# Molecular and cellular composition changes after neoadjuvant letrozole and palbociclib in early luminal breast cancer

**DOI:** 10.1016/j.xcrm.2025.102544

**Published:** 2026-01-09

**Authors:** Paul Cottu, Yann Kieffer, Jerome Lemonnier, Véronique D’hondt, Francois P. Duhoux, Céline Callens, David Gentien, Cécile Reyes, Anais Boulai, Isabelle Desmoulins, Marie-Ange Mouret-Reynier, Christelle Levy, Pierre-Etienne Heudel, Florence Dalenc, Julien Grenier, Laetitia Fuhrmann, Sylvain Baulande, Suzette Delaloge, Fatima Mechta-Grigoriou, Anne Vincent-Salomon

**Affiliations:** 1 Department of Medical Oncology, Institut Curie, Paris, France; 2 Université Paris Cité, Paris, France; 3 French Breast Cancer InterGroup UCBG, Research and Development Department, Unicancer, Paris, France; 4 IHU Institute of Women’s Cancer, Institut Curie, Paris, France; 5 Stress and Cancer Laboratory, Equipe labellisée par la Ligue Nationale Contre le Cancer, U830 Inserm, Institut Curie, PSL Research University, Paris, France; 6 Department of Medical Oncology, Institut du Cancer de Montpellier, Montpellier, France; 7 Department of Medical Oncology, King Albert II Cancer Institute, Cliniques Universitaires Saint-Luc and Institut de Recherche Expérimentale et Clinique (Pôle MIRO), UCL Louvain, Brussels, Belgium; 8 Genetics Unit, Pathology and Theranostic Medicine Department, Institut Curie, Paris, France; 9 Genomics Platform, Department of Translational Research, Institut Curie, Paris, France; 10 Department of Medical Oncology, Centre Georges François Leclerc, Dijon, France; 11 Department of Medical Oncology, Centre Jean Perrin, Clermont-Ferrand, France; 12 Department of Medical Oncology, Centre François Baclesse, Caen, France; 13 Department of Medical Oncology, Centre Léon Bérard, Lyon, France; 14 Department of Medical Oncology, Oncopole Claudius Régaud, IUCT, Toulouse, France; 15 Department of Medical Oncology, Institut Sainte Catherine, Avignon, France; 16 Pathology and Theranostic Medicine Department, Institut Curie, Paris, France; 17 ICGex – NGS Platform, Institut Curie Research Center, Paris, France; 18 Department of Cancer Medicine, Gustave Roussy, Villejuif, France; 19 PSL University, Paris, France

**Keywords:** luminal, palbociclib, neoadjuvant, PAM50, prognosis, randomized, cell type

## Abstract

The NeoPAL trial compares neoadjuvant letrozole-palbociclib (LP) with chemotherapy (CT) in 103 patients with high-risk, early luminal breast cancer. At surgery, the NanoString BC360 proliferation score and Ki67 expression are reduced in both arms, together with upregulation of immune-related signatures. Overall, there is very little difference in the changes observed with LP as compared to CT, even in signatures related to response to estrogen and CDK4/6 manipulation. Deconvolution of bulk RNA sequencing (RNA-seq) data reveals high content at baseline in cancer cells, immunosuppressive cancer-associated fibroblasts, FOXP3+ CD4^+^ regulatory T lymphocytes, and TREM2+ macrophages. In contrast, myoepithelial cells, normal-like fibroblasts, FOLR2+ macrophages, and SELL+ CD4^+^ T lymphocytes accumulate after treatment in both arms. A low ROR score is observed at surgery in 63.3% and 43.5% of patients in the LP and CT arms, respectively. No 3-year breast cancer-specific survival events are observed in these patients. These data provide a rationale for CT-sparing trials in this setting.

## Highlights

• We report here the translational analysis from the NeoPAL trial in luminal breast cancer
• Neoadjuvant letrozole-palbociclib yields similar proliferation reduction to chemotherapy
• Upregulation of immune signatures and stromal remodeling are similar in both arms
• Achievement of a low ROR score correlates with excellent survival

## Graphical abstract

**Figure:**
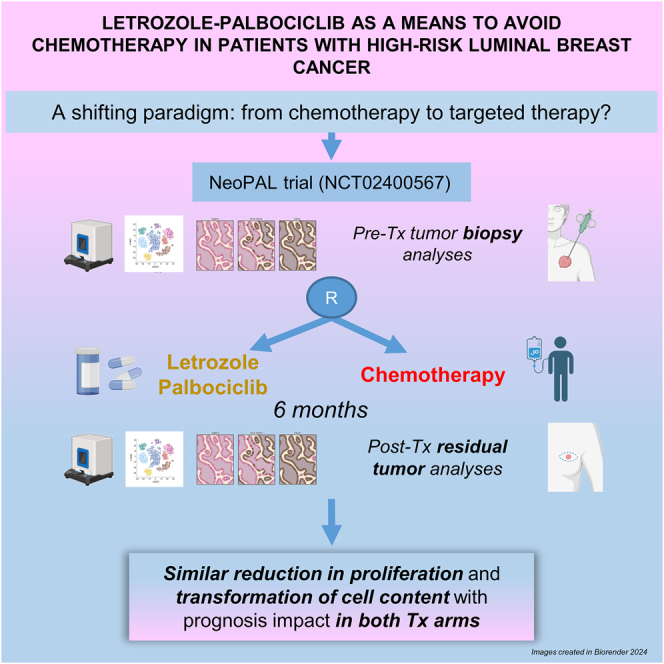

## Teaser

Cottu et al. report the translational analysis of the NeoPAL trial. They show that neoadjuvant letrozole-palbociclib and chemotherapy yield comparable reductions in proliferation, enhanced immune activity, and stromal remodeling in early luminal breast cancer. Low-ROR scores predict favorable survival. These data support chemotherapy-sparing approaches for patients with high-risk luminal disease.

## Introduction

Breast cancer is the most common cancer in women, with the hormone receptor-positive human epidermal growth factor receptor 2 (HER2)-negative subtype representing the most common subtype, usually referred to as luminal breast cancer (LBC).^1^ The standard of care for invasive LBC in the early stage combines local therapy (surgery and radiotherapy) and endocrine therapy in most patients.^2^ The generalization of genomic signatures has helped to refine the prognosis of early-stage LBC and the role of adjuvant chemotherapy (CT).^2^ Recently, innovative drugs such as cyclin-dependent kinases 4 and 6 inhibitors ([CDK4/6i] palbociclib, ribociclib, abemaciclib) have transformed the therapeutic landscape and the prognosis of advanced LBC,^3^^,^^4^ with early studies even suggesting that the combination of endocrine therapy and CDK4/6i may be as effective as CT in the advanced setting.^5^^,^^6^ Furthermore, abemaciclib and ribociclib have been demonstrated to enhance the mid-term outcomes of patients with high-risk early-stage LBC when administered in conjunction with adjuvant endocrine therapy for a period of 2–3 years.^7^^,^^8^

The neoadjuvant setting facilitates accelerated drug development and clinical practice changes, as it provides the opportunity to rapidly assess the efficacy of new therapies by using surrogate endpoints for long-term outcomes such as pathologic response.^9^ Nevertheless, its prognostic value has been challenged,^10^ and in LBC, more endocrine-specific endpoints such as the preoperative endocrine prognostic index (PEPI) scores^11^ or proliferation reduction^12^ may be more relevant. To further explore the potential role of CDK4/6i in high-risk, early-stage LBC, combined with an innovative use of the PAM50 signature, two prospective trials were conducted comparing a combination of letrozole (an aromatase inhibitor) plus a CDK4/6i with conventional CT as neoadjuvant therapy in patients with high-risk, early-stage LBC. The CORALLEEN trial (NCT03248427) compared the letrozole-ribociclib combination with a conventional anthracycline-taxane sequential CT regimen.^13^ The results suggested that at least 50% of patients with high-risk, early-stage LBC can achieve a significant and similar genomic downstaging with both letrozole-ribociclib and CT, as captured by the PAM50 risk-of-recurrence (ROR) class, which includes the PAM50 genomic score in combination with tumor size and nodal status. The NeoPAL trial (NCT02400567) demonstrated that in patients with PAM50-defined high-risk LBC, the combination of letrozole and palbociclib resulted in a pathological response rate that was comparable to that of a similar CT regimen and potentially more impactful on PEPI score response and proliferation reduction.^14^ Both studies demonstrated that letrozole-CDK4/6i was associated with a significantly lower incidence of adverse events compared to CT.^14^^,^^15^

Neoadjuvant therapy also facilitates the investigation of the mechanisms underlying treatment response and resistance.^9^^,^^16^^,^^17^ Previous studies have indeed extensively explored the mechanisms of resistance to endocrine therapy and CDK4/6 inhibitors in combination.^18^^,^^19^ These mechanisms will not be discussed in this report. Here, we focus on the exploration of the biological effects of an endocrine therapy-CDK4/6i combination in comparison to those of CT in patients with localized estrogen receptor (ER)-positive HER2-negative BC. To this end, we conducted a prespecified translational analysis of the NeoPAL trial that aimed to elucidate the differential molecular and cellular landscape of treatment response to CT and letrozole-palbociclib, through a multidimensional biomarker analysis at sequential time points.

## Results

### Biological landscape at inclusion in the NeoPAL trial

**Figure 1:**
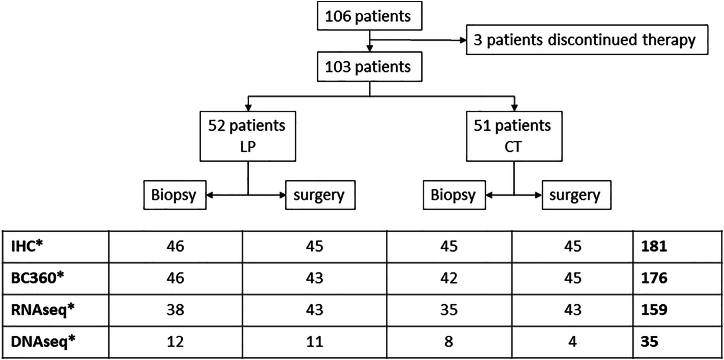
Flow chart Distribution of tumor samples for each analysis according to the treatment arm and the type of sample (baseline biopsy or surgical sample). ∗The figures in each cell indicate the number of available samples. BC360, NanoString BC360 transcriptional analysis; RNAseq, bulk RNA sequencing; DNAseq, targeted DNA sequencing.

The details and core results of the NeoPAL trial have been previously reported.^14^^,^^20^ Briefly, 106 patients with high-risk stage II–IIIA LBC (defined by central PAM50 subtyping either as luminal A, node positive or as luminal B) were randomized to receive either letrozole plus palbociclib 125 mg daily on a 3 weeks on/1 week off schedule (LP) for 19 weeks or CT (three cycles of epirubicin-cyclophosphamide followed by three cycles of docetaxel). Surgery was performed in 103 patients, 24 h after the last palbociclib dose and 3 weeks after the last cycle of CT. The primary endpoint was residual cancer burden (RCB). RCB 0 was observed in 3.8% of patients in the LP arm and in 5.9% of patients in the CT arm. RCB 0–I was observed in 7.7% of patients in the LP arm (95% confidence interval [CI], 0.4–14.9) and in 15.7% of patients in the CT arm (95% CI, 5.7–25.7). In addition, 17.6% and 8.0% of patients, respectively, achieved a breast cancer-specific survival PEPI score of 0.^14^ For the purpose of the present report, we studied 103 patients with 206 tumor samples, including the pre-treatment biopsies and the post-treatment surgical tumor samples. A total of 181, 176, 159, and 35 samples were available for immunohistochemistry (IHC), NanoString BC360 analysis, bulk RNA sequencing (RNA-seq), and targeted DNA sequencing, respectively (Figure 1), representing 91, 88, 86 and 21 patients, respectively. All analyses were conducted at Institut Curie, with the exception of the BC360 analysis, which was carried out at NanoString.

**Figure 2:**
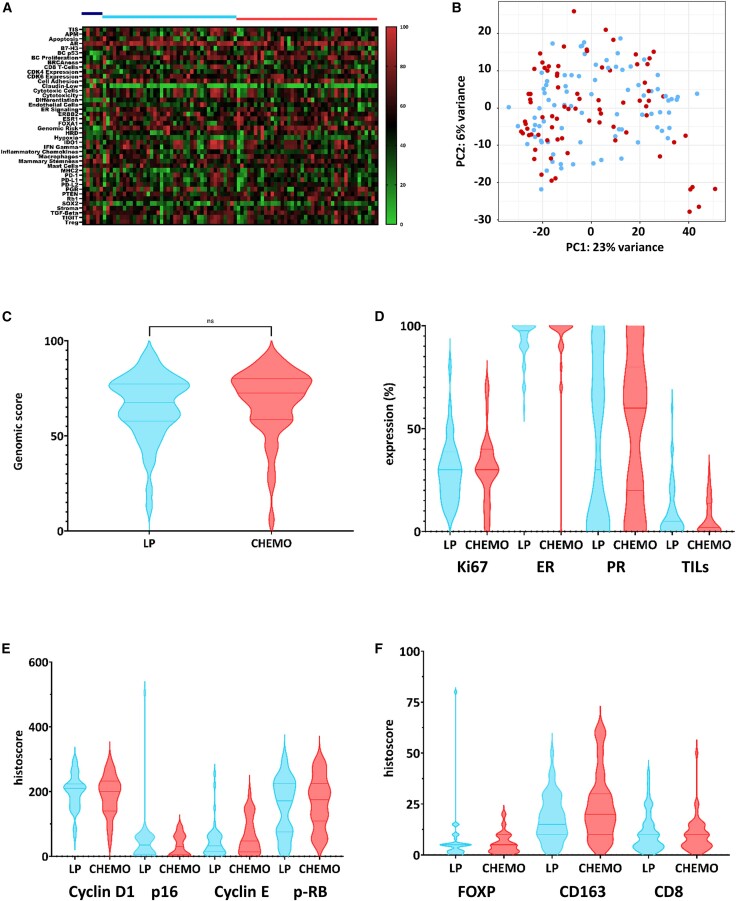
Multidimensional comparison of the populations of the two arms at entry into the study (A) Heatmap based on the BC360 normalized values of expression signature scores. The blue and red bars above the heatmap indicate patients in the letrozole+palbociclib and in the chemotherapy arms, respectively (dark blue, luminal A tumors; light blue, luminal B tumors; there were only luminal B tumors that were analyzed in the chemotherapy arm). (B). Principal-component (PC) analysis based on the 1,000 most variant genes by bulk RNA sequencing after variance-stabilizing transformation. Blue dots indicate the patients in the letrozole+palbociclib arm and red dots indicate the patients in the chemotherapy arm. *x* axis: PC1, 23% of variance; *y* axis: PC2, 6% of variance. (C) Distribution of the PAM50 genomic score at baseline in both arms. The horizontal bars show the median value with interquartile range. (D) Expression (in % of stained tumor cells) of Ki67, ER, progesterone receptor (PR), and percentage of TILs in baseline biopsies according to central review in both arms. The horizontal bars show the median value with interquartile range. Overall, there were only four luminal A tumors in the IHC analysis. (E). Histoscores of cyclin D1, p16, cyclin E, and phospho-Rb (p-Rb) expression at baseline according to central review in both arms. The horizontal bars show the median value with interquartile range. (F) Histoscores of FOXP3, CD163, and CD8 expression at baseline according to central review in both arms. The horizontal bars show the median value with interquartile range. All comparisons (D, E, and F, not shown) are exploratory and were not significant. CHEMO, chemotherapy arm. In the violin and box-and-whiskers plot, data are presented from min to max value, the middle bar is the mean, and the upper/lower bars or the boxes represent the 25%–75% interquartile range.

Key biomarkers (PAM50 subtyping, genomic risk, and RCB response) were similar between these four subsets and similar to the whole population of the trial (Table S1). The numerically lower proportion of patients with RCB 0–I in the 91-patient IHC subset was attributable to the lack of residual tumor tissue available for IHC analysis. We further examined the molecular pattern of the tumors at baseline. The distribution of the gene expression profile derived from the 42 biological signatures of the BC360 panel showed no significant difference between the treatment arms (Figure 2A). This was confirmed by principal-component analysis based on the 1,000 most variant genes derived from bulk RNA-seq, which demonstrated that the overall pattern of tumors from the LP arm (Figure 2B, blue dots) was indistinguishable from that of tumors from the CT arm (Figure 2B, red dots). Specifically, the BC360 genomic score (range: 0 to 100) at baseline was assessed in 88 available samples. The median scores were 67.5 and 72.5 in the LP and CT arms, respectively (*p* = 0.29; Figure 2C). Extensive IHC analysis including ER, PR, and Ki67 staining and tumor-infiltrating lymphocyte (TIL) counts (Figure 2D), cell cycle biomarkers (cyclin D1, p16, cyclin E, p-Rb; Figure 2E), and immune cell biomarkers (FOXP3, CD163, CD8; Figure 2F) also confirmed the similarity of the overall tumor profiles in both arms.

### Decrease in proliferation and changes in cancer cells

**Figure 3:**
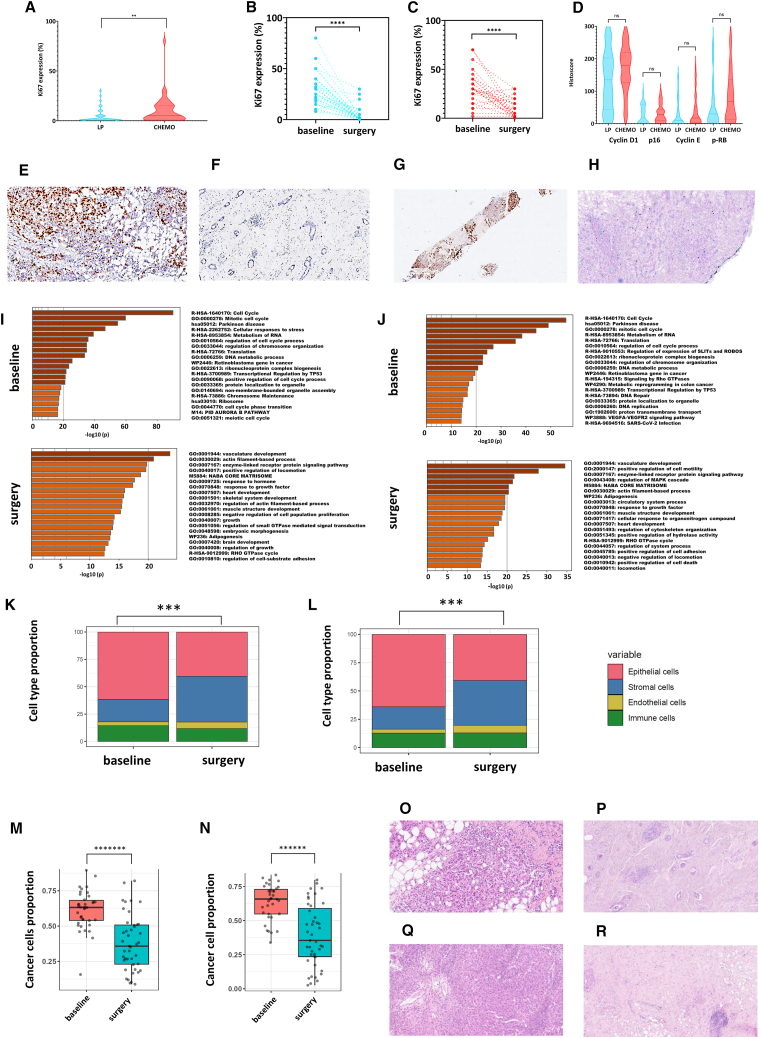
Analysis of the proliferation response, changes in gene expression profile and cell composition in the letrozole+palbociclib and in the chemotherapy arms (A) Final Ki67 expression as per central review, in the letrozole+palbociclib and in the chemotherapy arms. Exploratory comparison showed a significantly greater decrease in Ki67 expression in the LP arm than in the CT arm. (B and C) Individual decrease in Ki67 expression (*y* axis: %) after treatment with letrozole and palbociclib (B) and after treatment with chemotherapy (C). (D) Histoscore of cyclin D1, p16, cyclin E, and p-RB in surgical specimens as per central review. All exploratory comparisons were not significant except for cyclin D1 where it was of borderline significance (*p* = 0.051). (E and F) Example of Ki67 expression in the LP arm at baseline (E) and at surgery (F), showing the extinction of proliferation. (G and H) Example of Ki67 expression in the CT arm at baseline (G) and at surgery (H), showing the near-complete extinction of proliferation. (I and J) Gene expression analysis from bulk RNA-seq showing the gene ontology annotation associated with the 1,000 most upregulated genes at baseline (top) and at surgery (bottom) in the letrozole+palbociclib arm (I) and in the chemotherapy arm (J). (K and L) Bar graphs showing the decrease in the epithelial cell proportion from baseline to surgery, as captured by bulk RNA-seq and cell type deconvolution, in the letrozole+palbociclib arm (K) and in the chemotherapy arm (L). (M and N) Bar graphs showing the decrease in the proportion of cancer cells, as captured by bulk RNA-seq and cell type deconvolution, from baseline to surgery in the letrozole+palbociclib arm (M) and in the chemotherapy arm (N). (O and P) Example of tumor cells and TIL content at baseline in the LP arm (O). Decrease of tumor cell density and modification of TIL content at surgery (P). (Q and R) Example of tumor cells and TIL content at baseline in the CT arm (Q). Decrease of tumor cell density and modification of TIL content at surgery (R). *p* values are presented as asterisk (∗∗*p* < 0.01; ∗∗∗*p* < 0.001; ∗∗∗∗*p* < 0.0001; ∗∗∗∗∗∗*p* < 0.000001; ∗∗∗∗∗∗∗*p* < 0.0000001; ns, non-significant). In the violin and box-and-whiskers plot, data are presented from min to max value, the middle bar is the mean, and the upper/lower bars or the boxes represent the 25%–75% interquartile range.

We observed a lower median Ki67 expression at surgery in the LP arm compared to the CT arm (1%, interquartile range [IQR], 0–5 vs. 5%, [IQR, 2–15], *p* = 0.0001) (Figure 3A); final Ki67 in both arms (Figures 3B and 3C), individual decrease in Ki67 expression in the LP and CT arms, as confirmed by IHC analysis (Figures 3E–3H); and a higher frequency of complete cell-cycle arrest (i.e., final Ki67 expression ≤2.7%) in patients treated with LP (33 of 45, 73.3%) compared to CT (15 of 45, 28.9%; *p* < 0.0001). Consistent with these results, expression of key cell cycle proteins (P16, TP53, CCND1, CCNE1, and p-Rb) showed a similar pattern after treatment in both arms (Figure 3D). We then examined the differential gene expression profile derived from bulk RNA-seq at baseline and following treatment with LP (Figure S1A) and CT (Figure S1B). The overall pattern was again strikingly similar, as confirmed by the differential gene expression analysis between the two arms on the surgical samples. Only 37 genes were found to be differentially expressed with no relevant biological insight (Figure S1C, blue dots; Table S2; Figure S1D). A further analysis of the 1,000 most upregulated genes at baseline revealed that in both arms the most frequently involved biological processes were related to cell cycle, translation, and ribonucleoprotein biogenesis (Figure 3I, LP arm; Figure 3J, CT arm). These processes appeared to be almost extinguished at the time of surgery and replaced by vasculature development, extracellular matrix (ECM) organization, and cell motility processes (Figure 3I, LP arm; Figure 3J, CT arm). These alterations in gene expression were further investigated using the BC360 panel. Beyond the strong reduction of the genomic risk signature in both arms, we clearly observed very close patterns in both arms. For example, there was a significant decrease in the breast cancer proliferation signature, estrogen signaling-related signatures (e.g., ER signaling, ESR1, PGR, FOXA1), or differentiation signatures (Figure S1E, LP arm; Figure S1F, CT arm). Conversely, a parallel increase in signatures associated with the tumor microenvironment (e.g., endothelial cells, stroma, CD8 T cells, mast cells) was observed, suggesting a similar inflammatory response to therapy, whether it was the LP combination or CT (see below). Interestingly, an increase in the CDK6 expression signature, a potential mechanism of resistance to CDK4/6 inhibition, was also observed but not restricted to the LP arm. An illustrative example of how these biologically profound changes can be observed in a single patient is presented in Figure S1. The wheel plots show the collapse of the genomic risk signature from the baseline sample (Figure S1G) to the surgical sample, as well as the sharp decrease in the ER signaling signature or the breast cancer proliferation and Treg signatures (Figure S1H, arrows). Of note, targeted DNA sequencing of a 571-gene panel in a small subset of patients (due to tissue exhaustion) did not identify any mutation specifically associated with either response or resistance to treatment (Figure S6). No novel mutations were identified in the LP therapy group, as all matched samples exhibited identical mutation patterns (Figure S6).

We finally investigated how these intense proliferation and gene expression changes relate to changes in tumor cell composition. To this aim, the BayesPrism algorithm^21^ was applied to deconvolute bulk RNA-seq data and infer the composition of cell types and states in each tumor sample. A previously published BC cell atlas of 73,426 cells representing 39 different cell types and states was used as prior information.^22^ Global analysis of the main cell lineage fractions showed similar changes induced by neoadjuvant therapy, with no significant difference between LP and CT arms. As shown in Figure 3K (LP arm) and 3L (CT arm), two main changes in tumor cell proportions were induced by neoadjuvant therapy. First, there was a slight increase in the proportion of endothelial cells, consistent with the upregulation of gene signatures characteristic of vasculature development and endothelial cells. More strikingly, there was also a notable decline in the proportion of epithelial cells in favor of stromal cells in both arms (LP arm, *p* = 0.005; CT arm, *p* = 0.004). Interestingly, this decrease in epithelial cell proportions appeared to be primarily driven by the decrease in cancer cell proportions (Figure 3M, LP arm, *p* = 1.6e−07; Figure 3N, CT arm, *p* = 1.5e−06), demonstrating the equal efficacy of LP compared to CT in modifying tumor cell content. Individual pathological analyses confirming these results are shown in Figure 3O. Details of the changes in all cell types analyzed by bulk RNA-seq and BayesPrism deconvolution are provided in Table S3 and in the text below.

### Remodeling of the tumor microenvironment

**Figure 4:**
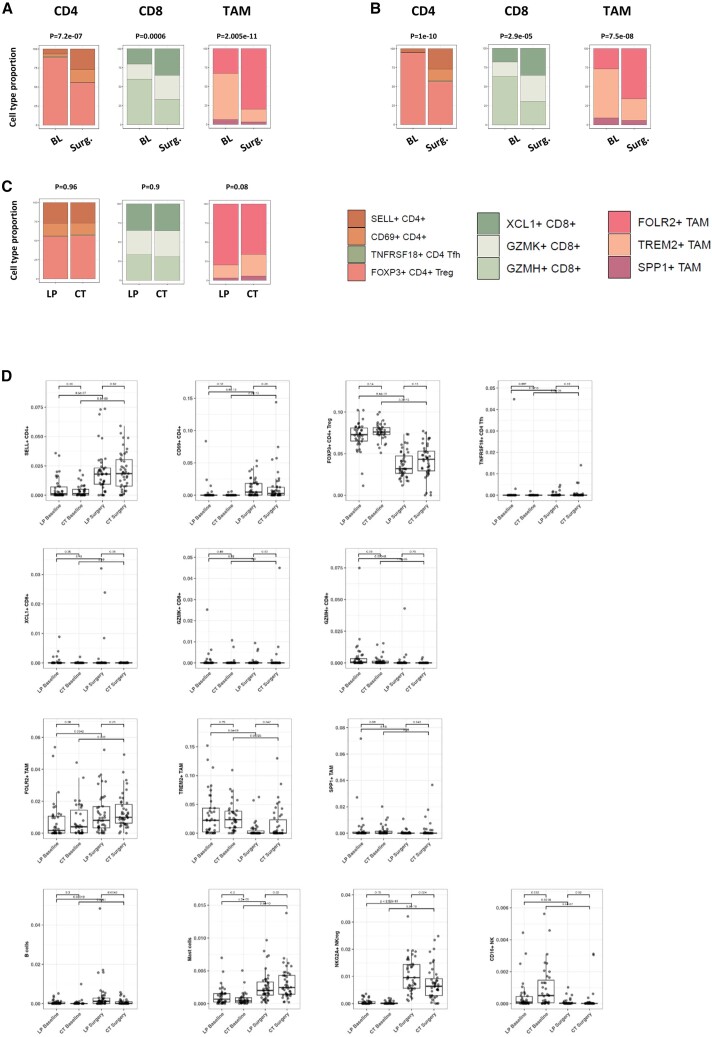
Capture of immune cell attraction by bulk RNA-seq and cell type deconvolution after neoadjuvant letrozole+palbociclib or chemotherapy (A and B) Changes in the CD4^+^ cell, CD8^+^ cell, and tumor-associated macrophage profiling between baseline and surgery, in the letrozole-palbociclib (A) and in the chemotherapy (B) arms. (C) Comparisons of the immune cell proportion profiling at surgery between the letrozole-palbociclib and the chemotherapy arms. (D) Detail of immune cell deconvolution before and after treatment in both arms. Each boxplot shows the variation of the indicated cell type at baseline and at surgery. All *p* values are indicated in Table S3. CD4, CD4^+^ T cells; CD8, CD8^+^ T cells; BL, baseline; Surg., surgery; SELL, Selectin L; TNFRSF, tumor necrosis factor superfamily receptor; Tfh, follicular helper T cell; XCL1, lymphotactin; GZM, granzyme; FOLR, folate receptor; TREM, triggering receptor expressed on myeloid cells; SPP, secreted phosphoprotein; NKG2A, killer cell lectin-like receptor G2A. In box-and-whiskers plot, data are presented from min to max value, the middle bar is the mean, and the upper/lower bars or the boxes represent the 25%–75% interquartile range.

**Figure 5:**
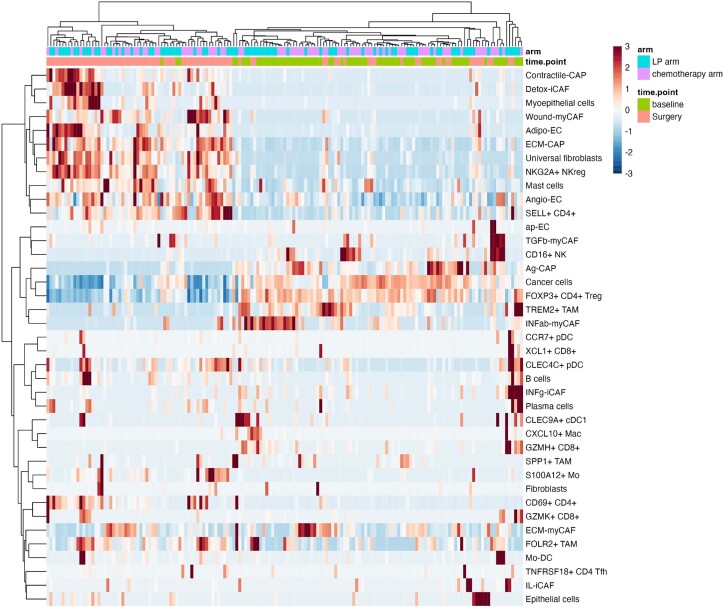
Global cellular composition of the 159 samples The heatmap was obtained through unsupervised analysis after deconvolution of bulk RNA-seq. Each row represents a cell type, and each column, a single tumor sample. The samples are evenly distributed by treatment arm (letrozole-palbociclib, in turquoise blue; chemotherapy, in light purple), but they appear separated by time point (baseline biopsy, in green, versus surgical sample obtained after neoadjuvant therapy, in pink). Before treatment (biopsy): there is an enrichment in cancer cells, IFNab-myCAF, IFNg, Ag-CAP, FOXP3+ CD4^+^ T cells, TREM2+ TAM, and GZMH+ CD8^+^. After treatment (surgery), there is an enrichment in myoepithelial cells, universal fibroblasts, detox-iCAF, wound-myCAF, endothelial cells, contractile-CAP, ECM-CAP, FOLR2+ TAM, NKreg, SELL+ CD4^+^, CD69^+^ CD4^+^, and mast cells. IL-iCAF, interleukin-producing iCAF; IFNg-iCAF, interferon gamma-secreting iCAF; ECM-myCAF, ECM-producing myCAF; TGFb-myCAF, tumor growth factor beta-producing myCAF; INFab-myCAF, interferon alpha/beta-producing myCAF; contractile-CAP, contractile cancer-associated perivascular-like fibroblasts; ECM-CAP, ECM-producing CAP; Ag-CAP, antigen CAP; ap-EC, antigen-presenting endothelial cells; angio-EC, angiogenesis EC; adipo-EC, adipogenesis-related EC.

We next investigated the changes in tumor cell composition induced by neoadjuvant therapy. Of note, the BC360 stroma signature was activated after treatment in both arms (Figures S1E and 2F). A highly significant increase in the proportions of naive (SELL+) and tissue-resident memory (CD69^+^) CD4^+^ T lymphocytes was observed, whereas there was a decrease in FOXP3+ regulatory T cells. This was observed in both the LP (*p* = 7.2e−07, Figure 4A) and CT (p = 1e−10, Figure 4B) groups (and confirmed by IHC analysis as shown in Figure S2). Details of the changes for each immune cell subpopulation and for each arm of treatment are illustrated in Figure 4D, with specific *p* values provided in Table S3. There was no discernible difference between treatment arms in the proportions of CD4^+^ T lymphocytes at surgery, indicating a similar effect of LP and CT on CD4^+^ T cell populations (*p* = 0.96, Figure 4C). Overall, the CD8^+^ T cell composition changed significantly with neoadjuvant therapy. There was an increase in precursor CD8^+^ T cells (XCL1+), as opposed to a decrease in differentiated (exhausted, GZMH+) CD8^+^ T lymphocytes, with both LP (*p* = 0.0006, Figure 4A) and CT (*p* = 2.9e−5, Figure 4B). Very interestingly, these changes were similar between LP and CT (*p* = 0.9, Figure 4C) and appeared to be primarily driven by the decrease in exhausted T cells (Figure 4D; Table S3), which are often associated with inefficient cancer control. Finally, we observed an increase in FOLR2+ TAM (tumor-associated macrophage) content, while there was a decrease in TREM2+ TAM, with overall highly significant changes in the TAM cell population, either with LP (*p* = 2.005e−11, Figure 4C) or CT (*p* = 7.5e−08, Figure 4B). Although there was no significant difference in the changes in TAM proportions achieved with either LP or CT (*p* = 0.08, Figure 4C), there was a numerical imbalance suggesting a more profound effect of the LP combination over CT on TREM2+ TAM content reduction (Figure 4C; Table S3). Finally, our bulk RNA-seq and cell deconvolution approach revealed important changes in other immune cell populations present at very low levels. In both treatment arms, there was a significant increase in the proportion of B cells, mast cells, and NKG2A^+^ NK (natural killer) cells, while there was a decrease in CD16^+^ NK cells (Figure 4D; Table S3). This remarkable expansion in cytotoxic immune cells was confirmed by the significant positive fold change in immune-related signatures in the BC360 analysis (Figure S2E, LP arm; Figure S2F, CT arm). Details of the fold change for each individual signature are presented in Table S4 (Mendeley dataset, key resources table). Some illustrative examples are depicted in Figure S3 (Figure S3A, LP arm; Figure S3B, CT arm). Importantly, the reduction in FOXP3+ T regulators observed in the LP arm was confirmed by the IHC analysis (Figure S2). The global cellular composition is depicted in Figure 5. The samples can be distinguished by each time point, with the baseline biopsy and surgical sample clearly separated. Conversely, there was no separation by treatment arm.

**Figure 6:**
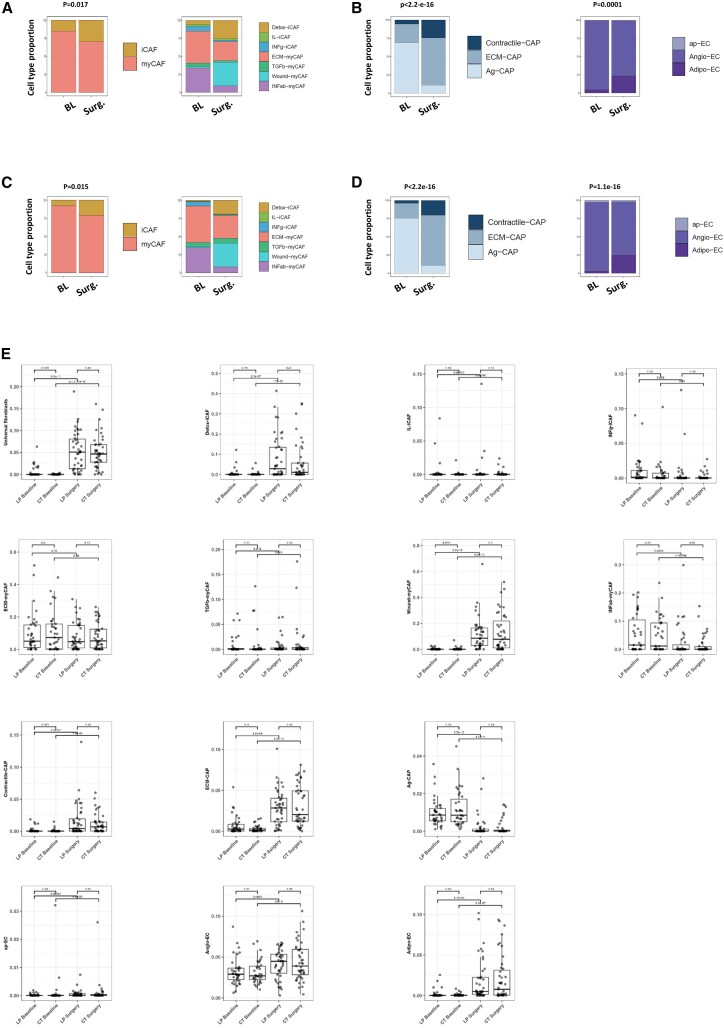
Stromal changes in non-immune cells by bulk RNA-seq and cell type deconvolution after neoadjuvant letrozole+palbociclib or chemotherapy (A and C) Bar graphs showing the pre- and post-treatment proportions of CAFs in the letrozole-palbociclib (A) and chemotherapy (C) arms, focusing on specific subpopulations of CAFs as captured by bulk RNA-seq and cell type deconvolution. After treatment, we observe a decrease in the ECM and IFNab-myCAF populations, while there is an increase in the detox-iCAF and wound-myCAF fractions. (B and D) Bar graphs showing the proportions of CAP cells and endothelial cells before and after treatment in the LP (A) and CT (C) arms, as captured by bulk RNA-seq and cell type deconvolution. After treatment (LP: B; CT: D), there is an increase in contractile-CAP and ECM-CAP (and a decrease in Ag-CAP), associated with an increase in adipo-EC. (E) Each boxplot shows the variation of the indicated cell type at baseline and at surgery. All *p* values are indicated in Table S3. In the box-and-whiskers plot, data are presented from min to max value, the middle bar is the mean, and the upper/lower bars or the boxes represent the 25%-75% interquartile range.

Beyond the remodeling in the immune cell profile after neoadjuvant therapy, cell deconvolution showed an overall decrease in fibroblasts (Table S3) and a specific enrichment in inflammatory cancer-associated fibroblast (CAF) (iCAF) in both treatment arms (LP arm, *p* = 0.017, Figure 6A; CT arm, *p* = 0.015, Figure 6C). According to our previously reported CAF atlas,^23^^,^^24^ a detailed examination of the iCAF and myofibroblastic (myCAF) subpopulations revealed a reduction in the content of ECM- and IFNαβ-myCAF clusters and an increase in the detoxification-associated iCAF (detox-iCAF) and wound-myCAF fractions (LP arm, Figure 6A; CT arm; Figure 6C). Cancer-associated pericyte-like cell (CAP) and endothelial cell profiles were also significantly altered by both treatments, as shown in Figure 6B (LP arm, *p* < 2.2e−16 and *p* = 0.0001, respectively) and Figure 6D (CT arm, *p* < 2.2e−16 and *p* = 1.1e−16, respectively). Indeed, both treatments increased contractile-CAP and ECM-CAP proportions, but decreased Ag-CAP. Moreover, a pronounced increase in Adipo-EC content was observed in the two treated arms to the expense of Angio-EC and Ag-EC. Details for each cell population and both arms of treatment are presented in Figure 6 and in Table S3. The remarkable similarity in the stromal response to either LP or CT is illustrated in Figures S4A, 4 and 5 (for all cell populations) and in Figure 6B (CAF response), Figure 6C (CAF subpopulations), Figure 6D (CAP cells), and Figure 6D (endothelial cells)

Finally, to gain a better understanding of the underlying mechanisms of response to neoadjuvant therapy, we performed an exploratory analysis of the correlation between the cell composition and treatment response, as measured by the RCB (the primary endpoint). Based on the similarity of the biological responses that were achieved with LP or CT, the two arms were combined for this analysis. As expected, a high RCB value was highly correlated with a high proportion of cancer cells at surgery, and also with a high proportion of FOXP3+ CD4^+^ regulatory T cells (Figure S5A). Conversely, high levels of contractile-CAP, Adipo-EC, and wound-myCAF were associated with a favorable RCB response (Figure S5B). This analysis was extended to our previously described eco-cell types (ECTs).^22^ Ten ECTs had been identified, corresponding to 11 cellular niches (Figure S5C). ECTs 1 and 7, which comprise cancer cells together with immunosuppressive cells and activating macrophages, were inversely correlated with low RCB at surgery. In contrast, ECT-4, mostly composed of protective immune and stromal cells, was positively associated with low RCB (i.e., a favorable response) (Figure S5D). Details of the correlation (Pearson) with RCB classes are given for each cell type and each treatment arm in Table S5. The correlation analysis between ROR and cell populations at surgery is shown in Table S6. Overall, “protective” cells, such as contractile-CAP, ECM-CAP, B cells, Adipo-EC, and detox-iCAF, all belonging to the ECT-1, were associated with a low-ROR favorable response, whereas cancer cells or FOXP3+ CD4^+^ T cells were associated with a high-ROR response.

Collectively, these results demonstrate, at the single-cell level as inferred from bulk RNA-seq analyzed with Bayesian deconvolution, the profound tumor remodeling achieved with neoadjuvant therapy in early LBC. They also strongly suggests that the well-tolerated combination of letrozole and palbociclib exerts strikingly similar effects to CT in this clinical setting.

### Genomic response and prognosis

**Figure 7:**
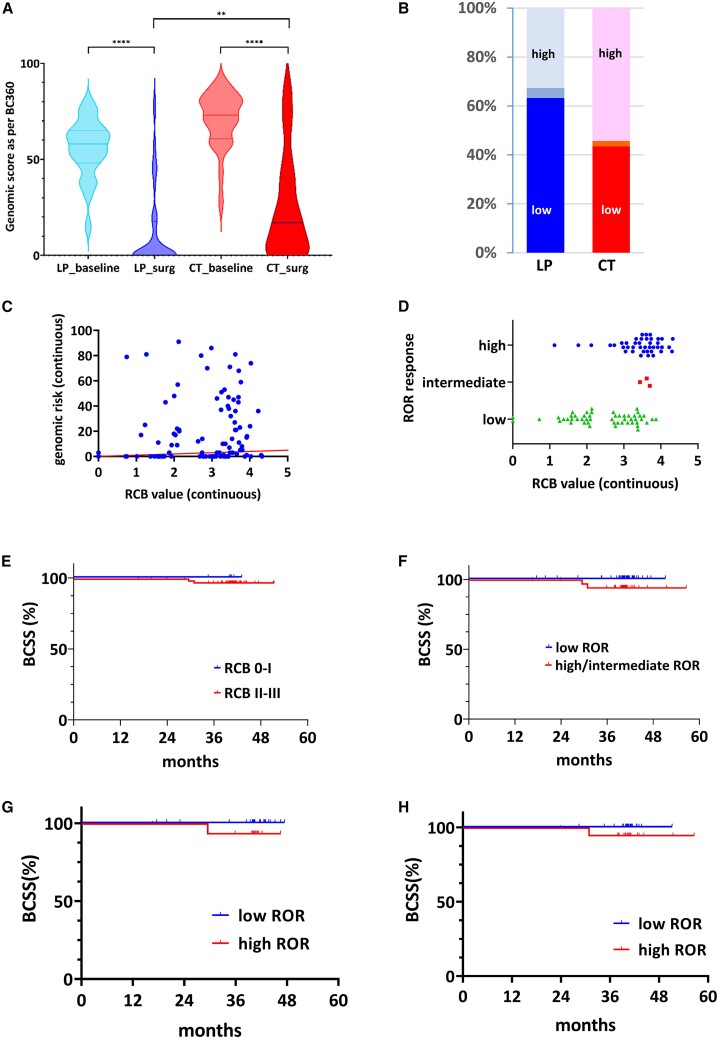
Genomic response, decrease of the ROR (risk of recurrence) score, and prognosis (A) Genomic response, as determined by the BC360 genomic score and the final ROR value and defined as the variation in the genomic score value between baseline and surgery, in the LP and CT arms. “Baseline” refers to the score value before any treatment, while “surg” refers to the score value at surgery, after neoadjuvant therapy. A highly significant decrease is observed in both arms. The exploratory comparison between the LP and the CT arms suggests a greater decrease of the genomic score in patients treated with the LP combination. (B) Bar graph showing the ROR response, as defined by the genomic score and the residual tumor. The proportion of “low ROR” responses is shown in the dark lower part of the bars (LP, dark blue; CT, dark red). Medium blue, intermediate ROR score response; light blue (LP) and light red (CT), high ROR response. (C) Spearman correlation showing a weak but significant relationship between the genomic risk (a component of the ROR response) and the RCB (as a continuous variable). The red line is the identity line. (D) Scatterplot showing the lack of relationship between RCB (the original primary endpoint of the study) and the ROR response. (E) BCSS probability according to RCB response (all patients). Blue curve, RCB class 0–I; red curve, RCB class II–III. (F) BCSS probability according to ROR response (all patients). Blue curve, “low ROR” response; red curve, “high ROR” response. (G) BCSS in the letrozole-palbociclib arm, according to ROR response. (H) BCSS in the chemotherapy arm, according to ROR response. ROR, risk of recurrence*. p* values are presented as asterisk (∗∗*p* < 0.01; ∗∗∗∗*p* < 0.0001; ns, non-significant).

As RCB may not be the most appropriate assessment criterion for the LP combination in the neoadjuvant setting, and more generally for neoadjuvant therapies in LBC, we finally investigated the potential value of the PAM50-based ROR at surgery as a response marker and prognostic factor, defining the genomic response to neoadjuvant therapy. In the present analysis, we used the final ROR, as assessed on the surgical sample after neoadjuvant therapy, similarly to the CORALLEEN trial.^13^ The genomic score was significantly lower at surgery in both arms (*p* < 0.0001 for each arm, Figure 7A) compared to baseline. An exploratory comparison suggested that it was even lower in the LP group than in the CT group after treatment (*p* = 0.0064, Figure 7A). A numerically higher proportion of patients with a low ROR was observed at surgery in the LP arm (31 of 49 evaluable patients, 63.3%) than in the CT arm (20 of 46 patients, 43.5%), although not significant (*p* = 0.10, Figure 7B). A subsequent examination of the potential correlation between ROR and RCB for all patients revealed a rather limited correlation (r Spearman = 0.21; 95% CI, 0.0085–0.395; *p* = 0.0358) as shown in Figure 7C. Indeed, patients with a low ROR were evenly distributed across the RCB values (Figure 7D).

We then evaluated the prognostic performance of RCB and ROR in the whole population. As very few patients had an RCB 0 response, we pooled patients with an RCB 0–I response and compared them with those with an RCB II–III response. No significant difference in breast cancer-specific survival (BCSS) was observed, despite a numerical difference favoring patients with an RCB 0–I response with a hazard ratio = 0.33 (95% CI, 0.0024–47.09; *p* = 0.66) (Figure 7E). An almost significant BCSS benefit was observed in patients who achieved a low-ROR response with no events after a minimum per-protocol follow-up of 3 years (hazard ratio [HR] = 0.08; 95% CI, 0.005–1.43; *p* = 0.087; Figure 7F). These results were confirmed when looking at the two treatment arms separately (Figure 7G, LP arm: HR = 0.06, *p* = 0.19; Figure 7H, CT arm: HR = 0.11, *p* = 0.27). Taken as a whole, these results strongly suggest the biological and clinical validity of ROR at surgery as a relevant endpoint for neoadjuvant treatment with endocrine-CDK4/6i therapy in patients with early LBC.

## Discussion

This study further advances the understanding of how the letrozole-palbociclib combination or cytotoxic CT exerts its effect on LBC at the single-cell-like level and molecular levels. Few single-cell approaches have been reported so far. Most of these studies used cell line analyses and focused on resistance to CDK4/6 inhibition, suggesting miscellaneous mechanisms of resistance.^25^^,^^26^^,^^27^ Indeed, conducting single-cell analyses on biopsy samples in clinical practice is highly challenging at the moment. Our Bayesian deconvolution strategy for bulk RNA-seq data is robust and enables us to investigate the cell-type composition of tumors using formalin-fixed, paraffin-embedded samples available in clinical practice. Some reports further analyzed the previously reported FELINE trial,^19^ adding GWAS or bulk omics data, again pointing miscellaneous mechanisms.^27^^,^^28^ One study specifically focused on the comparative analysis of the pre- and post-treatment with CDK4/6 inhibitor profile.^29^ Albeit conducted in the metastatic setting, this study very interestingly suggested that response to CDK4/6 inhibition is linked with changes in the immune cell composition, including an increase in CD8^+^ and NK cells. However, cell subpopulations were not analyzed. Here, we did not focus on the widely described mechanism of resistance to CDK4/6 inhibition, but we rather harnessed bulk RNA-seq data from 159 high-risk LBC tumor samples using BayesPrism deconvolution to determine cell composition before and after neoadjuvant therapy,^21^^,^^22^ according the treatment arm, an approach that was not used in the CORALLEEN trial.^30^ The use of cutting-edge deconvolution methods revealed that the composition and content in different cell types and states are identical when patients are treated with CT or endocrine plus CDK4/6i therapy. Indeed, the two types of treatments enhance infiltration of immunoprotective cells, reduce immunosuppressive CAF populations, and decrease cancer cell proliferation to a similar extent. Using single-cell transcriptomics and genomics, the atlas of human breast cells has been established, showing that it consists of epithelial cells, vascular cells, fibroblasts, and the complex category of immune cells.^31^^,^^32^ Spatial profiling technologies have helped to decipher the precise cell composition in cancer and describe heterotypic interactions.^33^ Notably, randomization ensured that even in this limited sample, molecular and cellular characteristics were identical between the two arms of the trial. In addition to the significant reduction in the proportion of cancer cells, we demonstrate that the treatment induces notable changes in the immune cell profile.

A significant increase in protective CD4^+^ T cells (SELL+, CD69^+^) was observed, in contrast to the decrease in immunosuppressive regulatory T cells (FOXP3+) and exhausted CD8^+^ T cells (GZMH+). This was corroborated by the elevated expression of immune signatures in the BC360 panel and by other similar observations indicating an upregulation of immune pathways after exposure to a CDK4/6i in both luminal and triple-negative breast cancer, including in the CORALLEEN trial.^30^^,^^34^ Most interestingly, we also observed important changes in the composition of TAMs. TAMs are flexible immune cells that may promote activation of dormant disseminated tumor cells and, more generally, tumor evolution,^35^^,^^36^^,^^37^ possibly through suppression of cytotoxic T cells and NK activities.^37^ However, this global view has been reconsidered by the identification of specific TAM subpopulations. Namely, FOLR2+ macrophages have been identified as tissue-resident macrophages present in both healthy and malignant luminal breast tissues. FOLR2+ TAMs have been shown to localize near vessels within the tumor stroma and to colocalize with CD8^+^ T cells.^38^ Moreover, this colocalization correlates with a favorable outcome. Here, we observed a significant enrichment of the FOLR2+ TAM subset after neoadjuvant therapy, together with a relative increase in CD8^+^ cytotoxic T cells. Conversely, we observed a significant decrease in TREM2+ macrophages (which often include SPP1+ macrophages), which have been repeatedly associated with immunosuppression in various cancer types, including breast cancer.^39^ In addition to the aforementioned global improvement in immune attraction, we observed an increase in B cells, mast cells, and especially NKG2A+ NK cells, suggesting a maintained capacity in NK cell expansion,^40^ whereas there was a decrease in CD16^+^ NK cells, which are more involved in tumor-targeting monoclonal antibody-based activity.^41^

CAFs represent one of the most prevalent cell types within the tumor microenvironment.^24^^,^^42^^,^^43^ Several CAF populations with specific functions have now been evidenced, including CAF with myofibroblastic (myCAF), inflammatory (iCAF), and antigen-presenting (apCAF) properties.^23^^,^^24^^,^^43^ Most of these subpopulations belong to the FAP+ CAFs, which have been generally associated with immunosuppression and tumor promotion.^24^^,^^44^ Our group has demonstrated that the FAP+ CAFs are divided into 8 different clusters that are differentially associated to immune control.^23^ In the present series, we observed an enrichment of specific CAFs (detox-iCAF and wound-myCAF) associated with favorable immune response.^22^ From a broader perspective, it is now recognized that the three main cell categories of the tumor microenvironment (vascular cells, immune cells, fibroblasts) are engaged in complex interplays that control tumor evolution.^22^^,^^45^^,^^46^ We have defined 10 cellular niches called EcoCellTypes, consisting of specific subsets of each of the three main cell categories and which are differentially localized in breast tumors.^22^ We observed an impressive correlation between treatment efficacy and immunocompetent EcoCellType, again demonstrating the complexity of the mechanisms of response to neoadjuvant therapy and the remarkable similarity between LP and CT.

The randomized phase 2 NeoPAL and CORALLEEN trials have pioneered the comparison of a well-tolerated endocrine therapy-CDK4/6i combination with conventional CT as neoadjuvant therapy in patients with high-risk early LBC.^13^^,^^14^ The results of both trials strongly support the use of the endocrine therapy-CDK4/6i combination over CT, as it produced very similar pathological results^13^^,^^14^ and favorable survival outcomes.^20^ Relevant endpoints for endocrine therapy-based neoadjuvant treatment have been debated. The RCB score was developed for neoadjuvant CT, and its prognostic performance is lower in LBC than in other subtypes.^9^^,^^16^ Alternative approaches have been developed to better evaluate the efficacy of endocrine therapy-based neoadjuvant treatment, mostly based on Ki67-based assessments of proliferation in the surgical specimen. Decrease in the Ki67 expression after neoadjuvant endocrine therapy has strong prognostic value,^12^ enhanced by the combination with estrogen receptor expression and final TNM stage in the PEPI scores.^11^ The long-term prognostic value of the ROR risk class has been widely demonstrated in early-stage LBC.^47^^,^^48^^,^^49^^,^^50^ Beyond the dramatic decrease in proliferation under LP therapy, which has been confirmed in other studies such as NeoPalAna and CORALLEEN,^18^^,^^30^ the present expanded results of the NeoPAL trial demonstrate that the risk of recurrence (ROR), which combines the PAM50 genomic score and TNM staging, can be confidently applied to surgical samples following neoadjuvant treatment and used as a relevant endpoint in this specific clinical setting. We confirm here that the LP combination can achieve a high rate of favorable ROR responses, in line with the results obtained with ribociclib,^13^ another CDK4/6 inhibitor that has demonstrated its efficacy in both the advanced and early settings.^3^^,^^8^ Furthermore, ROR demonstrated superior performance as a prognostic indicator following neoadjuvant therapy in patients with LBC, either endocrine therapy-CDK4/6i combination or CT.

### Limitations of the study

We acknowledge some limitations in this report. It is important to note that this is a retrospective exploratory study with a limited sample size not powered to formally compare outcomes between study arms. The limited sample size may also have led to an underestimation of the differences between the LP and the CT arms, although the consistency of our results, regardless of the method used to investigate potential differences, lends robustness to our observations. The very low number of patients achieving an RCB 0 response has also limited the specific analysis of major responders. Additional stratification approaches, such as IntClust^51^ or Oncotype DX,^52^ could have been used in these analyses. But, as the majority of tumors were of the luminal B subtype in both arms, the stratification based on molecular classes of tumor cells was not enough relevant. Instead, we developed a clinically driven approach exploring both tumoral and microenvironment genes, using NanoString BC360 signatures to dynamically explore multidimensional features with a single test. The issue of resistance to LP therapy was not specifically analyzed; however, it has been explored elsewhere.^18^^,^^19^ DNA sequencing analysis was limited by the very small number of remaining samples available and demonstrated that the study population was representative of early invasive breast cancer as described by The Cancer Genome Atlas.^53^

In conclusion, based on balanced groups and using the neoadjuvant setting as a discovery platform, the present report suggests that the letrozole-palbociclib combination has a similar efficacy to CT according to multiple validated and innovative analyses such as cell-type composition identification from bulk RNA-seq Bayesian deconvolution in a clinical setting. This efficacy is supported by an intense remodeling of tumor cellular composition, which rewires the tumor microenvironment toward a more effective tumor immune control. To confirm these results in a larger scale and to further develop CT-sparing studies, we are currently conducting a phase 2 clinical trial, RIBOLARIS (NCT05296746), which has started enrolling and will evaluate ribociclib plus letrozole without CT in the *neo*/adjuvant setting for those patients who achieve an ROR-low status at surgery.

## Resource availability

### Lead contact

Further information and requests for resources and reagents should be directed to and will be fulfilled by the lead contact Dr. Paul Cottu, paul.cottu@curie.fr.

### Materials availability

Clinical data are available for access upon external requests. Applicants should contact the lead contact to request access to clinical data. The request will be discussed internally in the joint steering committee of the study. The decision will be communicated within 1 month from the request. Applicants must complete specific documents to be granted a user license. The study protocol is available in the supplemental information. This study did not generate new unique reagents.

### Data and code availability

• DNA and RNA sequencing data generated in this study have been deposited on the EGA portal, under the access number EGAD50000001490. BC360 data, RNA-seq pathway changes data, and immunohistochemistry data have been deposited in the Mendeley and Figshare deposits (respective access codes: DOI: https://doi.org/10.17632/2btzwwvyy5.1 and https://doi.org/10.6084/m9.figshare.30634493).
• Code: All analyses have been performed according to our previous reports.^22^^,^^23^
• Any additional information required to reanalyze the data reported in this work paper is available from the lead contact upon request.

## Acknowledgments

We thank the patients and their families as well as the investigators and staff involved in the NeoPAL trial. We are grateful to Unicancer for promoting the clinical trial and especially to J.L. who was in charge of the NeoPAL clinical trial and all the members of the NeoPAL team that led the monitoring of the clinical data and sample shipment. We are also grateful to Pfizer and NanoString for their enthusiastic support. 10.13039/100004319Pfizer provided the funding of the study. NanoString provided the PAM50 analysis for the clinical part of the trial and the BC360 panel analysis for the present translational study. Neither Pfizer nor NanoString was involved in the study design, the analyses of the data, or the writing of the manuscript.

Pascale Morel, formerly at NanoString, was instrumental in the conduct of the trial. We also deeply thank Benoît Albaud and Patrica Legoix from the genomics platforms at Institut Curie.

## Author contributions

P.C., F.M.-G., and A.V.-S. conceptualized the translational analysis of the NeoPAL trial. P.C. and S.D. were the principal investigators of the clinical part of the trial. P.C. coordinated the translational axis and was involved in recruitment, clinical care, and data returns. P.C. and Y.K. ran the statistical analyses of the study. Y.K. and F.M.-G. conceptualized and performed the bulk RNA-seq and cell deconvolution analyses. J.L. was the coordinator of the NeoPAL trial on behalf of UNICANCER and centralized the collected samples and data. V.D.H., F.P.D., I.D., M.A.M.-R., C.L., P.E.H., F.D., and J.G. all importantly contributed to patient accrual and sample availability. C.C. and A.B. performed the DNA sequencing analyses. D.G. coordinated the BC360 analyses and C.R. performed the NanoString experiments. L.F. and A.V.-S performed the IHC analyses. A.V.-S. interpreted all IHC stainings and evaluated the tumor cellularity for molecular analyses. P.C., Y.K., C.C., F.M.-G., and A.V.-S. wrote the initial manuscript. All authors approved the final manuscript and contributed to critical revisions of its intellectual content.

## Declaration of interests

P.C. received consultant or invited speaker fees from Pfizer, Lilly, Novartis, Menarini, Roche, and AstraZeneca. F.P.D. reports a postdoctoral research grant from Fondation Belge contre le Cancer; consulting fees from Amgen, AstraZeneca, Daiichi Sankyo, Eli Lilly, Gilead Sciences, Merck Sharp & Dohme, Novartis, Pfizer, Pierre Fabre, Roche, and Seagen (paid to his institution); and travel support from Amgen, AstraZeneca, Daiichi Sankyo, Gilead Sciences, Pfizer, Roche, and Teva.

## STAR★Methods

### Key resources table

**Table:** 

REAGENT or RESOURCE	SOURCE	IDENTIFIER
**Antibodies**		

CD8	BioSB	BSB 2845
CD163	Novocastra	RRID:AB_2920861
p16	Roche-Ventana	RRID:AB_2833231
CCND1	BioSB	BSB 3761
CCNE1	BioSB	BSB 6555
FOXP3	Abcam	RRID:AB_2104897
p-(SER807/811)-Rb	Cell Signaling	RRID:AB_836912
TP53	DAKO	RRID:AB_2206626

**Biological samples**		

Tumor samples	This study	N/A

**Deposited data**		

Sequencing data	This study	EGA D50000001490
Participant-level clinical data	This study	Available upon reasonable request (details in the text)
BC360 and IHC data	This study	Mendeley deposit Reserved DOI: https://doi.org/10.17632/2btzwwvyy5.1 Figshare deposit: https://doi.org/10.6084/m9.figshare.30634493
Pathway changes (RNAseq)	This study	Mendeley deposit Reserved DOI: https://doi.org/10.17632/2btzwwvyy5.1 Figshare deposit: https://doi.org/10.6084/m9.figshare.30634493

**Software and algorithms**		

R studio v4.0.0	N/A	https://www.r-project.org/
GraphPad Prism v10.2.0	N/A	https://www.graphpad.com/

### Experimental model and study participant details

#### Clinical trial design and patients

The details and core results of the NeoPAL trial have been previously reported.^14^^,^^20^ The Protocol is available in the Supplementary material. Per protocol, all patients were followed for at least three years. This academic study was sponsored by UNICANCER, conducted within the French Breast Cancer Intergroup (UCBG), and registered at clinicaltrials.gov (NCT02400567) as the NeoPAL study. The study, which received the approval of the regulatory authorities on 12 December 2014, was conducted in accordance with Good Clinical Practice guidelines and the Declaration of Helsinki. We obtained two written informed consents from all randomised patients (before enrollment and before randomisation after Prosigna test results and axillary lymph node characterisation) for participation in the clinical part of the trial. All patients provided a specific written informed consent for translational analyses. The study obtained full approval by a national French Ethic Committee (CPP Ile de France III, Paris, France) and by local IRB (UCL Saint-Luc) in Belgium. As per French regulations, gender, ancestry, race, ethnicity, and socioeconomic status of the participants were not collected.

### Method details

#### Data collection and biomarkers of clinical trial

##### Immunohistochemistry analyses

For each patient, two specimens corresponding to the biopsy before treatment and surgery after treatment were prospectively collected in the central lab. Formalin-fixed paraffin embedded (FFPE) blocks were cut in serial sections (3μm). All immunostainings were performed on the BOND RX fully automated stainer (Leica Biosystems). Each antibody and steps of immunohistochemistry’s protocol are described in the table below.

A referent breast pathologist (AVS) interpreted all the stainings, read on a microscope. External controls were associated in each batch. For each staining: the intensity of the staining, the percentage of positive cells, the staining localization (nuclear, cytoplasmic, and membranous) were assessed on the biopsy before treatment and the most representative block of the residual disease on the surgical specimen after treatment. Histoscores were derived when appropriate.

##### BC360 analyses

BC360 analytic steps were performed at the Institut Curie Genomics platform. Raw data were generated at the Institut Curie Genomics Platform.

Biological analyses were performed in collaboration with Nanostring (Data Analysis Service). The BC360 panels harnesses the nCounter technology on a 759 genes set to describe the biology of breast cancer through an array of RNA expression signatures. The general output for a given signature or pathway is a binary variable (“high” or “low”; “activated” or “non activated”) easily includable in the NeoPAL clinical database. Beyond the PAM50 molecular subtype signature, many pathways are of particular interest in the context of the NeoPAL trial, such as: ER signaling; apoptosis; tumor proliferation; stromal tissue abundance; immune signaling, etc.

Details on normalisation, and differential Expression Analysis are available on request.

##### PAM50 subtypes

PAM50 Subtype calls are the result of a three-step algorithm. The first step involves a scaling using two sets of scaling factors to bring the housekeeper and reference sample expression values into the scale necessary for the next step. This second step calculates the correlation between the observed scaled expression for the PAM50 genes and a centroid for each of the four subtypes resulting in a set of four correlation values for each sample. The remaining step is to identify the subtype correlation with the greatest value and set that subtype as the subtype call for that sample.

#### Genomic risk of recurrence (genomic risk)

Genomic Risk scores are the result of a multiple step algorithm. The first step involves a scaling using two sets of scaling factors to bring the housekeeper and reference sample expression values into the scale necessary for the next step. This second step calculates the correlation between the observed scaled expression for the PAM50 genes and a centroid for each of the four subtypes that is different than that for calling subtypes and results in a set of four correlation values for each sample. The next step is to calculate a proliferation score for each sample, followed by taking a weighted sum of the proliferation score and the four subtype correlations. This last score is then scaled to be between 0 and 100. No tumor size information is utilized, only the genomic information portion of ROR.

#### Quality control

We assessed the technical performance of the nCounter profiling assay in this study. First, housekeeper genes assess sample integrity by comparing the observed value versus a predetermined threshold for suitability for data analysis. The machine performance is assessed using percentage of fields of view that were attempted versus those successfully analyzed. The binding density of the probes within the imaging area, ERCC linearity, and limit of detection are used as readouts of the efficiency and specificity of the chemistry of the assay. Any sample deemed as failing any one of these QC checkpoints were removed from the analysis. All details on quality control (housekeeper genes signal, binding density, limit of detection of the assay) are available on request.

#### Bulk-RNAseq analysis and cell deconvolution

##### Processing of bulk RNA-seq

RNAs were extracted from six 10μm-thick sections using High Pure FFPET RNA Isolation Kit according to the manufacturer’s instructions.Concentration was assessed by using NanoDrop One (ThermoFisher). RNA sequencing libraries were prepared from variable amounts of total RNA ranging from 94 to 192 ng, using the QuantSeq FWD 3′mRNA Seq LEXOGEN kit (CliniSciences). 159 Libraries were prepared according to the manufacturer’s recommendations for FFPE samples. The first step enables the synthesis of double strand cDNA, by reverse transcription, using oligo dT priming. A final PCR step was applied, with a number of cycles ranging between 21 and 23. The resulting amplified and barcoded libraries were then individually quantified and qualified using Qubit fluorometric assay (Invitrogen) with dsDNA HS (High Sensitivity) Assay Kit and LabChip GX Touch using a High Sensitivity DNA chip (PerkinElmer). Libraries were then equimolarly pooled and quantified using a qPCR method (KAPA library quantification kit, Roche). The sequencing was carried out using single-read mode SR100 on an Illumina Novaseq 6000 instrument. The sequencing configuration was set to reach an average of 10 million reads per sample. cDNA libraries were prepared using the 3′ mRNA-Seq library Prep Kit followed by sequencing on NovaSeq (Illumina). Overall quality of raw sequencing data was checked using FastQC (v0.11.9). Reads were then aligned on a ribosomal RNA database using bowtie (2.4.2) and on the human reference genome (hg38) with STAR (2.7.6a). Additional controls on aligned data were performed to infer strandness (RSeQC 4.0.0), complexity (Preseq 3.1.1), gene-based saturation, read distribution or duplication level using Bioconductor R package DupRadar. The aligned data were then used to generate a final count matrix with all genes and all samples. Only genes with at least one read in at least 5% of all samples were kept for further analyses. Normalization and differential analysis were conducted with DESeq2 R package.

##### Pathway enrichment

Pathways enrichment tests were performed using Metascape (https://metascape.org) with default parameters using top 1000 differentially expressed genes identified with DESeq2 analysis (adjusted *p*-value <0.05).

##### Deconvolution of bulk RNA-seq data

159 Bulk RNA-seq from the NeoPAL cohort were deconvoluted using BayesPrism algorithm version v2.019. Raw count matrix of 73,426 high-quality cells from BC atlas was used as input for prior information. Labels were derived from the annotation of 39 cell types and states previously described20. Mitochondrial and ribosomal protein coding genes were removed as these genes are expressed at high magnitude and not informative in distinguishing cell types. MALAT1 and genes from chrX and chrY were also removed following indication from BayesPrism’s authors. To reduce batch effects and speed up computation, deconvolution was performed only using protein coding genes. Default parameters to control Gibbs sampling and optimization were used. Final estimation of cell type fraction in each bulk RNA-seq sample was recovered using the updated theta matrix and used for downstream analysis.

#### Heatmap and clustering

Heatmap showing the global cell type composition of the 159 bulk RNA-Seq from the NeoPAL cohort was performed using pheatmap R package. Hierarchical clustering was applied on both samples and cell types using Euclidean distance and ward.D2 method. Values were centered and scaled by cell types.

#### DNAseq analyses

Sequencing was carried out using the Agilent SureSelect CD Curie CGP panel of 571 genes on 14 paired tumor biopsy/surgery and 7 single biopsies DNA from the NeoPAL trial. About 50 ng of input frozen DNA was used to build the libraries using the Agilent SureSelect XT-HS library prep kit (Agilent Technologies, Santa Clara, CA). The pool was finally sequenced on a NovaSeq 6000 (Illumina Inc., San Diego, CA). Bioinformatic analysis and variant selection algorithm were performed as described previously.^54^ We considered a minimal allelic ratio of 5% and a maximal frequency in the population of 0.1% with a minimal coverage of 100 reads. For each variant selected according to the above criteria, paired sample was checked specifically for this abnormality which was conserved even if less than 5%. Amplifications and homozygous deletions were defined as DNA segments <10 Mb with a log2 median ratio higher than 2 or lower than −1.5, respectively taking into account tumor cell infiltration. Oncoprints were drawn using the ComplexHeatmap package and were performed with the Maftools package for 4.00 version of R.

#### Outcomes

The residual cancer burden response (RCB) was evaluated as previously reported.^14^

### Quantification and statistical analysis

All relevant data were included in the existing NeoPAL database hosted by UNICANCER and analyses were performed at Institut Curie. Conventional parametric and non-parametric descriptive and time-related statistics were used, including multivariate models when applicable. The Wilcoxon signed-rank test was used to compare matched tumors at baseline and surgery. *p* value are presented as asterisk (∗ means *p* < 0.05; ∗∗ means *p* < 0.01; ∗∗∗ means *p* < 0.001; ∗∗∗∗ means *p* < 0.0001).

#### Survival analysis

In the absence of a grouping variable, the survival analysis used to create the forest plot incorporates a Cox proportional hazards model with the survival outcome as a dependent variable and the observed normalized gene expression or signature score data as a continuous covariate in the model.

Survival(time, event) = μ+Expression_(gene or signature)+ ε

The analysis method is performed on a by gene or by signature basis, as appropriate, and uses the regression routines implemented in the R package survival. All *p*-values are adjusted for the number of tests within each type of analysis (gene or signature) using the Benjamini and Yekutieli False Discovery Rate (FDR) method to account for correlations amongst the tests.

The Kaplan-Meier curves are created using cut points in the observed gene expression or signature data, as appropriate. Graphs were constructed with GraphpadPrism 10.2.0. The Mantel-Haenszel test was used where appropriate.

#### Additional resource

• Tables S1–S3 and S5, S6 and Figures S1–S6.
• The Study protocol (NCT02400567; DOI: https://doi.org/10.1093/annonc/mdy448).
• The SUPPLEMENTAL Table S4, related to Figure S3 is deposited in the Mendeley repository. Changes to the BC360 signatures from paired Biopsy and Surgery samples, in each treatment arm. This table reports the signature name, variable (when applicable), time point comparison, Log(2) transformed fold change, 95% lower confidence of the mean limit (lcl), 95% upper confidence of the mean limit (ucl), Student’s t test distribution score, unadjusted significance (p-value), and significance adjusted for multiple tests (FDR)(padjust).

## Supplemental information

Document S1. Figures S1–S6 and Tables S1–S3, S5, and S6

Document S2. Article plus supplemental information
