# Supplementary material for: Molecular and cellular composition changes after neoadjuvant letrozole and palbociclib in early luminal breast cancer
Source: Cell Rep Med. 2026 Jan 9;7(1):102544. doi: 10.1016/j.xcrm.2025.102544 (PMC12866096; doi:10.1016/j.xcrm.2025.102544)

Cell Reports Medicine, Volume *7*

Supplemental information

Molecular and cellular composition changes after neoadjuvant letrozole and palbociclib in early luminal breast cancer

Paul Cottu, Yann Kieffer, Jerome Lemonnier, Véronique D'hondt, Francois P. Duhoux, Céline Callens, David Gentien, Cécile Reyes, Anais Boulai, Isabelle Desmoulins, Marie-Ange Mouret-Reynier, Christelle Levy, Pierre-Etienne Heudel, Florence Dalenc, Julien Grenier, Laetitia Fuhrmann, Sylvain Baulande, Suzette Delaloge, Fatima Mechta-Grigoriou, and Anne Vincent-Salomon

Figures S1-S6

Tables S1-S3 and S5-S6

NEOPAL Study Protocol

Figure S1.

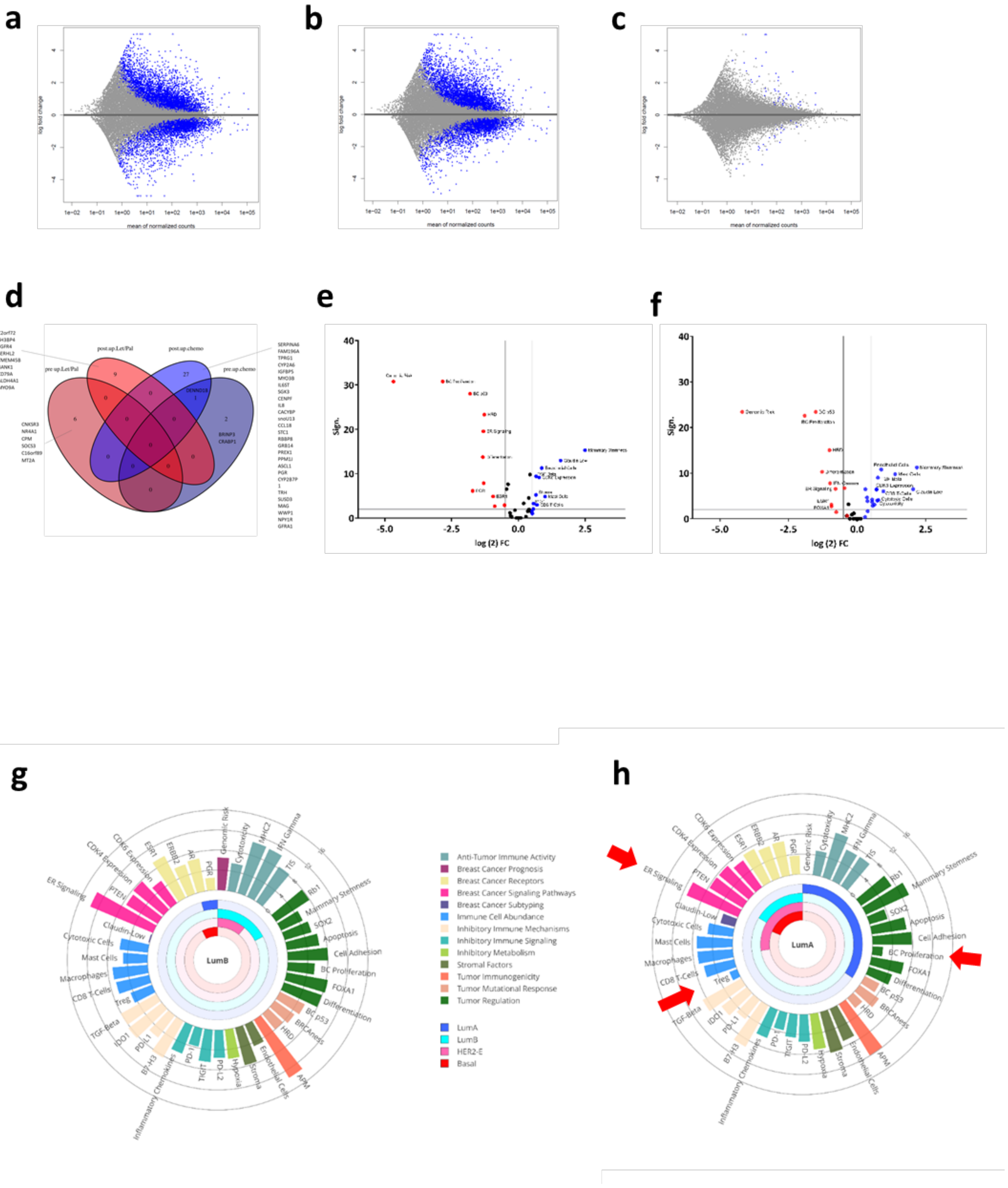

Figure S1. Differential gene expression and signature response in the letrozole+palbociclib and in the chemotherapy arms, related to Figure 3. *a*. Differential gene expression at baseline (pre) and after letrozole+palbociclib (post), derived from bulk RNA seq analysis. DESeq2 analyses adjusted p-values < 0.05. *b*. Differential gene expression at baseline (pre) and after chemotherapy (post), derived from bulk RNA seq analysis. DESeq2 analyses adjusted p-values < 0.05. *c*. Differential gene expression analysis between arms at

surgery. DESeq2 analyses adjusted p-values < 0.05. *d*. Venn diagram showing the genes that are differentially expressed in each treatment arm, before and after neoadjuvant therapy.e. Changes in signature scores by BC360 analysis in the letrozole+palbociclib arm. The x-axis indicates the log 2 fold change and the y-axis indicates the -LOG10 p-value. The vertical thin light grey lines indicate the -1 / +1 fold change and the horizontal thin light grey line indicates the 0.05 p-value threshold. f. Changes in signature scores by BC360 analysis in the chemotherapy arm. The x-axis indicates the log 2 fold change and the y-axis indicates the -LOG10 p-value. The vertical thin light grey lines indicate the -1 / +1 fold change and the horizontal thin light grey line indicates the 0.05 p value threshold. f. Example of a single patient analysis with the BC360 nCounter. The Wheel Plot shows the relative expression of each signature for a single sample. Signatures are grouped according to the biological process to which they belong. The correlation scores for Lum A, Lum B, HER2-E and Basal subtype are shown as a radial arc. Signature scores are shown as radial projections, with negative scores highlighted by a grey outline: Wheel plot analysis of baseline tissue from one patient, showing a dominant luminal B profile, partially HER2 enriched. f. Wheel plot analysis of surgical specimen from the same patient after 4 months of letrozole+palbociclib. The tumour has shifted to the luminal A subtype, with negative correlations with the other PAM50-defined intrinsic subtypes. Notably, ER signalling remains highly active, while the BC proliferation signature and genomic risk are almost extinguished.

Figure S2

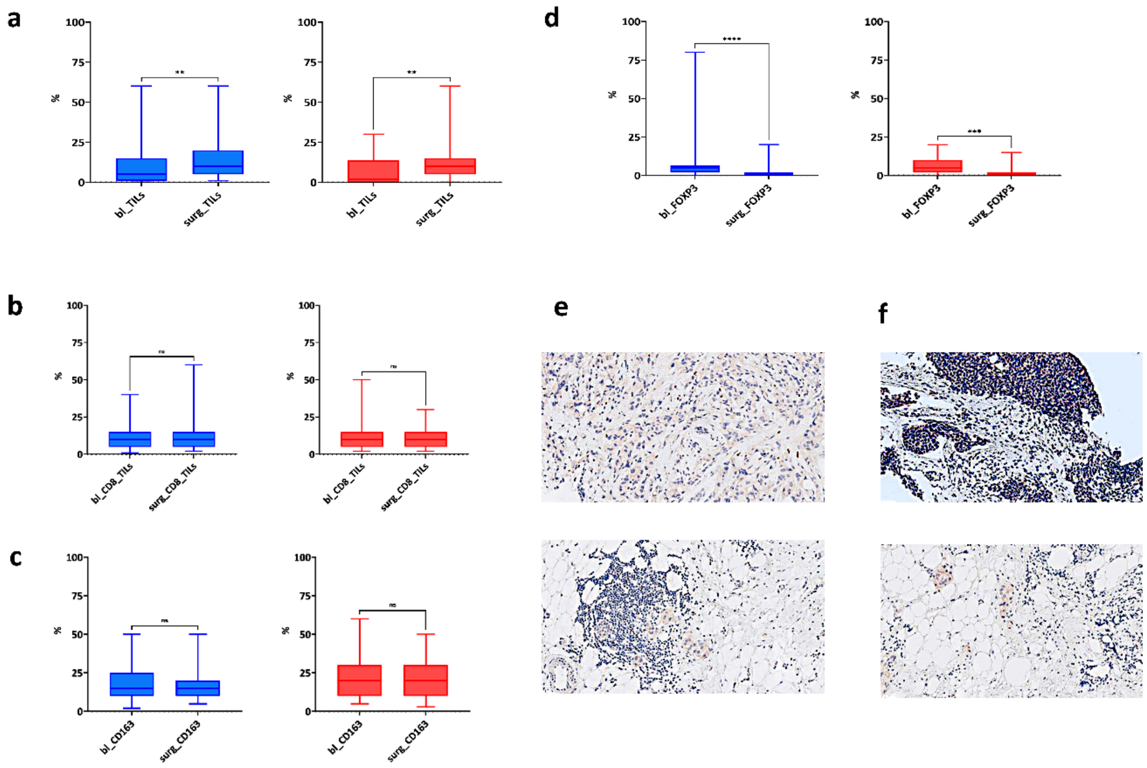

Figure S2. Immunohistochemical analysis of immune cell populations before and after treatment in the letrozole+palbociclib and in the chemotherapy arms, related to Figure 4. The boxplots show the variations in cellular staining, before and after treatment. In blue, patients in the letrozole-palbociclib; in red, patients in the chemotherapy arm. *a*. TILs count. *b*. CD8+ TILs count. *c*. CD163+ cells count. *d*. FOXP3+ cells count. *e*. Example of FOPX3+ cells decrease in the LP arm (top: baseline; bottom: at surgery). *f.* Example of FOPX3+ cells decrease in the CT arm (top: baseline; bottom: at surgery). *bl: baseline; surg.: surgery; TILs: tumour infiltrating lymphocytes, as per standard coloration and eyeballing; CD8_TILs: TILs count after CD8 staining; FOXP3: T regulators staining. P value are presented as asterisk (* means p<0.05; ** means p<0.01; *** means p<0.001; **** means p<0.0001; ns: non-significant).*

Figure S3

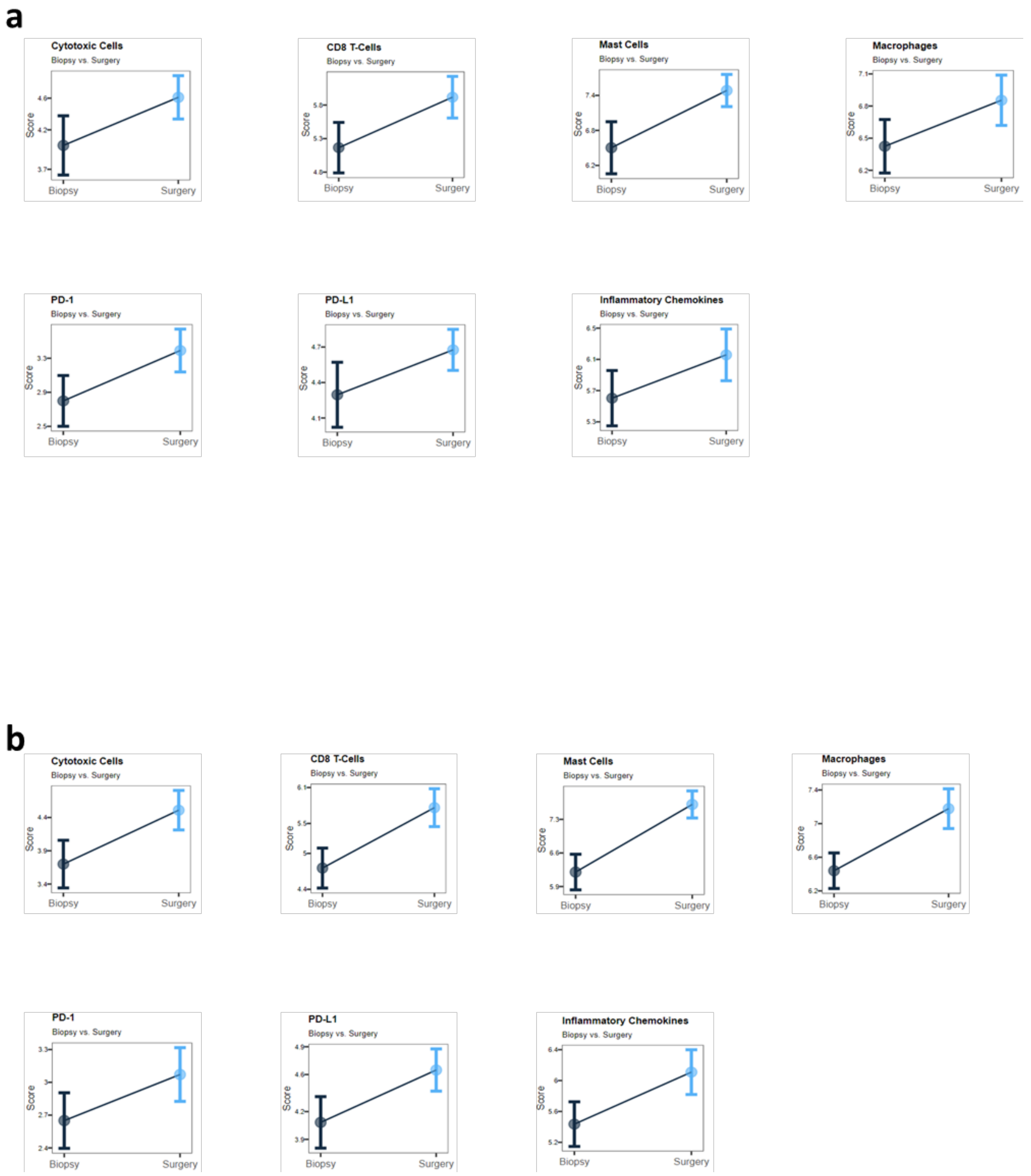

Figure S3. **Changes in immune-related BC360 signatures, related to Figure 4.** *a*. Letrozole-palbociclib arm. Plots show changes in immune-related BC360 signatures from paired biopsy and surgery samples in the letrozole+palbociclib arm. All p-values<.001. *b*. Chemotherapy arm**.** Plots show changes in immune-related BC360 signatures from paired biopsy and surgery samples in the chemotherapy arm. All p-values<.001.

Figure S4

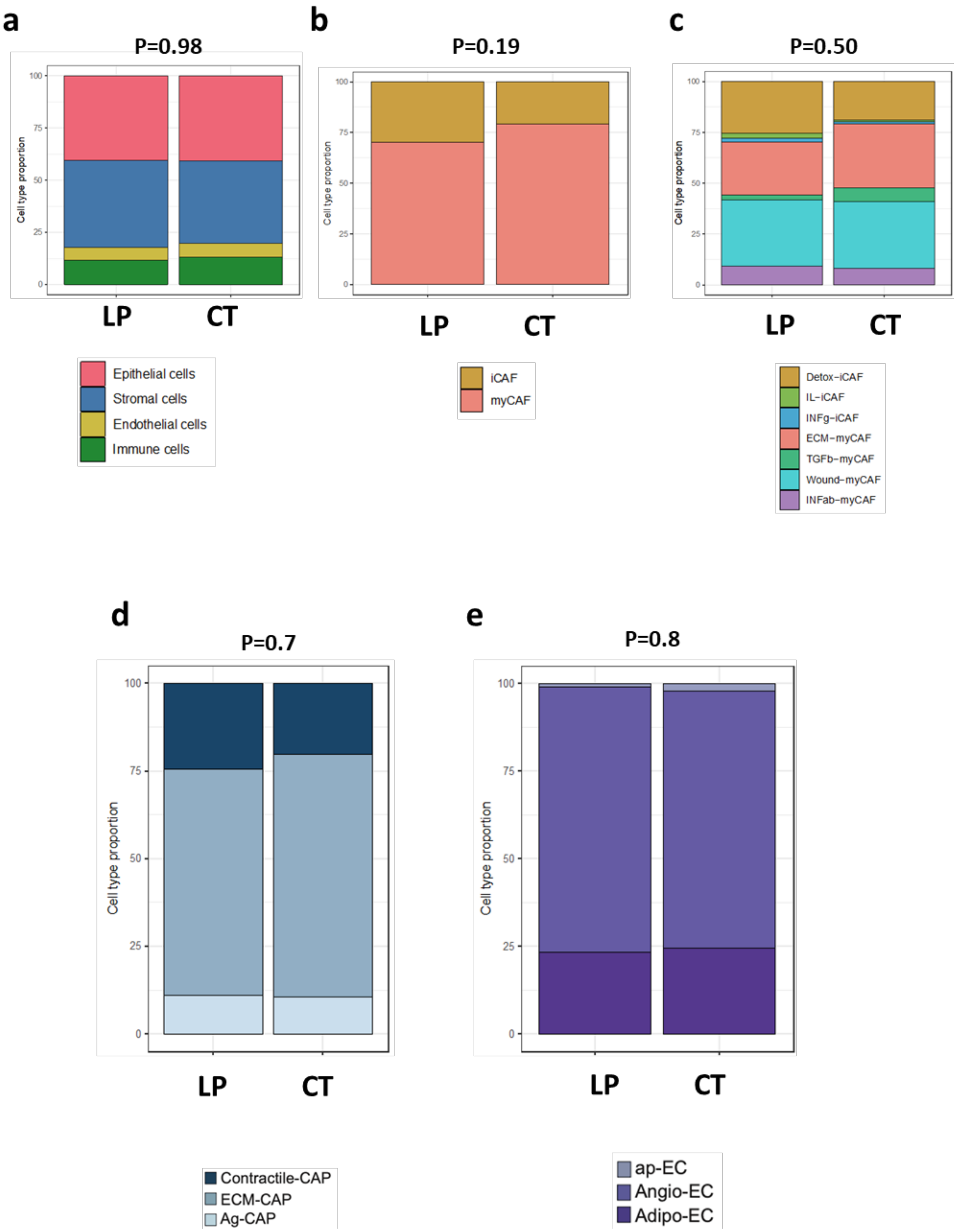

Figure S4. Comparison of the stromal changes in non-immune cells before and after treatment between the letrozole+palbociclib and the chemotherapy arms, related to Figure 6. Bar graphs showing for the letrozole-palbociclib (LP) and chemotherapy (CT) arms the cell types proportions after treatment. *a*. main cell

types proportions. *b*. iCAF and myCAF proportions. *c*. details of CAF subsets proportions. *d*. cancer-associated perivascular like fibroblasts proportions. *e*. endothelial cells proportions. Exploratory p-values are indicated above each graph.

iCAF: inflammatory CAF; myCAF: myofibroblastic CAF; Detox-iCAF: detoxification-associated iCAF; IL-iCAF: interleukine producing iCAF; INFg-iCAF; interferon gamma secreting iCAF; ECM-myCAF: extracellular matrix (ECM)-producing myCAF; TGFb-myCAF; tumor growth factor beta producing myCAF; INFab-myCAF: interferon alpha/beta producing myCAF; contractile-CAP: contractile cancer-associated perivascular like fibroblasts; ECM-CAP: ECM producing CAP; Ag-CAP: antigen CAP; ap-EC: antigen presenting endothelial cells; angio-EC: angiogenesis EC; adipo-EC: adipogenesis-related EC.

Figure S5

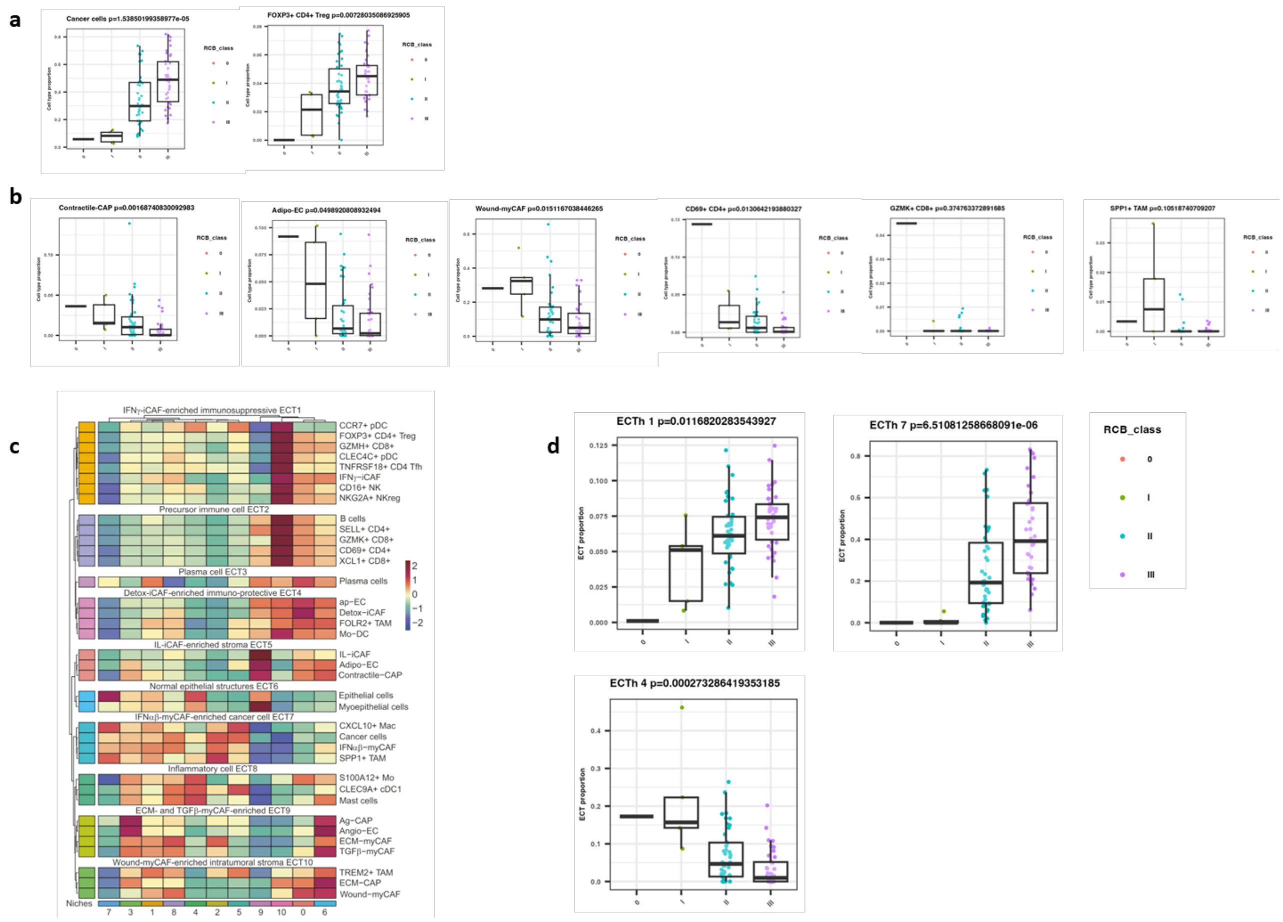

Figure S5. Correlations of RCB with cell composition at surgery (both arm combined), related to Figures 3, 4 and 6, and Table S5. *a*. cell types with positive correlation with RCB class (i.e: the persistence of a cell type is associated with a poor RCB response). *b*. cell types with a negative correlation with RCB class (i.e: the persistence of a cell type is associated with a favourable RCB response). *c*. identification of the EcoCellTypes that are present after neoadjuvant therapy. *d*. correlation of EcoCelltypes with RCB response. *In the box-and-whiskers plot, data are presented from min to max value, the middle bar is the mean, and the upper/lower bars or the boxes represent the 25%-75% interquartile range.*

Figure S6

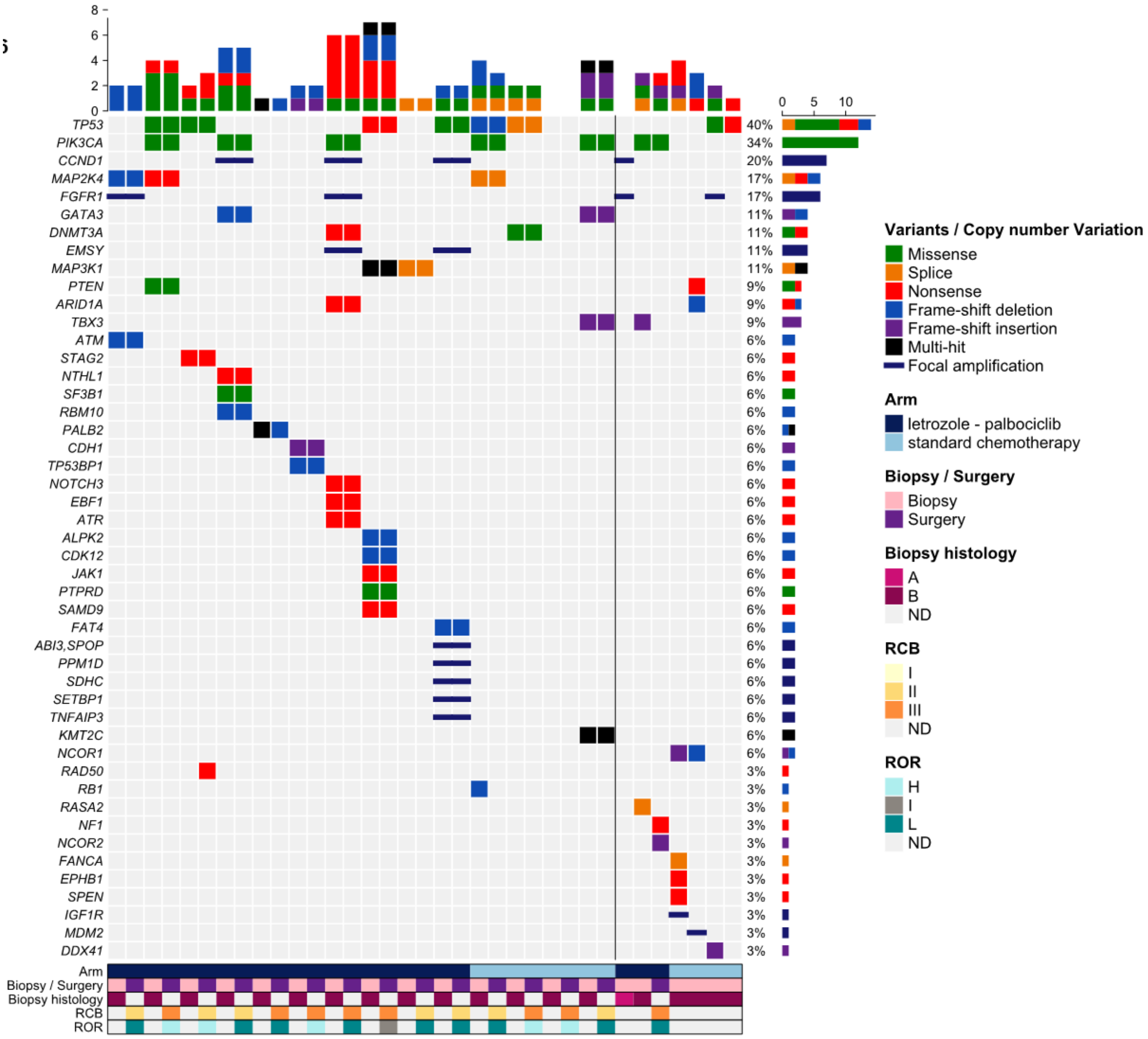

Figure S6. Mutational landscape of the NeoPAL trial cohort, related to Figure 3. Patients are sorted by treatment arm. Biopsy and matched tumor surgery samples are grouped on the left of the black vertical line. Single samples are on the right. *Biopsy histology: A, luminal A; B: luminal B. RCB: residual cancer burden; ROR: risk of recurrence.*

Table S1. Characteristics of main key biomarkers in the overall population and population subsets of the translational analysis, related to Figure 1.

	Overall population N=103	IHC subset N=91	BC360 subset N=88	RNAseq subset N=86	DNAseq subset N=21
PAM50 Luminal B subtype (%)	88	95.6	88.8	95.8	95
Baseline genomic risk (median, range)	70 (22-93)	71 (6-93)	69 (13-80)	71 (6-91)	77 (52-91)
RCB class 0-I (%)	11.6	7.7	10.2	8.2	0

Table S2. Differentially expressed genes after neoadjuvant therapy between the two arms, related to Figure S2.

Upregulated genes in the LP arm	Upregulated genes in the CT arm
C2orf72	SERPINA6
SH3BP4	FAM196A
FGFR4	TPRG1
SERHL2	CYP2A6
TMEM45B	IGFBP5
BANK1	MYO3B
CD79A	IL6ST
ALDH4A1	SGK3
MYO9A	CENPF
	IL8
	CACYBP
	snoU13
	CCL18
	STC1
	RBBP8
	GRB14
	PREX1
	PPM1J
	ASCL1
	PGR
	CYP2B7P1
	TRH
	SUSD3
	MAG
	WWP1
	NPY1R
	GFRA1
	DENND1B

LP: letrozole + palbociclib; CT: chemotherapy

Table S3. Details of the changes in cellular composition before and after neoadjuvant therapy, in each treatment arm, related to Figures 3, 4, 6 and S3

LP : biopsy vs surgical specimen		Chemotherapy : biopsy vs surgical specimen	
**Cell type**	**p-value**	**Cell type**	**p-value**
**Epithelial cells**	0.000202522	**Epithelial cells**	0.021977188
**Cancer cells**	1.57e-07	**Cancer cells**	1.52e-06
**CD4+ cells**			
SELL+ CD4+	8.60e-07	SELL+ CD4+	6.89e-09
CD69+ CD4+	5.77e-10	CD69+ CD4+	2.15e-13
TNFRSF18+ CD4 Tfh	0.001325589	TNFRSF18+ CD4 Tfh	2.85e-06
FOXP3+ CD4+ Treg	8.40e-13	FOXP3+ CD4+ Treg	3.27e-15
**CD8+ cells**			
XCL1+ CD8+	0.488756681	XCL1+ CD8+	0.490345893
GZMK+ CD8+	0.333915921	GZMK+ CD8+	0.904602129
GZMH+ CD8+	0.000479328	GZMH+ CD8+	1.59e-05
**TAM**			
FOLR2+ TAM	0.004200844	FOLR2+ TAM	0.002040702
TREM2+ TAM	8.46e-08	TREM2+ TAM	0.000248891
SPP1+ TAM	0.191033885	SPP1+ TAM	0.888797321
**Other immune cells**			
B cells	0.000186739	B cells	0.005112768
Mast cells	4.17e-05	Mast cells	2.89e-10
NKG2A+ NKreg	1.01e-19	NKG2A+ NKreg	5.31e-16
CD16+ NK	0.001805244	CD16+ NK	6.58e-07
**CAFs**			
Universal fibroblasts	6.86e-11	Universal fibroblasts	3.91e-18
Detox-iCAF	2.55e-07	Detox-iCAF	1.67e-05
IL-iCAF	0.000128848	IL-iCAF	0.000482667
INFg-iCAF	0.042898438	INFg-iCAF	0.326794431
ECM-myCAF	0.717059627	ECM-myCAF	0.818302071
TGFb-myCAF	0.018593367	TGFb-myCAF	0.002040702
Wound-myCAF	3.76e-16	Wound-myCAF	8.61e-13

INFab-myCAF	0.001805244	INFab-myCAF	0.000940665
**CAP**			
Contractile-CAP	2.87e-07	Contractile-CAP	1.83e-09
ECM-CAP	1.77e-08	ECM-CAP	8.61e-13
Ag-CAP	2.53e-12	Ag-CAP	1.48e-11
**EC**			
ap-EC	0.000866318	ap-EC	4.48e-05
Angio-EC	0.008873131	Angio-EC	0.012505306
Adipo-EC	1.54e-08	Adipo-EC	5.14e-07
**Others**			
Myoepithelial cells	4.19e-08	Myoepithelial cells	8.64e-09
Fibroblasts	0.015449905	Fibroblasts	0.157440304
S100A12+ Mo	2.30e-05	S100A12+ Mo	2.03e-10
Plasma cells	0.009944249	Plasma cells	0.001015042
CLEC4C+ pDC	0.04491657	CLEC4C+ pDC	0.002896161
CCR7+ pDC	0.24395962	CCR7+ pDC	0.000905377
CXCL10+ Mac	0.29742639	CXCL10+ Mac	0.880910874
CLEC9A+ cDC1	0.373140069	CLEC9A+ cDC1	0.928377609
Mo-DC	0.512757533	Mo-DC	0.00279801

In red: Down after treatment

In blue: up after treatment

LP : letrozole + palbociclib ; CT : chemotherapy ; CD4: CD4+ T cells; CD8: CD8+ T cells; TAM: tumour associated macrophage; SELL: Selectin L; TNFRSF: tumour necrosis factor super family receptor; Tfh: follicular helper T cell; XCL1: lymphotactin; GZM: granzyme; FOLR: folate receptor; TREM: triggering receptor expressed on myeloid cells; SPP: secreted phosphoprotein; NK: natural killer; NKG2A: killer cell lectin-like receptor G2A; iCAF: inflammatory CAF; myCAF: myofibroblastic CAF; Detox-iCAF: detoxification-associated iCAF; IL-iCAF: interleukine producing iCAF; INFg-iCAF; interferon gamma secreting iCAF; ECM-myCAF: extracellular matrix (ECM)-producing myCAF; TGFb-myCAF; tumor growth factor beta producing myCAF; INFab-myCAF: interferon alpha/beta producing myCAF; contractile-CAP: contractile cancer-associated perivascular like fibroblasts; ECM-CAP: ECM producing CAP; Ag-CAP: antigen CAP; ap-EC: antigen presenting endothelial cells; angio-EC: angiogenesis EC; adipo-EC: adipogenesis-related EC.

Table S5. Pearson correlation between cell type and RCB response after neoadjuvant treatment (both arms combined), related to Figure S5

Positive correlation (poor response)			Negative correlation (good response)		
**Cell type**	**r**	**p-value**	**Cell type**	**r**	**p-value**
Cancer cells	0.425	**0**	CD69+ CD4+	-0.421	**0**
FOXP3+ CD4+ Treg	0.275	**0.01**	GZMK+ CD8+	-0.394	**0**
Ag-CAP	0.21	0.052	SPP1+ TAM	-0.308	**0.004**
IL-iCAF	0.185	0.089	Contractile-CAP	-0.298	**0.005**
INFab-myCAF	0.165	0.128	Adipo-EC	-0.296	**0.006**
CXCL10+ Mac	0.158	0.146	Mo-DC	-0.27	**0.012**
GZMH+ CD8+	0.117	0.281	Wound-myCAF	-0.268	**0.013**
CD16+ NK	0.083	0.446	TNFRSF18+ CD4 Tfh	-0.231	**0.032**
TREM2+ TAM	0.082	0.452	Universal fibroblasts	-0.222	**0.04**
Epithelial cells	0.077	0.478	FOLR2+ TAM	-0.205	0.059
TGFb-myCAF	0.038	0.731	Detox-iCAF	-0.196	0.071
CCR7+ pDC	0.023	0.833	Fibroblasts	-0.194	0.073
ECM-myCAF	0.014	0.897	Myoepithelial cells	-0.189	0.082
			CLEC9A+ cDC1	-0.184	0.09
			S100A12+ Mo	-0.181	0.095
			B cells	-0.167	0.124
			CLEC4C+ pDC	-0.164	0.13
			Plasma cells	-0.161	0.139
			ECM-CAP	-0.154	0.156
			Angio-EC	-0.098	0.369
			INFg-iCAF	-0.094	0.387
			NKG2A+ NKreg	-0.093	0.395
			ap-EC	-0.072	0.508
			Mast cells	-0.055	0.617
			XCL1+ CD8+	-0.033	0.764
			SELL+ CD4+	-0.024	0.824

Table S6. Correlation between cell type and ROR response after neoadjuvant treatment (Wilcoxon test), related to Figure S5. Cell types associated with a decrease in ROR score (i.e. good response) are highlighted in green, whereas cell types associated with the persistence of a high ROR score (poor response) are shown in red.

	2 arms combined		Letrozole + Palbociclib		Chemotherapy	
**cell.type**	**p-value**	**comments**	**p-value**	**comments**	**p-value**	**comments**
Myoepithelial cells	4.65E-06	Up ROR low	0.056173433		3.44E-05	Up ROR low
Universal fibroblasts	0.000421613	Up ROR low	0.001736559	Up ROR low	0.112415804	
Contractile-CAP	0.000438562	Up ROR low	0.004654792	Up ROR low	0.039133569	Up ROR low
Ag-CAP	0.001089359	Up ROR high	0.172295127		0.002494404	Up ROR high
FOXP3+ CD4+ Treg	0.002208959	Up ROR high	0.191837481		0.014925109	Up ROR high
NKG2A+ NKreg	0.003527919	Up ROR low	0.18187747		0.034055126	Up ROR low
ECM-CAP	0.007271097	Up ROR low	0.01340209	Up ROR low	0.118816148	
B cells	0.007957601	Up ROR low	0.610224099		0.009093767	Up ROR low
Cancer cells	0.008198849	Up ROR high	0.439435765		0.009899683	Up ROR high
CD69+ CD4+	0.010990163	Up ROR low	0.115252284		0.112415804	
CD16+ NK	0.014993035	Up ROR high	0.064547112		0.125491593	
Adipo-EC	0.016735379	Up ROR low	0.042054255	Up ROR low	0.155069232	
Detox-iCAF	0.021877763	Up ROR low	0.014647182	Up ROR low	0.527073214	
Epithelial cells	0.033741943	Up ROR low	0.405483232		0.198937454	
Wound-myCAF	0.097196691		0.94201717		0.054619183	
ap-EC	0.099195657		0.782176069		0.044823565	Up ROR low
CLEC4C+ pDC	0.118676568		0.872856192		0.018886613	Up ROR low
Plasma cells	0.143662902		1		0.089434072	
Mo-DC	0.160459872		0.91889085		0.008345492	Up ROR low
S100A12+ Mo	0.181861124		0.313188094		0.047914044	Up ROR low
Angio-EC	0.188343746		0.474917664		0.32486437	
Mast cells	0.227023202		0.202180975		0.442721039	
CCR7+ pDC	0.234617753		0.782176069		0.031729495	Up ROR low
TREM2+ TAM	0.262591693		0.672875403		0.527073214	
SELL+ CD4+	0.266766392		0.22403763		0.676819619	
TNFRSF18+ CD4 Tfh	0.306366377		0.71593922		0.338393817	

GZMH+ CD8+	0.329950057		0.247482085		0.716629374	
CLEC9A+ cDC1	0.494756391		0.827236259		0.125491593	
FOLR2+ TAM	0.500908111		0.493215015		0.084309744	
GZMK+ CD8+	0.51960093		0.782176069		0.311683886	
INFab-myCAF	0.51960093		0.759899691		0.338393817	
IL-iCAF	0.673723255		0.849985099		0.637873243	
TGFb-myCAF	0.687924804		0.357516348		0.696622422	
Fibroblasts	0.72389839		0.694287547		0.637873243	
ECM-myCAF	0.812591719		0.91889085		0.798447751	
CXCL10+ Mac	0.835191495		0.872856192		0.757206202	
SPP1+ TAM	0.873149513		0.91889085		0.637873243	
XCL1+ CD8+	0.926760026		0.849985099		0.527073214	
INFg-iCAF	0.972984393		0.759899691		0.492400762	

Supplementary Appendix (online-only)

Study protocol

French Breast Cancer Intergroup-UNICANCER (UCBG)

EudraCT N° 2014-002560-33

CARMINA04 - UC-0140/1404

Open-label, randomized, multicenter, international, parallel exploratory phase II study, comparing 3 FEC-3 Docetaxel chemotherapy to letrozole + palbociclib combination as neoadjuvant treatment of stage II-IIIA PAM 50 defined Luminal breast cancer, in postmenopausal women

Abbreviated title: NEOPAL

Version 3. 2 - February 16th, 2016

	CPP Approval	ANSM Approval	VERSION
INITIAL PROTOCOL	12/11/2014	12/12/2014	v1.2 - Dec 03rd, 2014
AMENDEMENT 1	04/02/2015	26/02/2015	v2.0 – Dec 22nd, 2014
AMENDEMENT 2	29/04/2015	28/05/2015	v2.1 – March 27th, 2015
AMENDEMENT 3	15/12/2015	20/11/2015	v3.1 - Aug 06th, 2015
AMENDEMENT 4	05/04/2016	08/07/2016	v3.2 - Aug 16th, 2016

**INTERNATIONAL COORDINATORS**	**Paul COTTU, MD, MSc**<br>Oncologist, Department of Medical Oncology,<br>Institut CURIE, 26, rue d'ULM, 75005 Paris, FRANCE<br>Phone : +33 (0)1 44 32 46 81; Fax : +33 (0)1 53 10 40 26<br>e-mail : paul.cottu@curie.fr<br><br>Suzette DELALOGE, MD, MSc<br>Oncologist, Breast Pathology department<br>Gustave Roussy, 114 rue Edouard Vaillant, 94805 Villejuif, FRANCE<br>Tél : +33 (0)1 42 11 42 11; Fax : +33 (0)1 42 11 52 74<br>e-mail : suzette.delaloge@gustaveroussy.fr

**SPONSOR**	**UNICANCER**<br>101, rue de Tolbiac - 75654 PARIS CEDEX 13 - FRANCE<br>*Tel. +33.(0)1.44.23.04.04 - Fax: +33.(0)1.44.23.04.69*

APPROVALS AND CONTACT DETAILS

PROTOCOL NeoPAL

Open-label, randomized, multicenter, international parallel exploratory phase II study, comparing 3 FEC-3 Docetaxel chemotherapy to letrozole + palbociclib combination as neoadjuvant treatment of stage II-IIIA PAM 50 defined Luminal breast cancer, in postmenopausal women

**FRENCH COMPETENT AUTHORITY**	**Agence Nationale de la Sécurité du Médicament et des produits de santé (ANSM)**	**Date of initial authorization: December 2014, 12th**
		**Ref. number: 141044A-12**
**FRENCH ETHIC COMMITTEE**	**CPP Ile de France III**	**Date of initial approval: November 2014, 12th**
		**Ref. number : 3186**

WRITING COMMITTEE	Bernard ASSELAIN, Ivan BIECHE, Paul COTTU, Suzette DELALOGE, David GENTIEN, Jérome LEMONNIER, Anne VINCENT-SALOMON

NAME AND RESPONSIBILITIES	ADDRESS	E-MAIL
**Dr Paul COTTU**<br>**International Coordinator**	Institut Curie<br>Medical Oncology department<br>26 rue Ulm<br>Paris, 75005<br>Tel : +33 (0)1 44 32 46 81<br>Fax : +33 (0)1 53 10 40 26	paul.cottu@curie.fr
**Dr Suzette DELALOGE**<br>**International Coordinator**	Gustave Roussy<br>Breast Pathology department<br>114 rue Edouard Vaillant<br>94805 Villejuif<br>Tel : +33 (0)1 42 11 42 11<br>Fax : +33 (0)1 42 11 52 74	suzette.delaloge@gustaveroussy.fr
**Jérôme LEMONNIER**<br>**Project manager**	R&D UNICANCER<br>101, rue de Tolbiac<br>75654 PARIS Cedex 13<br>Tel. : +33 (0)1.71.93.67.02<br>Fax : +33 (0)1.44.23.04.69	j-lemonnier@unicancer.fr
**Lisa Belin**<br>**Statistician**	Institut Curie<br>Biostatistic department<br>26 rue Ulm<br>Paris, 75005<br>Tel : +33 (0)1 44.32.46.66<br>Fax : +33 (0)1.43.29.02.03	lisa.belin@curie.fr

SYNOPSIS – PROTOCOL UC-0140/1404

A) CLINICAL TRIAL IDENTIFICATION	
**SPONSOR - PROTOCOL CODE NUMBER** : CARMINA04 - UC-0140/1404<br>**VERSION AND DATE:** V 3.2 OF FEB 16TH, 2016	
**TRIAL TITLE :** Open-label, randomized, multicenter, international, parallel exploratory phase II study, comparing 3 FEC-3 Docetaxel chemotherapy to letrozole + palbociclib combination as neoadjuvant treatment of stage II-IIIA PAM defined Luminal breast cancer, in postmenopausal women.	
**ABBREVIATED TITLE : NEOPAL**	
**PRINCIPAL INVESTIGATORS**	
**Paul COTTU, MD, MSc**<br>Department of Medical Oncology<br>Institut CURIE<br>26 rue d'ULM<br>75005 Paris<br>Phone : +33 (0)1 44 32 46 81<br>Fax : +33 (0)1 53 10 40 26<br>E-mail : paul.cottu@curie.net	**Suzette DELALOGE, MD, MSc**<br>Department of Medical Oncology<br>Gustave ROUSSY<br>114 r Edouard Vaillant<br>94805 VILLEJUIF CEDEX<br>Phone : +33 (0)1 42 11 42 11<br>Fax : +33 (0)1 42 11 52 74<br>E-mail : suzette.delaloge@gustaveroussy.fr
**Planned number of investigational sites: 40**	**number of randomized subjects : 132**<br>**(~180 patients needed to be screened)**

B) SPONSOR IDENTIFICATION	
**NAME OF THE INSTITUTION: UNICANCER**	
**CONTACT PERSON:**	Jérôme LEMONNIER, R&D UNICANCER<br>101 rue de Tolbiac - 75654 PARIS Cedex 13<br>Tél. : +33 (0)1.71.93.67.02 - Fax : +33 (0)1.44.23.04.69<br>E-mail : j-lemonnier@unicancer.fr

C) TRIAL GENERAL INFORMATION

MEDICAL CONDITION : Post-menopausal patients with localized, stage II-IIIA, candidate for chemotherapy but not candidate or uncertain for breast conservation, PAM50 (Prosigna™)- defined Luminal A and Node-positive or Luminal B (ER+ and HER2-) operable breast cancer (randomized cohort), or Luminal A N- (non randomized cohort).

METHODOLOGY : Open-label, randomized, multicenter, parallel exploratory phase II study, comparing sequential standard chemotherapy (3 FEC 100-3 Docetaxel 100) and a same duration letrozole + palbociclib combination as neoadjuvant treatment of stage II-IIIA PAM 50 defined Luminal A-Node+/Luminal B breast parallel single arm cohort of Luminal A N- will be treated with the same duration letrozole + palbociclib combination.

MAIN OBJECTIVE :

To evaluate the ability of each treatment strategy to provide RCB 0-I pathological tumor response at surgery (local assessment) in luminal A N+ and luminal B patients subgroup.

SECONDARY OBJECTIVES:

1- EFFICACY:

- To evaluate clinical responses in each arm and in each subgroups (luminal A N+ and luminal B; luminal A N-), as defined by clinical and US examination;
- To evaluate the rates of breast conservation therapy according to each treatment scheme;
- To evaluate the progression free survival (PFS) and invasive disease free survival (iDFS) at 3 years follow-up;
- To evaluate RCB 0-1 pathological tumor response by central pathology reading

2- TOXICITY

- To assess the safety of each treatment strategy.

3- BIOLOGY

- To assess the positive predictive value of ROR on clinical and pathological tumor response in both treatment arms;
- To evaluate the ability of predefined biomarkers to predict for clinical and pathological response in each arm, such as p53 mutation status, RB and CCND1 status, as well as other proliferation and senescence biomarkers.

C) TRIAL GENERAL INFORMATION

INCLUSION CRITERIA :

1) Aged ≥ 18 years, post-menopausal women according to the following criteria:

    Age > 60 years,

    Bilateral ovariectomy,

    Age ≤ 60, with an uterus and presenting an amenorrhea of more than 12 months,

    Age ≤ 60 without an uterus and FSH > 20 IU/L.

2) Newly diagnosed and operable unilateral invasive breast cancer, not candidate or uncertain for breast conservation

    Note: Multicentric/multifocal tumors are allowed provided a maximum of 3 lesions are present, and all share the same characteristics: ER Allred 4, Her2- (PAM50 will be performed in the largest lesion).

3) Stage II-IIIA
4) Assessment of nodal status available (Ultrasound guided FNA or biopsy if necessary)
5) Non metastatic, M0
6) ER-positive by IHC (Allred Score≥4)
7) HER2-negative by IHC (score 0 or 1+) and/or FISH/CISH
8) PAM50 subtyping (Prosigna™) centralized evaluation available and:
    - Either Luminal A AND proven nodal involvement (cytology or histology), or Luminal B for randomized patients
    - Or Luminal A N- for non-randomized patients
9) ECOG 0-1
10) No prior systemic therapy for the present tumor
11) Adequate renal, hepatic, and hematopoietic functions as defined by the following criteria:
    - Absolute Neutrophil Count (ANC) ≥1,500/mm^3 or ≥1.5 x 10^9/L
    - Platelets ≥100,000/mm3 or ≥100 x 10^9/L
    - Hemoglobin ≥9 g/dL
    - Serum Aspartate Transaminase (AST) and serum Alanine Aminotransferase Transaminase (ALT) ≤2.5 x upper limit of normal (ULN)
    - Alkaline phosphatase ≤2.5 x ULN
    - Total serum bilirubin ≤1 x ULN
    - Serum creatinine ≤1.5 x ULN or estimated creatinine clearance ≥ 60 mL/min as calculated using the method standard for the institution
12) Adequate cardiac functions, including:
    - 12 Lead electrocardiogram (ECG) with normal tracing or non clinically significant changes that do not require medical intervention.
    - QTc interval ≤480 msec
    - No history of Torsades de Pointes or other symptomatic QTc abnormality.
13) Willingness and ability to comply with scheduled visits, treatment plan, laboratory tests, and other trial procedures
14) Signed informed consent and health insurance coverage

NON INCLUSION CRITERIA :

1) Non operable, bilateral, T4 or metastatic breast cancer
2) Limited T2 breast cancer immediately accessible to conservative surgery
3) Previous homolateral breast cancer (including in situ carcinoma), and/or contralateral breast cancer except if treated by surgery +/- radiation therapy alone without any systemic treatment
4) Previous hormone replacement therapy (HRT) stopped less than 2 weeks before beginning of treatment
5) Previous use of SERMs such as raloxifene
6) Any surgery (not including minor procedures such as lymph node biopsy, primary tumor core biopsy, fine needle aspiration) within 4 weeks of start of study treatment; or not fully recovered from any side effects of previous procedures.
7) Diagnosis of any previous malignancy within the last 5 years, except for adequately treated basal cell carcinoma, or squamous cell skin carcinoma, or in situ cervical carcinoma
8) Diagnosis of hypokalemia
9) History of any previous anti-cancer chemotherapy and any previous treatment using AI
10) Concurrent administration of herbal preparations as complementary medicine.
11) Any clinically significant gastrointestinal abnormalities, which may impair intake, transit or absorption of the study drugs, such as the inability to take oral medication in tablet form and malabsorption syndrome
12) Patient with any psychological, familial, social or geographical condition which could potentially hamper compliance with the study protocol and follow-up schedule; those conditions should be discussed with the patient before registration in the trial.

EVALUATION CRITERIA :

PRIMARY EFFICACY ENDPOINT :

- Local RCB rates in the two arms of the study in Luminal A N+ and Luminal B patients

SECONDARY ENDPOINTS :

<u>In Luminal A N+ and Luminal B patients:</u>

- Clinical/radiological response rates in each treatment arm (RECIST 1.1)
- Safety (CTC-AE V4.0)
- Relative dose intensity of each drug in both arms
- Positive and negative predictive values of PAM50 ROR-defined status in both arms
- Assessment of several biomarkers as potential predictors of clinical and pathological response in both arms
- Central RCB rates
- Rates of BCS in the two arms, with regard to the initially planned surgery

In Luminal A N- patients:

- Clinical/radiological response rates (RECIST 1.1)
- Safety (CTC-AE V4.0)
- Central RCB rates
- Rates of BCS with regard to the initially planned surgery

D) DESCRIPTION OF INVESTIGATIONAL MEDICINAL PRODUCTS				
**Drug Name (DCI)**	**Commercial Name**	**Pharmaceutical Form**	**Administration Route**	**Posology**
Palbociblib	-	Capsules (125 mg, 100mg, 75mg)	PO	125mg/day (3weeks/4)
Letrozole	Femara®	Tablets	PO	2.5 mg/day
5 Fluoro-uracile	5 Fluoro-uracile	Injectable	IV	500 mg/m² / course
Epirubicine	Epirubicine	Injectable	IV	100 mg/m² / course
Cyclophosphamide	Endoxan	Injectable	IV	500 mg/m² / course
Docetaxel	Taxotere	Injectable	IV	100 mg/m² / course

STUDY TREATMENT AND SCHEDULE

Overall duration of treatment will be 19 weeks, meaning the same duration of treatment in all patients

Luminal A N- patients will not be randomized, they will all receive:

- Palbociclib at the daily dose of 125 mg on a discontinuous 3 weeks/4 schedule up to the day prior to surgery
- Letrozole at the dose of 2.5 mg/day up to the day prior to surgery

Luminal A N+ and Luminal B breast cancer patients will be randomized between two arms:

Arm A :

- Palbociclib will be administered at the daily dose of 125 mg on a discontinuous 3 weeks/4 schedule up to the day prior to surgery
- Letrozole will be administered at the dose of 2.5 mg/day up to the day prior to surgery

Arm B :

Chemotherapy: patients will be planned to receive 3 cycles of FEC100 (5 FU 500 mg/m², Epirubicin 100 mg/m², Cyclophosphamide 500 mg/m²) every 3 weeks, followed by 3 cycles of Docetaxel 100 mg/m² every 3 weeks up to 4 weeks prior to surgery

Post-surgery therapy will be administered as per local procedures. Chemotherapy is encouraged in Arm A RCB II-III patients, and participation into post neoadjuvant clinical trials is encouraged in Arm B RCB II-III patients.

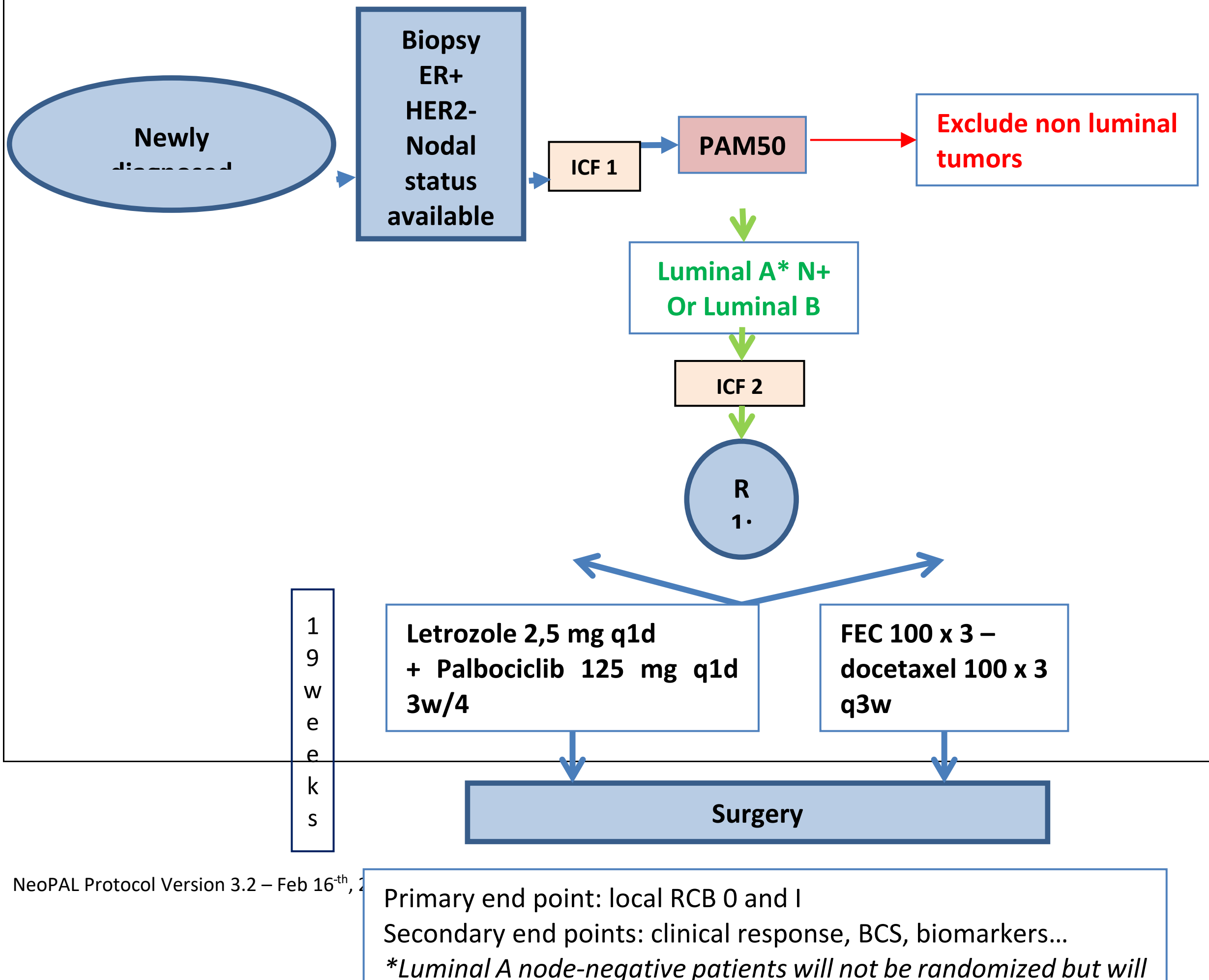

E) SAMPLE SIZE DETERMINATION and STATISTICAL CONSIDERATIONS

1) Sample size and decision rule

A Fleming two-step statistical design will be used in the experimental arm:

- Based on the RCB 0-I results with standard chemotherapy as administered in the standard arm, the null hypothesis (p0) will be that RCB 0-I is observed in 20% of the cases (p0=0.20)
- The alternative hypothesis will be p1 = 40% (0.40)
- Accepting a type I error of 0.045, and a type II error of 0.042 (power = 95.8%), the required number of patients is 60 evaluable patients per arm, therefore 132 patients will need to be included (estimation of 10% risk of non-evaluable patients). As about 10% of PAM50 evaluable patients will be classified as non-luminal, and taking into account potential technical failures, about 180 patients will be screened.

- The first interim analysis will be planned after 30 patients (luminal A N-positive and luminal B) are available for local RCB evaluation in the experimental arm (step 1)

At the end of step 1, among the first 30 patients included in the experimental arm

- o If 5 or less than 5 local RCB 0-I are observed (16.7%), the trial will be stopped for futility
- o If 6 or more local RCB 0-I are observed, the trial will continue accrual

After inclusion of 60 evaluable patients in both arms, final analyses will be conducted (step 2):

- o If 17 or less than 17 local RCB 0-I are observed (28.3%) in the experimental arm, the trial's objective will be considered as not reached,
- o If 18 or more than 18 local RCB 0-I are observed (30%), a 20% RCB 0 – 1 rate can be rejected and the trial's objective will be considered as reached.

The observed local RCB's rate in the control group will be checked at step 1 and 2 in order to verify the a priori hypothesis (20% RCB 0-1 in the control arm)

As the safety of the experimental strategy has been evaluated in the metastatic setting, no interruption in the accrual will be done between step 1 and 2, in order to maintain the dynamic of accrual in the trial

Luminal A N-negative patients will be analyzed independently as a prospective cohort.

2) Randomizations will be equally balanced between the two arms and will be stratified based on :

- T2 versus T3
- PAM 50 luminal A vs luminal B

F) TRIAL DURATION
**INCLUSION PERIOD :** 2 YEARS
**TREATMENT DURATION :** 19 WEEKS
**FOLLOW-UP DURATION :** 3 YEARS
**OVERALL TRIAL DURATION :** 5,5 YEARS

TABLE OF CONTENT

ABBREVIATIONS 32
1. RATIONALE OF THE TRIAL 33
1.1. NEOADJUVANT SYSTEMIC THERAPY IN ER+ BREAST CANCER 33
1.2. PAM50 SIGNATURE IN LUMINAL BREAST CANCER 33
1.3. PALBOCICLIB IN LUMINAL BREAST CANCER 34
1.4. THE RESIDUAL CANCER BURDEN 35
1.5. SUMMARY OF RATIONALE 36
2. TRIAL'S OBJECTIVES 36
2.1. MAIN OBJECTIVE 36
2.2. SECONDARY OBJECTIVES 36
3. METHODOLOGY 37
4. PATIENT SELECTION 38
4.1. INCLUSION CRITERIA 38
4.2. NON-INCLUSION CRITERIA 39
5. RANDOMIZATION 40
6. TREATMENTS 40
6.1. PALBOCICLIB 40
6.1.1. PACKAGING, LABELING, STORAGE CONDITIONS 41
6.1.2. DISPATCH, DISTRIBUTION AND ACCOUNTING 41
6.1.3. PALBOCICLIB ADMINISTRATION 42
6.2. LETROZOLE 42
6.3. CHEMOTHERAPY 43
6.4. GENERAL RULES 43
6.5. COURSE OF THE TREATMENT 44
7. DOSE MODIFICATIONS AND MANAGEMENT OF SPECIFIC TOXICITIES 45
7.1. GENERAL CONSIDERATIONS 45
7.2. PALBOCICLIB 45
7.2.1. DOSING INTERRUPTIONS 46
7.2.2. DOSE DELAY 46
7.2.3. DOSE REDUCTIONS 47
7.3. LETROZOLE 49
7.4. CHEMOTHERAPY 49
8. CONCOMITANT TREATMENTS 51
8.1. AUTHORIZED CONCOMITANT TREATMENTS 51
8.2. PROHIBITED CONCOMITANT TREATMENTS 52
9. STUDY PROCEDURE 55
9.1. BASELINE ASSESSMENT 56
9.2. ASSESSMENTS DURING TREATMENT PHASE AT DAY 1 OF EACH CYCLE. 57
9.3. END OF TREATMENT EVALUATION 58

9.4. SURGERY 58
9.5. PATHOLOGY ASSESSMENT 59
9.6. FOLLOW-UP VISITS 59
10. PREMATURE END OF THE TREATMENT 60
11. TRIAL TERMINATION CRITERIA 60
12. EVALUATION CRITERIA 61
12.1. MAIN CRITERION 61
12.2. SECONDARY CRITERIA 62
12.2.1. EFFICACY 62
12.2.2. SAFETY 62
12.2.3. BIOLOGY 62
13. STATISTICS 62
13.1. SAMPLE SIZE DETERMINATION AND STATISTICAL ANALYSIS 62
13.2. DEFINITION OF POPULATION 63
14. PATHOLOGY 64
14.1. TASK FOR THE LOCAL PATHOLOGIST 64
14.1.1. TUMOR MATERIAL FOR CENTRAL PAM50 TESTING 64
14.1.2. RCB DETERMINATION 65
14.1.3. SURGERY TUMOR MATERIAL FOR CENTRAL REVIEW 66
14.1.4. LABELLING AND SHIPMENT OF TUMOR MATERIAL 66
14.2. TASK FOR THE CENTRAL PATHOLOGIST 66
14.2.1. PAM 50 TESTING 66
14.2.2. RNA AND DNA COLLECTION 67
15. SAFETY 67
15.1. ADVERSE EVENT 67
15.1.1. GENERAL DEFINITION 67
15.1.2. KNOWN UNDESIRABLE EFFECTS OF PALBOCICLIB 67
15.2. SERIOUS ADVERSE EVENT 68
15.2.1. GENERAL DEFINITION 68
15.2.2. DEFINITION OF SUSPECTED UNEXPECTED SERIOUS ADVERSE REACTION (SUSAR) 68
15.2.3. SEVERITY CRITERION 69
15.2.4. HANDLING OF A SERIOUS ADVERSE EVENT 69
16. STEERING COMMITTEE AND INDEPENDENT DATA MONITORING COMMITTEE 70
16.1. TRIAL STEERING COMMITTEE 70
16.2. INDEPENDENT DATA MONITORING COMMITTEE (IDMC) 70
17. QUALITY INSURANCE AND QUALITY CONTROL 71
18. DATA PROTECTION AND CONFIDENTIALITY MANAGEMENT 71
19. PUBLICATION GUIDELINES 72
19.1. PRIMARY MANUSCRIPT AND PRESENTATION 72
19.2. SECONDARY MANUSCRIPTS 72
19.3. SUB-STUDIES MANUSCRIPTS 72

20. TRANSLATIONAL RESEARCH 72
21. DATABASE ACCESS 73
22. ETHICAL AND REGULATORY ISSUES 73
22.1. GENERAL REQUIREMENTS 73
22.2. INDEPENDENT ETHICS COMMITTEE (IEC) OR INSTITUTIONAL REVIEW BOARD (IRB) 74
22.3. COMPETENT AUTHORITY 74
22.4. PATIENT INFORMATION AND THE INFORMED CONSENT OF PARTICIPANTS 74
22.5. SPONSOR RESPONSABILITIES 75
22.6. INVESTIGATOR RESPONSIBILITIES 75
22.7. FEDERATION OF THE PATIENT COMMITTEES FOR CLINICAL RESEARCH IN ONCOLOGY 76
23. BIBLIOGRAPHY 77

Appendix 1a – Investigation Summary Table – Arm A (Letrozole + Palbociclib) 79
Appendix 1b – Investigation Summary Table – Arm B (Chemotherapy: 3 FEC 100 + 3 Doc 100). 80
Appendix 2 – Treatment schedule 82
Appendix 3 – ECOG peformance status scale 83
Appendix 4 – Toxicity criteria (CTCAE) 84
Appendix 5 –Summary of Product Characteristics 85
Appendix 6: Detailed Pathology Methods for Using Residual Cancer Burden (RCB) 86
Appendix 7: Scheme of logistic flow for pathological samples 96

List of Tables

Table 1: PAM50 results in neoadjuvant studies 34
Table 2: PCR and RCB (%) according to ROR-S and Ki67 status in I-SPY1 trial 36
Table 3. Palbociclib Dose Levels 47
Table 4: Palbociclib Dose Modifications for Treatment Related Toxicities Requiring Treatment Interruption/Delay or Persisting Despite Optimal Medical Treatment. 48
Table 5: Management guidelines in case of QTc Prolongation 48
Table 6: Management guidelines in case of Hematological Toxicity 50
Table 7: Management guidelines in case of Non Hematological Toxicities (Except Alopecia) 51
Table 8: Docetaxel specific toxicities 51
Table 9: Clinically relevant drug interaction: substrates, inducers and inhibitors of isoenzyme CYP3A. 54

ABBREVIATIONS

AI: Aromatase Inhibitor

ALP: Alkaline Phosphatase

ALT: Alanine aminotransferase

AST: Aspartate aminotransferase

AUC: Area under curve

BC: Breast Cancer

CDK: Cyclin-Dependent Kinase

EFS: Event Free Survival

EGFR: Epidermal Growth Factor Receptor

ER: Estrogen Receptor

FAC: 5-fluorouracil, doxorubicin and cyclophosphamide

FEC: 5-fluorouracil, epirubicin and cyclophosphamide

FFPE: Formalin Fixed and Paraffin Embedded

FNA: Fine Needle Aspiration

HER2: Human Epidermal Growth Factor Receptor 2

HR: Hormonal Receptor

iDFS: invasive Disease Free Survival

IEC: Independent Ethic Committee

IGF: Insulin Growth Factor

IGFR: Insulin Growth Factor Receptor

mTOR: mammalian Target Of Rapamycin

LN: Lymph node

NET: Neoadjuvant Endocrine Treatment

pCR: pathological Complete Response

PEPI: Preoperative Endocrine Prognostic Index

PI3K: Phosphatidylinositide 3 Phosphate

PR: Progesterone Receptor

Rb: Retinoblastoma

RCB: Residual Cancer Burden

ROR: Risk Of Recurrence

SLN: Sentinel Lymph Node

SPC: Summary of Product Characteristics

1. RATIONALE OF THE TRIAL

1.1. Neoadjuvant systemic therapy in ER+ breast cancer

ER+ ("luminal") breast cancer accounts for about 65-70% of all invasive breast carcinomas in Western Countries [1]. Primary chemotherapy or primary endocrine therapy are current options for patients bearing HR-positive Her2-negative breast cancer not candidate for immediate conservative surgery due to large tumor size.

ER expression is a negative prognostic factor for pathological response after primary chemotherapy among breast cancer patients. Pathological response to primary chemotherapy in luminal breast cancer is highly heterogeneous, and varies from 2% to no more than 20%. However, metaanalyses have shown an average pCR rate of 8% [2] and GeparTrio data have demonstrated that luminal B breast cancer with a Ki67 comprised between 15 and 35% have a pCR rate of 10% [3]. A recent review of 12 international trials involving almost 12 000 patients has confirmed the value of pCR as defined by ypT0/is ypN0 as a powerful prognostic factor for both event-free and overall survival [4]. However, this association between pathological complete response and long-term outcomes was strongest in patients with triple-negative breast cancer (EFS: HR 0·24, 95% CI 0·18–0·33; OS: 0·16, 0·11–0·25) and in those with HER2-positive, hormone-receptor negative tumours who received trastuzumab (EFS: 0·15, 0·09–0·27; OS: 0·08, 0·03, 0·22). It is striking that pCR has a much less powerful prognostic value for luminal (ER+) breast cancer, whereas this subtype usually exhibits the lowest pathological response rates.

The alternative systemic primary therapy in ER+ breast cancer is endocrine therapy. Clinical response with neoadjuvant endocrine treatment (NET) is roughly 50% [5-8]. It has also been suggested that NET may yield a higher rate of breast conserving surgery than chemotherapy in ER+ disease [6], although pCR remains under 10% in both instances. A duration of 16-18 weeks of neoadjuvant endocrine therapy is a commonly accepted schedule and has been validated in randomized studies [9]. Beyond clinical response, no pathological or biological parameter of response has been validated so far. The most commonly evaluated biomarker is the Ki67 level at baseline, the best Ki67 responses being observed for patients with a high baseline value, however with no standardized cutoff [10,11]. The most promising parameter for evaluating the response to NET has been proposed by Ellis et al in the P024 trial, which assessed on a dynamic basis (i.e. pre- and post-NET) a Preoperative Endocrine Prognostic index ("PEPI") combining clinical (T and N) and biological (ER and Ki67) parameters [9]. The PEPI score is very significantly associated with long term prognosis. However, it has never been validated in further prospective trials. It has also been shown that no endocrine therapeutic class yields better results than the others [9,10,12].

It has also been highlighted over the years that prognosis in early breast cancer is not only related to common clinical (pT, pN, M) and biological (ER, PR, proliferation, HER2) features, but also to how we look at prognosis. pCR and PEPI are closely correlated to short term prognosis, i.e. the risk of relapse or death in the 5 years after diagnosis. The unsolved issue for ER+ disease is the long term prognosis, where no surrogate after neoadjuvant systemic therapy has been unveiled so far.

Taken together, these data strongly suggest that conventional chemotherapy may not be the best means to achieve early results with a long term impact, and they also underline the need for alternative approaches in ER+ breast cancer. We need new and reproducible evaluation criteria, as well as new therapeutic approaches in this very common setting.

1.2. PAM50 signature in luminal breast cancer

Beyond classical parameters, genomic signatures have emerged during the last 10 years as a new and additive means to evaluate more precisely long term prognosis, and in some instances the amount of benefit from chemotherapy or endocrine therapy in the adjuvant setting [13].

More specifically, predictive factors for the long term benefit of adjuvant endocrine therapy have been recently enriched by second generation genomic signatures, namely Endopredict [14] and PAM50 [15,16]. The PAM50 signature has proven a superior long term predicting ability than OncotypeDx in luminal breast cancer [17].

As a second-generation FFPE based multigene expression assay, the PAM50 test (50 discriminator genes + 8 controls) was developed to identify intrinsic breast cancer subtypes [luminal A (LumA)/ B (LumB), HER2-enriched, basal-like], which reflect the underlying biology associated with ER and HER2 pathways, and in addition includes

proliferation genes and markers of the basal phenotype. The terminology of intrinsic subtypes was adopted by the 2011 St Gallen Consensus Conference to describe the paradigm for making treatment decisions in patients with EBC. Luminal subtypes A and B are the most common subtypes of breast cancer in the clinically hormone receptor-positive population. LumA tumors, characterized by lower expression of genes associated with cell cycle activation and ERBB2 have significantly lower rates of recurrence (i.e. better prognosis) when compared with LumB, which can be quantified as a ROR-Score. It has also been shown that PAM50 reliably identifies about 10% of non-luminal tumors on ER+ disease from FFPE samples, with a long term prognosis impact [18].

The PAM 50 signature has now been evaluated on numerous series of patients treated either in the adjuvant or neoadjuvant setting, and either by neoadjuvant chemotherapy or NET. It has been shown to highly correlate with Residual Cancer Burden (see below) and pCR in patients treated with neoadjuvant chemotherapy [19-21], as well as with the PEPI score in patients treated with NET [12].

Table 1: PAM50 results in neoadjuvant studies

Author	Reference	Population	sample	Main results
Parker	JCO 2009	189 test<br>761+133 validation chemotherapy	Frozen +FFPE	- Subtypes<br>- ROR S and ROR C<br>- Response to CT
Ellis	JCO 2011	Z1031 : anastrozole vs exemestane vs letrozole	Frozen	Correlation PEPI and RCB
Esserman	BCRT 2012	I-SPY1	FFPE	Correlation PAM50 and RCB
Cheang	CCR 2012	MA5	FFPE	Subtyping predicts response to chemo and prognosis

However, the PAM50 signature and its results, i.e. individual subtyping and attribution of a ROR score have not been prospectively tested. We propose here to combine these results in a randomized parallel study comparing neoadjuvant chemotherapy and an endocrine based neoadjuvant therapy, in a series of luminal breast cancer as assessed by PAM50.

1.3. Palbociclib in luminal breast cancer

Despite an initial rather good prognosis, a large amount of early ER+ breast cancer patients relapse over time. Long term prognosis is closely associated with initial tumor burden parameters (T, N) as well as biological features which can be captured with second generation genomic signatures such as PAM50 [17]. Resistance mechanisms to endocrine treatments are diverse and may involve various pathways such as PI3K/mTOR, IGF pathway, non-genomic ER signalling, etc (reviewed in [22]). Several attempts to combine endocrine treatments and pathway targeting in advanced ER+ breast cancer have generated mitigated results, yielding in 2012 to the controversial approval of everolimus in combination with exemestane. Of note, no other targeting (IGFR, EGFR, src, epigenetics…) has produced clinically meaningful results. Recently, involvement of CyclinD1 and related kinases CDK4 and 6 in endocrine resistance have been highlighted [23].

Palbociclib is an oral cyclin-dependent kinase (CDK) 4/6 inhibitor that has been under investigation in Phase I and II clinical trials in multiple indications. Palbociclib prevents cell cycle progression from G1 to S phase and has shown antitumor activity in multiple preclinical models, including in estrogen receptor-positive (ER+) luminal breast cancer

cell lines. Furthermore, pre-clinical exploration using a breast cancer cell line panel has demonstrated that presence of retinoblastoma (Rb) protein and upregulation of cyclin D1 as well as decreased CDKN2A (p16) were associated with sensitivity to palbociclib as well as with its effects upon cell cycle and growth inhibition. These gene expression findings were also associated with the luminal subtype versus basal-like subtype of BC. These results, together with published data on the interaction of estrogens and CDKs and the important role of cell cycle-related proteins in the genesis and maintenance of breast cancer, led to the initiation of a randomized Phase II clinical trial (A5481003 – Paloma-1) investigating the antitumor activity of palbociclib in combination with letrozole and single-agent letrozole in the first-line treatment of ER(+)/HER2(-) ABC patients. The Phase II study was divided into 2 parts. In Part 1, patient selection was based only on ER/HER2 status while in Part 2, patients were additionally prospectively selected taking into account tumor CCND1 amplification and/or CDKN2A (p16) loss. After a median follow-up of 16.5 months, preliminary results from Part 1 of this Phase II trial suggest that the combination of palbociclib with letrozole is superior to letrozole alone in the selected patient population as demonstrated by prolonged progression-free survival (median 18.2 months vs 5.7 months, respectively), and improved objective response and disease control rates (52% vs 32% and 76% vs 47%, respectively) in patients treated with the combination [24]. The combination therapy was generally well tolerated when compared to letrozole alone with AEs similar to those seen with palbociclib and letrozole when administered alone. Uncomplicated neutropenia, leucopenia, and fatigue were the most frequent adverse events, and the most commonly reported Grade 3 treatment-related adverse events were neutropenia (54%) and leucopenia (21%) in patients treated with the combination therapy. Grade 4 events included neutropenia and fatigue each reported for 6% of patients treated with palbociclib + letrozole. No Grade 4 events were reported in the letrozole alone arm. Treatment-related Grade 1/2 AEs reported more frequently in patients in the palbociclib + letrozole arm compared with the letrozole alone arm included leukopenia, anemia, fatigue, alopecia, arthralgia, nausea, neutropenia, and thrombocytopenia. Hot flushes were the most common Grade 1/2 treatment-related AEs reported in patients enrolled in the letrozole alone arm.

Overall, 3 (9%) patients in the palbociclib + letrozole arm and 1 (3%) patient in the letrozole alone arm discontinued the Phase II Part 1 of Study A5481003 due to AEs including 1 patient with Grade 4 fatigue (not related to PD-0332991) and 2 patients with Grade 3 neutropenia in the palbociclib + letrozole arm and 1 patient with Grade 2 nausea in the letrozole alone arm. The median duration of treatment was 13.7 months in the palbociclib + letrozole arm vs 5.4 months in the letrozole alone arm, with palbociclib dosing interruptions and dose reductions due to AEs reported in 61% and 39% of patients enrolled in the palbociclib + letrozole arm respectively. The median duration of dosing interruptions was 4.5 days, and the median time to first dosing interruption was 55.5 days. Despite the dosing interruptions and dose reductions, the median dose intensity for palbociclib was 87% across all cycles. Palbociclib is currently under evaluation in preoperative and neoadjuvant settings (no data yet available but for safety).

Based on these striking results on progression free survival and on tumor response in both initially and secondary resistant patients, combined with a favourable safety profile, we postulate that the letrozole-palbociclib combination should be evaluated in the neoadjuvant setting and compared to standard chemotherapy in luminal BC.

1.4. The Residual cancer burden

As recalled above, the most widely evaluated parameter in the neoadjuvant setting in pathological response assessed on both breast primary tumor and axillary lymph nodes [5]. The corresponding criterion for NET, the PEPI score, has not been prospectively and independently validated yet. Within this context, the residual cancer burden (RCB), an innovative mode of evaluation of the residual tumor after neo-adjuvant chemotherapy has been published initially by Symmans in 2007 [25]. The RCB was developed first on a cohort of 241 patients treated by paclitaxel 4 cycles followed by FAC, 4 cycles, then on a FAC only cohort (4 courses only), and a third Paclitaxel-FAC cohort. 19% of patients had Her2+ tumors, 29% triple-negative and 52% HR+ Her2-. The prognostic performance of RCB is stable among the three cohorts, whether on RFS or OS, with an HR around 2 and a c-index (AUC of 0.74. It therefore allows reliable prediction of prognosis. Among HR+ Her2- tumors, 10% had RCB 0 (pCR), 13% RCB I, 60% RCB II, 17% RCB III. Most importantly, RCB I tumors harbour the same long term prognosis than that of pCR (RCB 0) tumors, including in the 4 courses FAC validation cohort. These data have recently been validated at ten years of follow up [26].

Of note, the RCB has been used as an endpoint in the most recent neoadjuvant trials such as I-SPY1 [20]. In this trial, the RCB 0-I is consistently observed in ROR-S low or intermediate patient at an average 20% rate, in line with the initial report and recent validation by Symmans group.

Table 2: PCR and RCB (%) according to ROR-S and Ki67 status in I-SPY1 trial

		pCR	RCB 0-1			pCR	RCB 0-1
ROR-S	Low	6	18	Ki-67	-10	7	14
	Interm	17	24		10-20	10	18
	High	36	43		20+	35	43
	P value	0.006	0.06			<0.001	0.005

So far, the RCB has not been evaluated in NET treated patients.

1.5. Summary of rationale

We propose in the present study an innovative approach, combining the most recent therapeutic opportunities in high risk ER+ breast cancer with the most recent and innovative diagnostic approaches such as the PAM50 signature and the RCB tumor response evaluation method. In line with the most recent recommendations on targeted anticancer therapies, we have designed a parallel phase II randomized trial with early stopping rules [27], which will able in the meantime to build a unique prospective collection of tumor tissue, pre- and post-treatment.

2. TRIAL'S OBJECTIVES

2.1. Main Objective

The main objective of this trial is to evaluate the ability of each treatment strategy to provide RCB 0-I histological tumor response at surgery (local assessment) in luminal A N+ and luminal B patients subgroup.

2.2. Secondary Objectives

2.2.1 Efficacy

- To evaluate clinical responses in each arm and in each subgroups (luminal A N+ and luminal B; luminal A N-), as defined by clinical and ultrasound examination.

- To evaluate the rates of breast conservation therapy according to each treatment scheme.

- To evaluate the progression free survival (PFS) and invasive disease free survival (iDFS) at 3 years follow-up.
- To evaluate RCB 0-1 pathological tumor response at central reading.

2.2.2 Toxicity

- To assess the safety of each treatment strategy according to CTC-AE v 4.0.

2.2.3 BIOLOGY

- To assess the positive predictive value of ROR on clinical and pathological tumor response in both treatment arms.

- To evaluate the ability of predefined biomarkers to predict for clinical and pathological response in each arm, such as p53 mutation status, p16/CDKN2A, RB1 and CCND1 status, as well as other proliferation and senescence biomarkers.

3. METHODOLOGY

Open-label, randomized, multicenter, parallel exploratory phase II study, comparing sequential standard chemotherapy (3 FEC 100 cycles followed by 3 cycles Docetaxel 100) and a same duration letrozole + palbociclib combination as neoadjuvant treatment of stage II-IIIA PAM 50 defined Luminal A-Node+/Luminal B breast cancer.

Newly patient diagnosed with ER+, Her2-negative, stage II-III breast cancer will be tested for PAM50 signature. Tumor tissue availability is required for patient participation. Only luminal A N+ or Luminal B patients according to the PAM50 result will be eligible and randomized to receive either Letrozole plus Palbociblib or standard chemotherapy (3 FEC and 3 Docetaxel).

<u>Study design:</u>

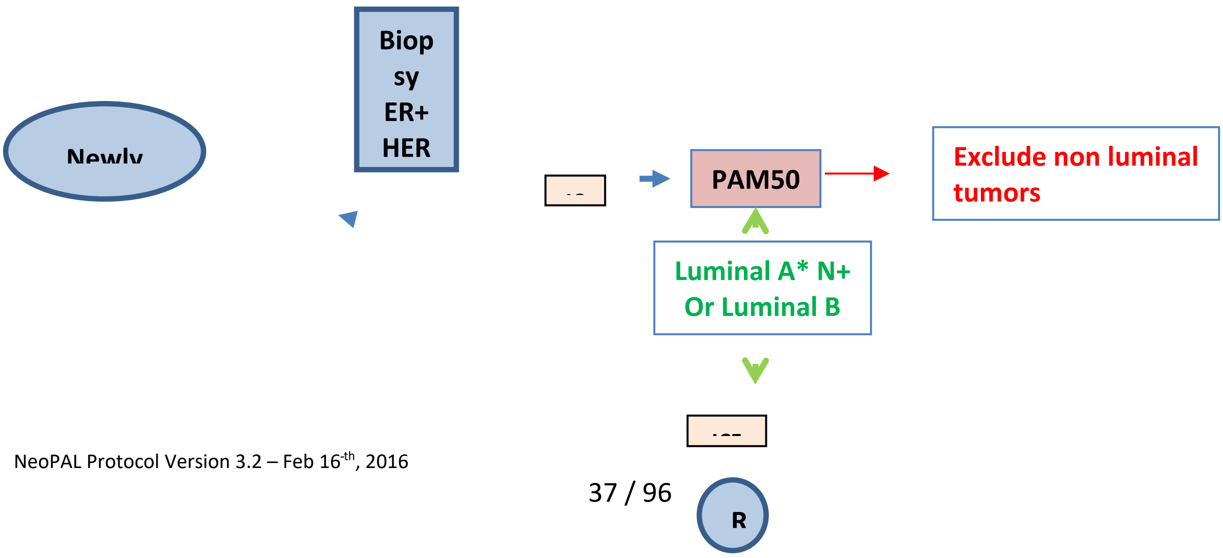

4. PATIENT SELECTION

4.1. Inclusion Criteria

All the following conditions are to be fulfilled:

1) Aged ≥ 18 years, post-menopausal women according to the following criteria
   Age > 60 years,

   Bilateral ovariectomy,

   Age ≤ 60, with an uterus and presenting an amenorrhea of more than 12 months,

   Age ≤ 60 without an uterus and FSH > 20 IU/L

2) Newly diagnosed and operable unilateral invasive breast cancer, not candidate or uncertain for breast conservation –

Note: Multicentric/multifocal tumors are allowed provided a maximum of 3 lesions are present, and all share the same characteristics: ER Allred 4, Her2- (PAM50 will be performed in the largest lesion)

3) Stage II-IIIA
4) Assessment of nodal status available (Ultrasound guided FNA or biopsy if necessary)
5) Non metastatic, M0
6) ER-positive by IHC (Allred Score ≥ 4)
7) HER2-negative by IHC (score 0 or 1+) and/or FISH/CISH
8) PAM50 (Prosigna™) centralized evaluation available and:
   - *Either Luminal A AND proven nodal involvement (cytology or histology), or Luminal B for randomized patients*
   - *Or Luminal A N- for non randomized patients*
9) ECOG 0-1
10) No prior systemic therapy for the present tumor
11) Adequate renal, hepatic, and hematopoietic functions as defined by the following criteria:
    - *Absolute Neutrophil Count (ANC) ≥1,500/mm^3 or ≥1.5 x 10^9/L*
    - *Platelets ≥100,000/mm3 or ≥100 x 10^9/L*
    - *Hemoglobin ≥9 g/dL*
    - *Serum Aspartate Transaminase (AST) and serum Alanine Aminotransferase Transaminase (ALT) ≤2.5 x upper limit of normal (ULN)*
    - *Alkaline phosphatase ≤2.5 x ULN*
    - *Total serum bilirubin ≤1 x ULN*
    - *Serum creatinine ≤1.5 x ULN or estimated creatinine clearance ≥60 mL/min as calculated using the method standard for the institution*
12) Adequate cardiac functions, including:
    - *12 Lead electrocardiogram (ECG) with normal tracing or non-clinically significant changes that do not require medical intervention.*
    - *QTc interval ≤480 msec*
    - *No history of Torsades de Pointes or other symptomatic QTc abnormality.*
13) Willingness and ability to comply with scheduled visits, treatment plan, laboratory tests, and other trial procedures
14) Signed informed consent form and health insurance coverage.

4.2. Non-inclusion Criteria

1) Non operable, bilateral, T4 or metastatic breast cancer
2) Limited T2 breast cancer immediately accessible to conservative surgery
3) Previous homolateral breast cancer (including in situ carcinoma), and/or contralateral breast cancer except if treated by surgery +/- radiation therapy alone without any systemic treatment
4) Previous hormone replacement therapy (HRT) stopped less than 2 weeks before beginning of treatment
5) Previous use of SERMs such as raloxifene
6) Any surgery (not including minor procedures such as lymph node biopsy, primary tumor core biopsy, fine needle aspiration) within 4 weeks of start of study treatment; or not fully recovered from any side effects of previous procedures.
7) Diagnosis of any previous malignancy within the last 5 years, except for adequately treated basal cell carcinoma, or squamous cell skin carcinoma, or in situ cervical carcinoma

8) Diagnosis of hypokalemia

9) History of any previous anti-cancer chemotherapy and any previous treatment using an Aromatase Inhibitor.

10) Concurrent administration of herbal preparations as complementary medicine.

11) Any clinically significant gastrointestinal abnormalities, which may impair intake, transit or absorption of the study drugs, such as the inability to take oral medication in tablet form and malabsorption syndrome

12) Patient with any psychological, familial, social or geographical condition which could potentially hamper compliance with the study protocol and follow-up schedule; those conditions should be discussed with the patient before registration in the trial.

5. RANDOMIZATION

After provision of written informed consent, completion of baseline assessments and confirmation that all inclusion / exclusion criteria have been met, Luminal A N-positive or Luminal B patients will be randomized in a 1:1 ratio to either Arm A (experimental arm: Palbociclib (1 capsule /day) added to Letrozole (1 tablet /day)) or Arm B (control arm: standard chemotherapy (3 cycles of FEC 100 followed by 3 cycles of Docetaxel 100)).

To randomize their patient, the investigator will have to connect to a specific web site using the login and password given during the site initiation visit.

Randomizations will be equally balanced between the two arms and will be stratified based on:

- T2 versus T3
- PAM 50 luminal A vs luminal B

Luminal A N-negative patients will not be randomized but will be analyzed independently as a prospective cohort.

6. TREATMENTS

The investigational drugs used in the course of this trial are Palbociclib and Letrozole. 5 Fluoro-uracile, Epirubicine, Cyclophosphamide, Docetaxel.

6.1. Palbociclib

Palbociclib (PD-0332991) is an oral selective inhibitor of the cyclin-dependent kinases CDK4 and CDK6.

Palbociclib prevents cell cycle progression from G1 to S phase and has shown antitumor activity in multiple preclinical models, including in estrogen receptor positive (ER+) luminal breast cancer cell lines. This activity has been confirmed in the Paloma-1 study.

6.1.1. Packaging, Labeling, Storage conditions

Palbociclib will be provided by UNICANCER.

This drug will be labeled in accordance with the guidelines of the appendix 13 of the EEC directive: Good Manufacturing Practices for the manufacture of investigational medicinal products (revised and adopted in 31 January 2010 by the European Commission).

Labeling and distribution will be performed by SODIA, Avenue Robert Schuman 51100 REIMS - FRANCE

Palbociclib capsules:. Palbociclib will be supplied as capsules containing 75mg, 100mg or 125 mg equivalents of Palbociclib free base.

The sponsor will provide the oral drug formulation to sites in HDPE bottles containing 23 capsules.

Trial supplies will be labeled in accordance with the clinical trials specific requirements and regulations.

Storage conditions:

Palbociclib capsules should be stored in the original closed packaging, protected from light and moisture, at controlled room temperature (15-25°C, 59-77°F) in their original container.

Medication should be kept in a secured locked area at the study site in accordance with applicable regulatory requirements. Returned medication should be stored separately from medication that needs to be dispensed.

These instructions should also be made clear to the patient for storage and self-administration of medication at home.

6.1.2. Dispatch, distribution and accounting

The distribution of the Palbociclib supplied to the health care centers' pharmacies by the sponsor will be performed by SODIA in conformity with the Good Distribution Practices, under sponsor's supervision and responsibility.

The pharmacist of the health care center will acknowledge receipt of all shipments by returning to the distributor a duly completed receipt.

Palbociclib will have to be stored in a locked room with limited access and in accordance with the recommendations of the manufacturer (see section 6.2).

It is the responsibility of the Investigator to ensure that Palbociclib is only dispensed to study subjects. Palbociclib must be dispensed only from official study sites by authorized personnel according to local policy.

Site personnel must ensure that patients clearly understand the directions for self-medication. Patients should be given a sufficient supply to last until their next study visit. Unused drug and/or empty bottles should be returned to the site at the next study visit. Returned unused medication MUST NOT be re dispensed to patient.

Patients should be instructed to keep their medication in the bottles provided and not transfer it to any other container. Due to possible unknown hazards associated with topical and environmental exposure to experimental agents, capsules must not be opened and/or emptied into any vehicle for oral ingestion; capsules must be swallowed intact.

The pharmacist of the health care center will keep accurate records of the drugs delivered, used, unused and/or returned by the patient.

The sponsor's clinical research assistant will be responsible for verifying the accounting records for the supplied medicinal products ensuring that accountability forms are validated and signed by the pharmacist of the health care center prior to drug destruction.

Drug destruction:

Unused Palbociclib will be destroyed on site. It is Investigators responsibility to ensure that arrangements have been made for the disposal, that written authorization has been granted by the Sponsor's representative, procedures for proper disposal have been established according to applicable regulation and guidelines and institutional procedures, and appropriate records of the disposal have been documented..

6.1.3. Palbociclib administration

Detailed recommendations are available in the Investigator Brochure. Shortly, the tablet(s) should be taken orally with a glass of water, approximately at the same time each day, during a meal, preferably in the morning. Patients should be instructed to swallow Palbociclib capsules whole and not to chew them prior to swallowing. No capsule should be ingested if it is broken, cracked, or otherwise not intact. Patients should be encouraged to take their dose at approximately the same time each day. Patients should be instructed to record daily administration of the study drugs in a patient diary.

Palbociclib will be administered together with letrozole.

Patients experiencing investigational product related toxicity may have their dose modified according to section 7. If vomiting occurs, no attempt should be made to replace the vomited dose. Patients should be instructed that if they miss a dose on one day they should not take any extra doses the next but instead to contact the study center as soon as possible to ask for advice.

6.2. Letrozole

Letrozole will be prescribed in the therapeutic indication of neo-adjuvant treatment of postmenopausal women with hormone receptor positive, HER-2 negative breast cancer.

The Sponsor will provide commercially available letrozole 2.5 mg film coated tablets.

This drug will be labeled in accordance with the guidelines of the appendix 13 of the EEC directive: Good Manufacturing Practices for the manufacture of investigational medicinal products (revised and adopted in 31 January 2010 by the European Commission).

Labeling and distribution will be performed by SODIA, Avenue Robert Schuman 51100 REIMS - FRANCE

Letrozole tablets must be stored according to the instructions detailed in the local package insert. Complete information about letrozole formulation can be found in the Summary of Product Characteristics (SPC) for Femara© (see appendix 5).

To ensure adequate records, letrozole tablets will be accounted for as instructed by the sponsor. Patients are requested to return previously dispensed containers as well as their completed patient diary to the clinic at each visit for accountability purposes even if they will not be issued with new medication at that visit.

6.3. Chemotherapy

The standard treatment recognized nationally for Her2 negative, N+ tumors is 3 cycles of FEC 100 followed by 3 cycles of TAXOTERE according to the schema of the PACS 01 study.

The Sponsor will not provide chemotherapy. FEC100 and TAXOTERE will be taken from stocks of site's pharmacy.

The pharmacist of the health care center will keep accurate records of the chemotherapy delivered on the forms provided by the sponsor.

The sponsor's clinical research assistant will be responsible for verifying the accounting records for the chemotherapy ensuring that accountability forms are validated and signed by the pharmacist of the health care center prior to drug destruction.

Complete information about chemotherapy formulation can be found in the Summary of Product Characteristics (SPC) (see appendix 5).

6.4. General Rules

For both Palbociclib and letrozole:

- Patients who miss a day's dose entirely must be instructed NOT to "make it up" the next day.
- Patients who vomit any time after taking a dose must be instructed NOT to "make it up," and is to resume treatment the next day as prescribed.

- Patients who inadvertently take 1 extra dose during a day must be instructed to skip the next day's dose.

6.5. Course of the Treatment

Patients will begin trial treatment within 15 days of randomization, and will receive one of following treatments:

Arm A:

Palbociclib 125 mg per day (1 capsule/day) taken together with Letrozole 2.5 mg per day.

- Palbociclib will be administered orally once a day for 21 days of every 28-day cycle followed by 7 days off treatment until the day prior to surgery, for total of about 19 weeks (see treatment schedule, appendix 2);
- Letrozole will be administered orally once a day until the day prior to surgery, for total of about 19 weeks (see treatment schedule, appendix 2).

or

Arm B:

Standard chemotherapy (3 cycles of FEC100 followed by 3 cycles of Docetaxel100)

Cycles 1 to 3 : FEC 100

3 courses of FEC 100 will be administered every 3 weeks:

- 5FU: 500 mg/m2 (infusion as recommended by the RCP)
- Epirubicine 100 mg/m2 (infusion as recommended by the RCP)
- Cyclophosphamide 500 mg/m2 (infusion as recommended by the RCP)

Theoretical Dates : D1 (w1), D22 (w4), D43 (w7)

Cycles 4 to 6 :

3 courses of docetaxel will be administered every 3 weeks, beginning 3 weeks after the last administration of FEC100:

- Docetaxel : 100 mg/m2 (infusion as recommended by the RCP)

Theoretical Dates : D64 (w10), D87 (w13), D108 (w16). See treatment schedule (appendix 2).

The chemotherapy courses will be reconducted according to current chemotherapy management guidelines, as per each center (refer to section 6.6.3). More specifically, hematological toxicity will be managed as follows:

- Neutrophil granulocyte count should be ≥ 1000/mm3
- Platelets count should be ≥ 100 000/mm3

Chemotherapy may be delayed up to 14 days. Patients should be excluded from the study if hematological recovery is not complete at day 35.

Granulocyte colony stimulating factors (G-CSF) is highly recommended as primary or secondary prophylaxis according to current guidelines, at days 6-13 after each course.

Docetaxel acute reactions prophylaxis will be given according to local procedures

Treatment duration: Treatment will be pursued for 19 weeks.

7. Dose modifications and management of specific toxicities

7.1. General considerations

In the event of significant treatment-related toxicity, treatment may be interrupted or delayed and/or reduced as described below. In the event of multiple toxicities, dose modification should be based on the worst toxicity observed. Patients are to be instructed to notify Investigators at the first occurrence of any adverse sign or symptom.

Dose modifications may occur in three ways:

- Within a cycle: dosing interruption until adequate recovery and dose reduction, if required, during a given treatment cycle;
- Between cycles: next cycle administration may be delayed due to persisting toxicity when a new cycle is due to start;
- In the next cycle: dose reduction may be required in a subsequent cycle based on toxicity experienced in the previous cycle.

Patients discontinuing Palbociclib treatment due to treatment-related toxicity should continue on the active treatment phase of the study receiving letrozole monotherapy.

7.2. Palbociclib

The undesirable effects that may occur with Palbociclib are listed in the Investigator Brochure given during the site initiation visit.

7.2.1. Dosing Interruptions

Patients experiencing the following adverse events should have their treatment interrupted/delayed:

- Uncomplicated Grade 3 neutropenia (ANC<1000/mm^3);
- Grade 3 neutropenia (ANC<1000/mm^3) associated with a documented infection or fever ≥38.5°C;
- Grade 4 neutropenia (ANC<500/mm^3);
- Grade 3-4 thrombocytopenia (Platelet count <50,000/mm^3);
- Grade ≥3 non-hematologic toxicity (including, nausea, vomiting, diarrhea, and hypertension only if persisting despite optimal medical treatment);
- Grade 3 QTc prolongation (QTc ≥501 msec on at least two separate ECGs).

Appropriate follow up assessments should be done until adequate recovery occurs as assessed by the Investigator. Criteria required before treatment can resume are described in the next section.

Doses may be held as needed until toxicity resolution. Depending on when the adverse event resolved, a treatment interruption may lead to the patient missing all subsequent planned doses within that same cycle or even to delay the initiation of the subsequent cycle.

If the adverse event that led to the treatment interruption recovers within the same cycle, then re-dosing in that cycle is allowed. Doses omitted for toxicity are not replaced within the same cycle.

In the event of a treatment interruption for reasons other than treatment-related toxicity (eg, non-cancer related surgery) lasting >2 weeks, treatment resumption will be decided in consultation with the sponsor.

7.2.2. Dose Delay

Retreatment following treatment interruption for treatment related toxicity or at the start of any new cycle may not occur until all of the following parameters have been met:

- Platelet count ≥50,000/mm^3;
- ANC ≥1000/mm^3 and no fever;
- Grade 3 or higher treatment-related non-hematologic AEs (including, nausea, vomiting, diarrhea, and hypertension only if persisting despite optimal medical treatment), with the exception of alopecia, have recovered to Grade ≤ 1 or baseline (or, at the investigator’s discretion, Grade ≤ 2 if not considered a safety risk for the patient).

- QTc <501 msec and potential reversible causes (eg, electrolyte imbalance, concomitant medications known to prolong QTc) corrected. If QTc remains above 480 msec, ECG should be monitored more frequently as per the investigator's best medical judgement until QTc≤480 msec.

If a treatment delay results from decline in hematologic parameters, the frequency of blood count assessments should be increased as clinically indicated.

If these parameters are met within 2 weeks of treatment interruption or cycle delay, Palbociclib may be resumed.

If these parameters have not been met after 2 weeks of dose interruption (including the scheduled 1 week off treatment) or 2 weeks of cycle delay, permanent discontinuation of Palbociclib treatment should be considered. Treatment resumption for patients recovering from treatment-related toxicity after > 2 weeks of treatment interruption or cycle delay but deemed to be deriving obvious clinical benefit per the investigator's best medical judgment is left at the investigator's discretion.

7.2.3. Dose reductions

No specific dose adjustments are recommended for Grade 1/2 treatment-related toxicity. However, investigators should always manage their patients according to their medical judgment based on the particular clinical circumstances.

Dose reduction of Palbociclib by 1 and, if needed, 2 dose levels (Table 3) will be allowed depending on the type and severity of toxicity encountered. Patients requiring more than 2 dose reductions will be discontinued from the study and entered into the follow-up phase. All dose modifications/adjustments must be clearly documented in the patient's source notes and Investigational product administration CRF.

Table 3. Palbociclib Dose Levels

**Dose Level**	**Palbociclib** (3 out of 4 weeks)
Starting dose	125 mg/d
-1	100 mg/d
-2	75 mg/d
Discontinue Study Treatment	

Once a dose has been reduced for a given patient, all subsequent cycles should be administered at that dose level, unless further dose reduction is required. Dose re-escalation is not allowed.

Patients discontinuing Palbociclib treatment due to treatment-related toxicity may continue on the active treatment phase of the study receiving letrozole monotherapy as per the investigator's discretion

Palbociclib recommended dose modifications for treatment related toxicities requiring treatment interruption/delay or persisting despite optimal medical treatment are described in Table 4.

Table 4:Palbociclib Dose Modifications for Treatment Related Toxicities Requiring Treatment Interruption/Delay or Persisting Despite Optimal Medical Treatment.

Toxicity	Restart Palbociclib at:
**Uncomplicated Grade 3 neutropenia** *(ANC<1000/mm^3)*	Same dose level
**Grade 3 neutropenia** *(ANC<1000/mm^3)* **associated with a documented infection or fever ≥38.5°C**	↓ 1 Dose Level
**Grade 4 neutropenia** *(ANC<500/mm^3)*	↓ 1 Dose Level
**Grade 3-4 thrombocytopenia** *(Platelet count <50,000/mm^3)*	↓ 1 Dose Level
**Grade ≥3 non-hematologic toxicity** *(including, nausea, vomiting, diarrhea, and hypertension only if persisting despite optimal medical treatment)*	↓ 1 Dose Level

- **QTc prolongation management**

In the event of QTc prolongation of, possible alternative reversible causes such as serum electrolytes abnormalities, or usage of concomitant medications with the potential to prolong the QTc interval should be evaluated.

If such reversible causes are identified, then they should be corrected accordingly (ie, correction of electrolyte abnormalities with supplements to within normal limits and/or discontinuation (if possible) of concomitant medications known to prolong the QT interval).

Management guidelines in case of QTc prolongation is described in Table 5.

Table 5:Management guidelines in case of QTc Prolongation

	Toxicity (NCI CTC Grade, Version 4.0)		
	**Grade 2 QTc prolongation**	**Grade 3 QTc prolongation**	**Grade 4 QTc prolongation**
**Reversible cause identified**	Treat reversible cause<br><br>Initiate more frequent ECG monitoring according to investigator's best medical judgment until QTc≤480 msec<br><br>Continue at the same dose level (1)	Treat reversible cause<br><br>Withhold treatment until QTc<501 msec<br><br>Resume treatment at the same dose level<br><br>Monitor ECG more frequently as per investigator's best medical judgment until QTc≤480 msec.	Permanently discontinue.<br><br>Monitor ECG more frequently (continuous) in hospital until the advice of a cardiologist

**No reversible cause identified**	Initiate more frequent ECG monitoring according to investigator's best medical judgment until QTc≤480 msec<br><br>Continue at the same dose level (1)	Withhold treatment until QTc<501 msec<br><br>Resume treatment at the next lower dose level(2)<br><br>Monitor ECG more frequently as per investigator's best medical judgment until QTc≤480 msec.	Permanently discontinue.<br><br>Monitor ECG more frequently (continuous) in hospital until the advice of a cardiologist

1. If the QTc remains above 480 msec more than 2 cycles or if Grade 2 QTc prolongation recurs in the absence of other alternative causes or despite correction of alternative causes, dose adjustment and/or discontinuation should be considered in consultation with a cardiologist and the study medical monitor, taking into account the emerging safety data from palbociclib trials and the investigator's best medical judgment.
2. If the Grade 3 QTc prolongation occurs again after one dose reduction, further dose adjustment and/or discontinuation should be discussed with study medical monitor in consultation with a cardiologist, taking into consideration the emerging safety data from palbociclib trials and the investigator's best medical judgment.

7.3. Letrozole

No dose adjustment for letrozole is permitted but dosing interruptions are allowed.

Treatment interruption for letrozole related toxicities will be performed as per the investigator's best medical judgment.

7.4. Chemotherapy

Chemotherapy is conventional and will generally be managed according to standard procedures. More specifically, management of hematological, non-hematological and specific docetaxel side effects are detailed below.

Management guidelines in case of hematological and non-hematological Toxicities is described in table 6 and 7 respectively.

Docetaxel specific toxicities are described in table 8.

Table 6: Management guidelines in case of Hematological Toxicity

Neutrophil count ≥ 1000/mm3 **and** Platelets ≥ 100 000/mm3	Continue chemotherapy (CT)
Neutrophil count ≤ 1000/mm3 **or** Platelets ≤ 100 000/mm3	- Delay CT for 7 days (D28)<br>- G-CSF for all subsequent courses
Neutrophil count ≤ 1000/mm3 **or** Platelets ≤ 100 000/mm3 At Day 28	- Extended CT delay<br>- D35 is the longest permitted delay
Febrile neutropenia	
neutrophil < 500 / mm3 **and** fever > 38.5°c	- Antibiotics as per local procedures<br>- G-CSF<br>- CT delay (see above)
No recovery at D35 despite G-CSF use	- 25% CT dose reduction<br>- G-CSF for all subsequent courses
Anemia (Hb < 11g/dl)	Management as per local procedures

Table 7: Management guidelines in case of Non Hematological Toxicities (Except Alopecia)

Grade II peripheral neuropathy	- CT delay until downgrading to grade ≤1 (max 14 days)<br>- CT reconducted with -25% dose
Grade III ou non healed grade II neuropathy	- CT to be stopped<br>- Treatment at investigator's discretion.<br>- Patient withdrawn from study
Grade III – IV mucositis	- CT delay until downgrading to grade ≤1 (max 14 days)<br>- CT reconducted with -25% dose
Other grade III toxicities	CT delay until downgrading to grade ≤1 (max 14 days)
Other grade IV toxicities	- CT to be stopped<br>- Treatment at investigator's discretion.<br>- Patient withdrawn from study

Table 8: Docetaxel specific toxicities

Mild or moderate hypersensivity reactions	- Surveillance until resolution; Symptomatic care at investigator's discretion, No delay nor dose modification
Severe hypersensivity reactions<br>(bronchospasma, urticaria, hypotension < 80mmHg, angioedema)	- Stop docetaxel infusion and immediate resuscitation care<br>- Treatment can be reconducted within 24h after complete disappearance of symptoms and appropriate premedication
Peripheral oedema	- Symptomatic care at investigator's discretion<br>- Docetaxel my be reconducted at investigator's discretion
Transaminases > 1.5 x ULN **AND** PAL > 2.5 x ULN	25% dose reduction
Bilirubine Totale > 1,5 x LSN **OR** Transaminases > 3.5 x LSN et PAL > 6 x LSN	Stop docetaxel

8. Concomitant treatments

8.1. Authorized Concomitant Treatments

The use of other concomitant medication/therapy deemed necessary for the care of the patient is allowed (other than those described below).

The prescriptions that are not related to cancer treatment will be noted in the case report form.

The following treatments are permitted throughout the duration of the active treatment phase:

Standard therapies for preexisting medical conditions, medical and/or surgical complications, and palliation. Any medication intended solely for supportive care (eg, analgesics, antidiarrheals, antidepressants) may also be used at the investigator's discretion. All medications should be recorded.

Bisphosphonates and receptor activator of nuclear factor kappa B ligand (RANKL) inhibitors for the treatment of osteoporosis or management of existing bone metastases may be continued for patients who have been receiving them at a stable dose for at least 2 weeks prior to randomization. Initiation and/or dose increase during the study period is forbidden.

Hematopoietic growth factors (eg, granulocyte colony stimulating factor [G CSF]): Primary prophylactic use of granulocyte colony stimulating factors is not permitted for patients receiving Palbociclib but they may be used to treat treatment emergent neutropenia as indicated by the current American Society of Clinical Oncology (ASCO) guideline [27]. If neutropenic complications are observed in a cycle in which primary prophylaxis with CSFs was not received, secondary prophylaxis may be given at the discretion of the investigator, but only if dose reduction or delay are not considered to be a reasonable alternative.

8.2. Prohibited concomitant treatments

Patients must be instructed not to take any additional medications (over-the-counter or other products) during the study without prior consultation with the investigator. Any medications including herbal supplements, vitamins, or treatment taken by the patient from 28 days prior to the start of study treatment and up to 28 days following the last dose of investigational product and the reason for their administration must be recorded on the CRF.

• **Anticancer agents:** No additional investigational or commercial anticancer agents other than those mentioned in this study will be permitted during the active treatment phase. In general, any drugs containing "for the treatment of breast cancer" on the product insert are not permitted on study.

• **Strong CYP3A inhibitors/inducers (Table 9):** Palbociclib is metabolized to multiple metabolites in a qualitatively similar manner in rat, dog and human liver microsomes. In vitro, palbociclib is primarily metabolized by CYP3A4 enzymes. Co administration with drugs that are CYP3A inhibitors and inducers may change the plasma concentrations of palbociclib in humans.

- Co-administration with strong CYP3A inhibitors (e.g. amprenavir, atazanavir, boceprevir, clarithromycin, conivaptan, delavirdine, diltiazem, erythromycin, fosamprenavir, indinavir, itraconazole, ketoconazole, lopinavir, mibefradil, miconazole, nefazodone, nelfinavir, posaconazole, ritonavir, saquinavir, elaprevir, telithromycin, verapamil and voriconazole) should be avoided while on treatment. Patients treated with those drugs within the last 5 days prior to randomization cannot be included in the trial.

- Co-administration with strong CYP3A inducers (e.g. carbamazepine, felbamate, nevirapine, phenobarbital, phenytoin, rimidone, rifabutin, rifampin, rifapentin, and St. Johns wort) should be avoided while on treatment. Patients treated with those drugs within the last 5 days prior to randomization cannot be included in the trial.
- Co-administration with moderate CYP3A inhibitors (e.g. fluconazole, calcium channel blockers, benzodiazepines) and moderate inducers should also be avoided if possible, or otherwise subject to caution (e.g. increased frequency of safety monitoring).

• **Herbal** / alternative remedies, Sevilla orange, grapefruit and grapefruit juice affect cytochrome P450 and PgP activity and should therefore be avoided

• **Chronic immunosuppressive therapies** should be avoided, including systemic corticosteroids. Steroids given for physiological replacement, as anti-emetics or inhaled as well as short course of oral/topical steroids given for allergic reactions or asthma flares are allowed.

• **Erythropoietin** is not to be used. Any potential indication should be discussed with the investigator and the sponsor.

Table 9: Clinically relevant drug interaction: substrates, inducers and inhibitors of isoenzyme CYP3A.

Substrates		
**Macrolide antibiotics:**	**HMG CoA Reductase Inhibitors:**	
clarithromycin*	atorvastatin	
erythromycin	cerivastatin	
NOT azithromycin	lovastatin	
telithromycin*	NOT pravastatin	
	simvastatin	
**Anti-arrhythmics:**		
quinidine	**Steroid 6beta-OH:**	
	estradiol	
**Benzodiazepines:**	hydrocortisone	
alprazolam	progesterone	
diazepam	testosterone	
midazolam		
triazolam	**Miscellaneous:**	
	alfentanyl	LAAM
**Immune Modulators:**	aprepitant	lidocaine
cyclosporine	aripiprazole	methadone
tacrolimus (FK506)	buspirone	nateglinide
	cafergot	ondansetron
**HIV Protease Inhibitors:**	caffeine=>TMU	pimozide
indinavir*	cilostazol	propranolol
nelfinavir*	cocaine	quetiapine
ritonavir*	codeine- N-demethylation	quinine
saquinavir *	dapsone	risperidone
	dexamethasone	NOT rosuvastatin
**Prokinetic:**	dextromethorphan	salmeterol
cisapride	docetaxel	sildenafil
	domperidone	sirolimus
**Antihistamines:**	eplerenone	tamoxifen
astemizole	fentanyl	taxol
chlorpheniramine	finasteride	terfenadine
terfenadine	gleevec*	trazodone
	haloperidol	vincristine
**Calcium Channel Blockers:**	irinotecan	zaleplon
amlodipine		ziprasidone
diltiazem		zolpidem
felodipine		
lercanidipine		
nifedipine		
nisoldipine		
nitrendipine		
verapamil		

Inducers		
**HIV Antivirals:**<br>efavirenz<br>nevirapine	barbiturates<br>carbamazepine<br>glucocorticoids<br>modafinil<br>oxcarbazepine<br>phenobarbital	phenytoin*<br>pioglitazone<br>rifabutin*<br>rifampin*<br>St John's wort<br>troglitazone

Inhibitors			
**Strong**	**Moderate**	**Weak**	**Others**
**HIV Antivirals:**<br>indinavir*<br>nelfinavir*<br>ritonavir*<br>saquinavir*<br><br>clarithromycin*<br>fluvoxamine*<br>itraconazole*<br>ketoconazole*<br>nefazodone*<br>posaconazole*<br>telithromycin*<br>voriconazole*	aprepitant<br>erythromycin<br>fluconazole<br>grapefruit juice<br>verapamil<br>Seville orange (bitter orange) juice or product<br><br>diltiazem (moderate/weak)	cimetidine	amiodarone<br>NOT azithromycin<br>chloramphenicol<br>delaviridine<br>diethyl- dithiocarbamate<br>gestodene<br>imatinib<br>mibefradil<br>mifepristone<br>norfloxacin<br>norfluoxetine<br>star fruit (carambola)<br>troleandomycin

Based on: Ingelman-Sundberg M, Human drug metabolising cytochrome P450 enzymes: properties and polymorphisms, Naunyn Schmiedebergs Arch Pharmacol. 2004 Jan; 369(1):89-104. [http://www.medicine.iupui.edu/flockhart/clinlist.htm as of Aug 20, 2007 (version 4.0)]

* asterisk denotes strong inhibition/ induction

Please note:

- strong inhibitor implies that it can cause ≥ 5-fold increase in AUC or ≥ 80% decrease in clearance of sensitive CYP substrates
- moderate inhibitor implies that it can cause 2 to 5-fold increase in AUC values or 50-80% decrease in clearance of sensitive CYP substrates.
- a weak inhibitor is one that cause a > 1.25-fold but < 2-fold increase in the plasma AUC values or 20-50% decrease in clearance.
- distinction is not always categorical as interaction can vary according to conditions.
- macrolide antibiotics: azithromycin is not a CYP3A substrate. It may therefore be employed where antibiotic therapy with a macrolide is desirable in a patient being treated with Palbociclib
- statins: atorvastatin or pravastatin may be co-administered with Palbociclib, since a PK interaction study has shown that there is no relevant PK interaction.

9. Study procedure

Patient monitoring from the date of randomization till surgery and the patient's assessments' schedule are described in appendix 1.

9.1. Baseline assessment

Screening step:

Eligible patients who have provided the first written informed consent will undergo the following baseline assessments which should be completed within a period of 30 days prior to randomization.

However, to include a patient it will be possible to use some examination results performed before enrollment in the trial.

- **Clinical examination**

  Complete clinical examination with weight, height, medical history and associated treatment,

  ECOG performance Index (appendix 3),

- **Paraclinical examination:**

  Within 2 months prior randomization: bilateral mammography and Mammary Ultrasound.

  For cN0 patients, the axillary area must be evaluated with an ultrasonography. Any suspect lymph node should be sampled with US guided fine needle aspiration (No sentinel lymph node procedure should be performed at this stage).

  If clinically indicated, metastatic work up should include a thorax abdomen pelvis CT scan, or a PET scanner, or MRI according to local procedures.

  A baseline bone density is recommended but not mandatory for the trial,

- **Histopathological assessment of the primary tumor**

  Pathological tumor type and EE grade (**Scarff-Bloom & Richardson's** (**SBR**) classification as **modified** by **Elston** and **Ellis**),

  Measurement of estrogen and progesterone receptors (immunohistochemistry only):

  Estrogen receptor and progesterone receptor will be respectively considered as positive when ≥ 10% (Allred ≥4).

  Immunohistochemical determination of cerbB2 expression (FISH or CiSH if required).

  Ki67 staining

- **Mandatory FFPE block sample collection**

  Collection of Paraffin Tumor block(s) for PAM50 testing and ancillary studies.

The PAM 50 testing will be centralized at Institut Curie. Each patient will undergo 2 core biopsies, formol fixed and paraffin embedded (FFPE), of which one will be sent to Institut Curie Biopathology Department (Dr Anne Vincent-Salomon) along with the PAM50 screening form as described in section 14. The second core biopsie will be kept at the investigator site.

Randomization step:

Eligible patients who have provided the second written informed consent will undergo the following baseline assessments which should be completed within a period of 30 days prior to randomization.

- **Clinical examination**
  Complete clinical examination with weight, height, medical history and associated treatment,

  ECOG performance Index (appendix 3),

- **12 Lead ECG, LVEF**

- **Blood tests**
  Hematology (hemoglobin, WBC, neutrophils, lymphocytes and platelet count),

  Blood chemistry (Na, K, Chloride, Ca),

  Renal and Liver enzymes (creatinine, urea, bilirubin, ALT, AST, ALP, γGT),

  Ca15.3

- **Mandatory Blood sample:** 10 ml of blood to be collected into an EDTA tube.

After positive result of PAM50 testing and once all inclusion and exclusion criteria have been verified and all required baseline examinations have been performed and the second informed consent form is signed the participant will be included and randomized.

9.2. Assessments during treatment phase at day 1 of each cycle.

For the purpose of this trial one cycle = either 28 days for arm A (Letrozole + Palbociclib) or 21 days for arm B (chemotherapy).

- **Clinical examination**
  Complete clinical examination with weight and associated treatment,

  ECOG performance Index (appendix 3)

- **Investigations if clinically relevant**

- **Evaluation of treatment toxicity and adverse events**

- **12-Lead ECG** (note: In case of abnormal QT/QTc (>501 ms) a close ECG monitoring shall be adapted (continuous) in hospital until the advice of a cardiologist)

- **Blood tests** *(note: can be performed outside of the center)*
  Hematology (haemoglobin, neutrophils, lymphocytes and platelet count),

  Note: For patient enrolled in Arm A, hematology should be repeated at day 14 for cycle 1 and cycle 2.

  Blood chemistry (Na, K, Chloride, Ca),

  Renal and Liver enzymes (creatinine, urea, bilirubin, ALT, AST, ALP, γGT),

- **Paraclinical examination at week 8:** unilateral mammography and Mammary Ultrasound.

9.3. End of treatment evaluation

- **Clinical examination**
  Complete clinical examination with weight and associated treatment,

  ECOG performance Index (appendix 3)

- **Investigations if clinically relevant**

- **Evaluation of treatment toxicity and adverse events**

- **Blood tests** *(note: can be performed outside of the center)*
  Hematology (haemoglobin, neutrophils, lymphocytes and platelet count),

  Blood chemistry (Na, K, Chloride, Ca),

  Renal and Liver enzymes (creatinine, urea, bilirubin, ALT, AST, ALP, γGT)

- **Paraclinical examination:** unilateral mammography and Mammary Ultrasound.

- **12-Lead ECG**

9.4. Surgery

- **5 days before surgery (Arm A)**
  Only for the arm A, blood tests (Hematology) will be performed 5 days before the surgery. In case of Neutropenia grade > 2 or other toxicity grade > 2, Palbociclib will be permanently stopped and a control blood test will be performed the day prior to surgery.

- **Breast surgery**
  Breast surgery will be performed at week 20 +/- 1 week, i.e. 4 weeks after the end of chemotherapy (Arm B) and 1 day after the end of the palbociclib letrozole combination (Arm A)

Rationale for decision of either breast conserving surgery or mastectomy should be notified in the CRF.

Preoperative radiation therapy is forbidden unless the clinical course renders surgery not feasible. Pathological response will therefore be classified RCB III (see below §12.1).

Immediate reconstruction after mastectomy is allowed according to local guidelines.

- **Lymph node surgery**

  Lymph node surgery is considered at two steps of the study

  At inclusion, before chemotherapy of palbociclib-letrozole: Sentinel Lymph Node (SLN) sampling is not permitted as a method to assess axillary lymph node status

  At the end of the medical treatment: as local RCB is the primary endpoint, all patients should have an axillary surgery along with the breast surgical procedure. However, SLN procedure is allowed, and SLN results will be included in the RCB assessment (see below)

9.5. Pathology assessment

The Residual cancer burden (RCB) evaluation will be performed both locally and centrally. RCB is estimated from routine pathologic sections of the primary breast tumor site and the regional lymph nodes after the completion of neoadjuvant therapy and will be calculated as describe in section 14.1.2 and appendix 6.

9.6. Follow-up visits

Post neoadjuvant and post-surgery treatments are left to the investigators discretion but will be recorded. Patients who will have received palbociclib and letrozole and who are not RCB 0-I may be proposed chemotherapy, as per local guidelines.

Follow-up visits will be performed at **1, 6, 12, 18, 24, 30, 36 months after surgery**.

- **Clinical examination**

  Complete clinical examination with weight, height, medical history and associated treatment,

  ECOG performance Index (appendix 3),

- **Blood tests**

  Hematology (haemoglobin, neutrophils, lymphocytes and platelet count),

  Blood chemistry (Na, K, Chloride, Ca,),

  Renal and Liver enzymes (creatinine, urea, bilirubin, ALT, AST, ALP, γGT),

  CA15.3: Only if initially above upper normal limit

- **Paraclinical examination:**
  Yearly unilateral mammography

10. PREMATURE END OF THE TREATMENT

The treatment can be stopped early for the following reasons:

- toxicity (any clinical adverse event, laboratory abnormality or intercurrent illness which in the opinion of the investigator indicates that continued treatment with study therapy is not in the best interests of the patient,
- compulsory detention for treatment of either a psychiatric or physical (e.g., infectious disease) illness
- disease progression,
- patient's refusal to continue the treatment,
- consent withdrawal,
- patient lost to follow-up,
- major protocol violation
- termination of the study by the sponsor.

The same follow up requirements apply to patients for whom the treatment is prematurely ended within the limits of what can be reasonably achieved.

Any participant who stops prematurely the treatment due to an adverse event or a biological abnormality will be followed until the undesirable event is reversed and/or the biological test(s) has(ve) returned to normal. Participants who exit the trial prematurely will not be replaced.

As far as possible, the participants for whom the treatment was prematurely stopped will be followed up under the same standards as for the other participants.

The date and reason for premature trial exit or treatment stop will be reported in the case report file. In that case, a final evaluation will be performed and the results reported in the appropriate section of the case report file (page for study exit visit).

11. TRIAL TERMINATION CRITERIA

The trial can be suspended or stopped by the sponsor in consultation with the principal investigator at the request of the competent authority and/or the Ethic Committee for the following reasons:

- unexpected occurrence or severity of toxicity,
- insufficient patient recruitment,

- poor quality of data collection.

12. EVALUATION CRITERIA

12.1. Main criterion

The local Residual Cancer Burden (RCB) rates in the two arms of the study (in Luminal A N+ and Luminal B patients) is the main criterion of evaluation. Local RCB will be assessed right after surgery. The RCB method has been reported by Symmans et al in 2007 [25]. It has recently been updated and further validated [25]. The RCB is estimated from routine pathologic sections of the primary breast tumor site and the regional lymph nodes after the completion of neoadjuvant therapy. Six variables are included in a calculation formula.

The six variables are the following:

1. The primary tumor bed area in its two dimensions (i.e. 2 variables). For multifocal tumors, the largest one is considered.
2. The overall cancer cellularity (percentage of area)
3. The proportion of in situ disease (percentage of area)
3. The number of metastatic lymph nodes
4. The diameter of the largest lymph node metastase

The calculated RCB index value can also be categorized as one of four RCB classes. The calculation formula and detailed description can be found at a dedicated Web site: http://www.mdanderson.org/breastcancer_RCB. The four classes are the following:

1. RCB 0 : no residual disease
2. RCB-I (minimal RD), Minimal residual disease
3. RCB-II (moderate RD)
4. RCB-III (extensive RD).

The detailed procedures are provided in the section 14.1.2 and appendix 6.

For patients with inoperable or Progressive Disease: The RCB index cannot be accurately calculated for patients whose disease remains inoperable at the completion of the neoadjuvant treatment course (e.g., requiring subsequent additional treatments before surgical resection is possible), or those who experience disease progression and so do not undergo surgical resection at the completion of the neoadjuvant treatment course. **For those patients, RCB is assigned as extensive, i.e., RCB-III**.

12.2. Secondary criteria

12.2.1. Efficacy

In Luminal A N+ and Luminal B patients:

- Clinical response in each treatment arm as defined by clinical and US examination.
- Positive and negative predictive values of PAM50 ROR-defined status in both arms
- Rates of breast conservation therapy in the two arms, with regard to the initially planned surgery
- PFS is defined as the interval between the date of randomization and the date of tumor progression, relapse (local, regional or distant) or death from any cause, whichever occurs first.
- iDFS is defined as the interval between the date of randomization and the date of invasive breast cancer relapse (local, regional or distant) or the date of invasive contralateral breast cancer or second invasive cancer or death from any cause, whichever occurs first.
- Central RCB rates

In Luminal A N- patients:

- Clinical/radiological response rates (RECIST 1.1)
- Safety (CTC-AE v4.0)
- Central RCB rates
- Rates of BCS with regard to the initially planned surgery

12.2.2. Safety

In order to be considered as eligible for toxicity evaluation the patients must have received at least one dose of treatment after randomization.

The toxicity will be evaluated according to the scale: CTC-AE version 4.0 (see appendix 4).

12.2.3. Biology

A tumor collection will be constituted in order to assess several biomarkers as potential predictors of clinical and pathological response in both arms, such as p53 mutation status, p16/CDKN2A, RB1 and CCND1 status, as well as other proliferation and senescence biomarkers.

13. STATISTICS

13.1. Sample size determination and statistical analysis

A Fleming two-step statistical design will be used in the experimental arm:

- **Based on the RCB 0-I results with standard chemotherapy as administered in the standard arm, the null hypothesis (p0) will be that RCB 0-I is observed in 20% of the cases (p0=0.20)**
- **The alternative hypothesis will be p1 = 40% (0.40)**
- **Accepting a type I error of 0.045, and a type II error of 0.042 (power = 95.8%), the required number of patients is 60 evaluable patients per arm, therefore 132 patients will need to be included (estimation of 10% risk of non-evaluable patients). As about 10% of PAM50 evaluable patients will be classified as non luminal, and taking into account potential technical failures, about 180 patients will be screened.**

- **The first interim analysis will be planned after 30 patients** (luminal A N-positive and luminal B) **are available for local RCB evaluation in the experimental arm (step 1)**

At the end of step 1, among the first 30 patients included in the experimental arm

- o **If 5 or less than 5 local RCB 0-I are observed (16.7%), the trial will be stopped for futility**
- o **If 6 or more local RCB 0-I are observed, the trial will continue accrual**

After inclusion of 60 evaluable patients in both arms, final analyses will be conducted (step 2):

- o **If 17 or less than 17 local RCB 0-I are observed (28.3%) in the experimental arm, the trial's objective will be considered as not reached,**
- o **If 18 or more than 18 local RCB 0-I are observed (30%), a 20% RCB 0 – 1 rate can be rejected and the trial's objective will be considered as reached. Depending on the results obtained, a phase III trial will be then developed.**

The observed local RCB's rate in the control group will be checked at step 1 and 2 in order to verify the a priori hypothesis (20% RCB 0-1 in the control arm)

As the safety of the experimental strategy has been evaluated in the metastatic setting, no interruption in the accrual will be done between step 1 and 2, in order to maintain the dynamic of accrual in the trial

Luminal A N-negative patients will be analyzed independently as a prospective cohort.

2) Randomization will use block permutations technique and will be stratified based on :

- **T2 versus T3**
- **PAM 50 luminal A vs luminal B**

3) Analyses

Principal analyses will give the proportion of RCB 0-1 patients in each group, with their 95% confidence intervals.

Secondary endpoints will be assessed using classical statistical methods and results will be given with their 95% confidence intervals.

13.2. Definition of population

Intent-to-treat population

All randomized patients will be included in the intent-to-treat population (ITT), whether or not any study medication was administered after randomization, and regardless of the eligibility status. As far as statistical inferences are concerned, patients are analysed in the treatment group and in the stratum to which they were assigned by the randomization.

Per protocol population

Eligible patients are patients with no major violations of the inclusion and exclusion criteria.

Safety population

The safety population will consist of all treated patients, that is, all patients who received at least one dose of treatment after randomization, regardless of their eligibility for the study. Moreover, patients will be analysed according to the treatment regimen they actually received.

14. Pathology

The principal investigator of each site participating in the trial is responsible for informing the pathologist about the protocol and in particular about investigations and sample processing for PAM50 testing, central pathology review and translational research.

The principal investigator will further be responsible for ordering biopsy material from the pathologist and he/she is responsible for sending the tumor tissue/biopsy to the central review pathologist.

Logistic flow for pathological samples is summarized as a scheme in appendix 7.

14.1. Task for the local pathologist

14.1.1. Tumor material for central PAM50 testing

2 core biopsies have to be done, formalin fixed and processed as 2 distinct paraffin embedded blocks (FFPE).

The task for local pathologist is to send one core biopsy to Institut Curie, Biopathology Department (Dr Anne Vincent-Salomon) (see section 14.1.4) along with the pathology form.

Specific pathological data have to be recorded on the website (type, grade, ER, PR, Her2, Ki67, cellularity). The link to the website will be given during the site initiation visit

The second core biopsy will be stored at the investigator site.

The tumor biopsy to be sent should have been tested ER-positive (>10%) and HER2 negative by local pathology assessment.

14.1.2. RCB determination

Detailed procedures are provided in appendix 6.

Primary Tumor Bed

1. GROSS. Identify the probable tumor bed and describe this macroscopic finding:

a. Report the measurements of the largest gross dimensions (prefer three dimensions, but minimum is two dimensions).

b. Submit the largest cross-sectional area for histology and specifically describe those blocks in the Section Code:

i. Try to indicate how they are oriented by photography, radiography, photocopy, or intelligent description (e.g., "sections B1 – B7 cross section of tumor bed in rows from antero-superior to postero-inferior").
ii. If additional sections are from surrounding tissues, then describe those as well.
iii. Five representative sections from a big, obvious tumor bed should be sufficient.

2. MICROSCOPY. Review the slides that correspond to the tumor bed (+/- surrounding tissues):

a. Estimate the extent of spread of residual cancer relative to the gross tumor bed:

i. If similar to the gross description, then keep the original measurements.

ii. If obviously different, then revise the dimensions of the tumor bed based on the microscopic review of the tumor bed.

iii. Suggestion: Dotting the perimeter of cancer in each slide can be helpful to reconstruct the tumor extent across multiple slides (see point 1-b-i).

b. Using the microscope, make visual snapshots of cancer cellularity as you go from field to field across the defined tumor bed from one end to the opposite (e.g., left to right, then top to bottom) to estimate the:

i. Average cancer cellularity (%) across the entire tumor bed. **This is all cancer, whether invasive or in situ**.

ii. Average percent of the cancer within the tumor bed that is in situ.

iv. Cellularity estimates are to the nearest 10%, with additional selections of 1% and 5% for very low cellularity. For reference, there are images of computer generated examples linked to our Web site: http://www.mdanderson.org/breastcancer_RCB.

v. The usual misunderstanding is to only make estimates in foci of the tumor bed that contain lots of cancer. The estimates are supposed to represent the average across the entire residual tumor bed area

Regional Lymph Nodes: Pathologic evaluation of the primary tumor bed in the breast requires that the pathologist make two judgments:

i. Count the number of positive lymph nodes.

ii. Measure the diameter of the largest nodal metastasis.

14.1.3. Surgery tumor material for central review

The local pathologist will select the most representative block from the residual tumor together with the largest axillary Lymph node metastasis if present.

The local pathologist will send the biological material to Institut Curie, Biopathology Department (Dr Anne Vincent-Salomon) (see section 14.1.3)

14.1.4. Labelling and shipment of tumor material

All material has to be clearly labelled with local number of paraffin block and the trial identification 'NeoPAL'

Material has to be sent along with the biological material transfert form duly completed.

Shipping address:

Dr Anne Vincent-Salomon

NeoPAL Study

Institut Curie -Departement de Biopathologie

26 rue d'Ulm - 75005 Paris - France

Shipment has to be organized by the investigator site.

14.2. Task for the central pathologist

14.2.1. PAM 50 testing

The task for the central pathologist is to perform from the core biopsy received:

- One H&E tissue section for cellularity evaluation and for delineation of the highest tumor cellular area.

- Five 10 µm tissue sections

Macrodissection will then be performed in the Pharmacogenomics unit of the Biopathology Department at Institut Curie (Dr Ivan Bièche) for RNA extraction.

Quantity and Quality control will be performed, and 125 ng RNA will be sent to Institut Curie Nanostring nCounter platform (David Gentien).

PAM 50 test results will be controlled by both the Pharmacogenomic unit of the Biopathology Department at Institut Curie (Ivan Bièche) and the Nanostring platforms, and the following results will be disclosed to the patient and the investigator through a web interface

- Subtype classification : luminal A, luminal B, non luminal. Patients with a HER2 enriched subtype will not enter the study, and HER2 status evaluation on the primary tumor is to be checked by the referring investigator.
- ROR-S classification

The remaining FFPE biopsy will be banked to UNICANCER Biobank (CRB - Centre Léon Bérard, Lyon) for translational research.

14.2.2. RNA and DNA collection

Collection of remaining RNA (after PAM 50 testing) will be constituted for ancillary studies (additional biomarkers using RT-PCR) and will be sent to the UNICANCER Biobank (CRB - Centre Léon Bérard, Lyon).

DNA collection will be processed from blood samples or tumor blocks with enough remaining tumor tissue for ancillary studies.

15. SAFETY

15.1. Adverse Event

15.1.1. General definition

An adverse event (AE) is defined as any untoward/unfavorable medical occurrence, related or not to the research or one of the investigational products, in an individual who participates in a biomedical research.

15.1.2. Known undesirable effects of Palbociclib

AEs most frequently (≥ 20%) observed with palbociclib that were considered to be related to the study treatment were neutropenia, fatigue, diarrhea and anaemia.

Please refer to the Investigator Brochure for more detailed information.

15.2. Serious Adverse Event

15.2.1. General definition

A serious adverse event (SAE) is defined as any untoward medical occurrence or effect that at any dose:

- results in death,
- is life-threatening,
- requires hospitalization or prolongation of existing hospitalization,
- results in persistent or significant disability or incapacity
- induces a congenital anomaly or birth defect
- is medically relevant

Medical and scientific judgment should be exercised in deciding whether other situations should be considered serious, such as important medical events that might not be immediately life-threatening or result in death or hospitalization, but might jeopardize the patient or might require intervention to prevent one of the other outcomes listed in the definition above (for example : overdose, second cancer, …).

These characteristics/consequences have to be considered at the time of the event. For example, regarding a life-threatening event, this refers to an event in which the subject was at risk of death at the time of the event; it does not refer to an event which hypothetically might have caused death if it was more severe.

The terms disability and incapacity correspond to any clinically relevant physical or psychic handicap, transient or permanent, with impacts on the physical condition/activity and/or the quality of life of the patient.

The following are not considered to be serious adverse events (SAE):

- Hospitalization for care, procedures or all investigation performed on an outpatient basis without any seriousness criteria associated,
- Hospitalization already scheduled before the start of the trial and/or which is part of the protocol (biopsy, chemotherapy, etc),
- Hospitalization occurring in the context of tumor progression of disease under study,
- Progression of disease under study**,
- Clinical events related to progression of disease under study are not to be reported as SAEs,
  **Only progression leading to death and when the death occurs within 30 days after the last administration of study treatment must be reported.

15.2.2. Definition of Suspected Unexpected Serious Adverse Reaction (SUSAR)

A serious adverse reaction, the nature or the severity of which is not consistent with the applicable product information (e.g., Investigator's Brochure for an unapproved investigational product or package insert/summary of product characteristics for an approved product).

When the outcome of the serious adverse reaction is not consistent with the applicable product information this adverse reaction should be considered as unexpected.

The reference document to assess expectedness for the different study drugs is either IB for Palbociclib or SPC for other drugs.

The expectedness assessment is the responsibility of the Sponsor.

15.2.3. Severity criterion

The criterion for severity (intensity) must not be confused with the seriousness criterion which is the guide for defining the reporting requirements.

The term "severity" is used here to describe the intensity of a specific event.

The severity of events will be estimated according to the extract from the CTC-AE version 4.0 classification (Appendix 4). The severity of adverse events not listed in this classification will be assessed according to the following qualifiers:

- Grade 1: Mild; asymptomatic or mild symptoms; clinical or diagnostic observations only; intervention not indicated.
- Grade 2: Moderate; minimal, local or noninvasive intervention indicated; limiting age-appropriate instrumental ADL.
- Grade 3: Severe or medically significant but not immediately life-threatening; hospitalization or prolongation of hospitalization indicated; disabling; limiting self-care ADL.
- Grade 4: Life-threatening consequences; urgent intervention indicated.
- Grade 5: Death related to AE.

15.2.4. Handling of a serious adverse event

The investigator must immediately and no later than 24 hours following knowledge inform, the R&D UNICANCER Safety Office of any SAE,

From the signature of the first informed consent form, only SAE related to the biopsy performed for the protocol must be reported.

From the signature of the second informed consent form, until 60 days following the surgery, any SAE whether or not related to the research must be reported.

Any delayed SAE, i.e. occurring after a period of 60 days, which are considered to be related to the study treatments or to the research (other treatments used, diagnostic procedures and examinations carried out during the research) must be reported without any limitation in terms of deadline.

Abnormal laboratory results should be reported as SAE if they possibly put at risk the patient or they require medical intervention to prevent an outcome corresponding to one of severity criteria.

Second cancer, whether or not related to the research, must be reported to the R&D UNICANCER Safety Office without any limitation in terms of deadline.

Notification must be carried out immediately and no later than 24 hours following knowledge, by fax to the R&D UNICANCER Safety Office by sending the form entitled "notification of a serious adverse event", located in the investigator file, completed as precisely as possible, dated and signed by the investigator:

R&D UNICANCER
Safety Office, France
Tel.: +33.(0)1 44 23 04 16 – Fax: +33.(0)1 44 23 55 70

Email: pv-rd@unicancer.fr

The investigator should also attach to the form "notification of a serious adverse event", whenever possible:

- A copy of the hospital or extended hospitalization report,
- A copy of the autopsy report if necessary,
- A copy of all results of additional investigations carried out, including relevant negative results, and enclosing the normal laboratory values,
- Any other document which he believes to be useful and pertinent.

All these documents must be anonymised.

Additional information can be requested (by fax, mail, telephone or visit) by the monitor and/or the R&D UNICANCER Safety Office.

The investigator is responsible for appropriate medical follow-up of patients until the resolution or stabilization of the event or until the death of the patient. This can sometimes mean that the follow-up continues after the patient has left the trial.

The investigator shall send additional information to the R&D UNICANCER Safety Office using an SAE declaration form (by ticking the Follow-up no X box to specify that it is a follow-up and not an initial report) as soon as he is aware of the event. He shall also submit the last follow-up at the resolution or stabilization of the SAE.

The investigator must keep the documents concerning the suspected serious adverse event in order to supplement the information previously submitted if necessary.

16. STEERING COMMITTEE AND INDEPENDENT DATA MONITORING COMMITTEE

16.1. Trial Steering Committee

A steering committee made up of the principal investigators, the UNICANCER project manager, the study statistician and some investigators involved in the study will be established to ensure the protection of patients, to ensure that the trial is conducted in an ethical manner, to assess the risk / benefit ratio of the test and ensure the review of current scientific results or the end of the trial. All participating groups will be represented in the steering committee.

16.2. Independent Data Monitoring Committee (IDMC)

A trial monitoring committee (IDMC = Independent Data Monitoring Committee) may be set up to ensure: (1) protection of the patients; (2) that the trial is conducted according to the ethics; (3) that the scientific results are reviewed independently during the course and at the end of the trial; and (4) to evaluate the benefit/risk ratio for the trial. This committee will be formed during the study in order to ensure an independent review of data and to ensure the protection of patients

This committee has a consultative role with respect to the sponsor. In addition to the two planned interim analysis (see section 11.1) the IDMC will review frequently the safety data and will be in charge to make recommendations to sponsor.

Data presented to IDMC are strictly confidential.

The sponsor takes the final decision regarding the recommendation proposed by the committee.

17. QUALITY INSURANCE AND QUALITY CONTROL

In order to guaranty the authenticity and credibility of the data in accordance with the Good Clinical Practices, the sponsor will set up an assurance quality program that includes:

- Management of the trial according to trial specific procedures provided by UNICANCER.
- control of the quality of the data provided by the investigation site is performed by the study monitor the role of which is to match and check the consistency of the data reported in the observation handbook with respect to the source-documents,
- possible audit of investigational sites,
- conducting a centralized review covering certain aspects of the protocol (to be specified).

18. DATA PROTECTION AND CONFIDENTIALITY MANAGEMENT

Until the trial results are published, the investigator is responsible for insuring the confidentiality of the totality of the information, handled by herself/himself and all other individuals involved in the course of the trial, that are supplied by UNICANCER. This obligation holds neither for the information that the investigator may communicate to the patients within the context of the trial nor for the already published information.

The investigator commits not to publish, not to spread or use in any manner, directly or indirectly, the scientific and technical information related to the trial.

Nevertheless, both the center and the investigator may communicate information relative to the trial:

- to the Health Minister,
- to the public health inspectors who are doctors,
- to the public health inspectors who are pharmacists,
- to the Competent Authority General Director and inspectors.

The trial will not be the subject of any written note and/or oral comment without the prior agreement of the sponsor; the totality of the information that is communicated or obtained during the course of the trial belongs in full right to the French Federation of Comprehensive Cancer Centers who can freely use it.

19. PUBLICATION GUIDELINES

All information resulting from this trial is considered to be confidential, at least until appropriate analysis and checking has been completed by the sponsor, the principal investigator and the statistician of the trial.

Any publication, abstract or presentation comprising results from the trial must be submitted for examination and approval to the Sponsor (UNICANCER).
Furthermore, any written communication or presentation must imperatively include a section that mention UNICANCER, La Ligue Nationale Contre le Cancer (The French League for Treating Cancer) and any institution, investigator, cooperating or collaborating group and scientific society that has contributed to the trial as well as any organism that has financially supported this research (for example Cancer Research UK).

19.1. Primary manuscript and presentation

The first author of the publication will be the trial chairperson. She/he may however designate another person to (co-) write the publication. In case 2 trial chairpersons are involved for the same protocol, one of them will be first author and the second will either be co-first author or at last position for the primary publication. The second chairperson will present the trial during its first presentation during an international meeting.

The other investigators will appear in the list of co-authors in decreasing order, according to the number of recruited patients. Each cooperating group shall be represented in proportion to their level of patient accrual. Each cooperating group having enrolled more than 5% of evaluable cases deserves a position in the author list. The trial steering committee may adapt this threshold depending on the number of contributing group and the length of the author list accepted by the target journal. Each member of the steering committee who has included more than 10 patients will be an author. In addition, the statistician and at least one representative of UNICANCER will be cited as well.

19.2. Secondary manuscripts

The first author of the publication will be determined according to the number of recruited patients and should be approved by the trial steering committee. Authorship should be rotated across publications.

19.3. Sub-studies manuscripts

In an equal manner, the first author of the publication of the sub-studies (biological studies) will be the person who has carried out the sub-studies. All the individuals who have taken part in carrying out these sub-studies shall be represented. At least the trial chairperson(s) and further key clinical persons should be coauthors. Moreover, at least one representative of UNICANCER will be cited as well.

20. Translational research

To enable the development of translational research projects, a collection of blood, FFPE core biopsy and tumor block from the primary tumor is planned.
The consent of the patient has already been incorporated on a separate form. The patient's refusal to participate in collection of tumor tissue will not be an exclusion criterion for the clinical trial.

These samples will be stored in France, and Belgium and will be made available in accordance with the recommendations and approval of the translational research committee of this trial.

Proposals for additional translational analyses and projects, not specified in this protocol, will be subject to approval by UNICANCER according to UNICANCER standard operating procedures (Charter of translational research studies).

21. Database access

Study data will be made available to access requests after publication of the primary objective of the trial.

Data access requests for any proposed sub-study will be subject to review by the VALPACS Committee of UNICANCER.

Researchers will need to request a formal data extraction by submitting a complete project application to UNICANCER.

On approval of a successful application, the UNICANCER data-management center will provide a data extraction tailored to the specific needs of the research study.

22. ETHICAL AND REGULATORY ISSUES

The clinical trial must be conducted in accordance with:

- the principles of ethics as stated in the last version in use of the Declaration of Helsinki,

- the Good Clinical Practices defined by the International Conference on Harmonization (ICH–E6, 17/07/96),

- the European directive 95/46/CE on the protection of individuals with regard to the processing of personal data and on the free movement of such data,

- the European directive 2001/20/CE on the conduct of clinical trials.

22.1. General Requirements

The study will be performed according to this study protocol, the Declaration of Helsinki, the guidelines of ICH-GCP and the respective national legal requirements. Special emphasis will be placed on data protection. As this clinical study will be carried out in Europe the study will be conducted according to the EU directive on data protection (95/46/EC).

The sponsor is obliged to obtain evidence of the investigator's qualification to perform the clinical study. Therefore, the investigator has to provide a dated and signed copy of his professional curriculum vitae (no older than two years and preferably one page-length in English) prior to the start of the clinical study, including information about his experience in conducting clinical studies according to the guidelines for GCP.

22.2. Independent Ethics Committee (IEC) or Institutional Review Board (IRB)

Prior to the start of the study, the sponsor or investigator will submit the study protocol, patient information, informed consent(s), and other study-related documents, as required by local regulations, to the respective regulatory authorities and the responsible IEC/IRB for their written approval.

The sponsor or investigator will inform the IEC/IRB and regulatory authorities, according to local regulations, about protocol amendments including any new information that require an ethical reconsideration of the study protocol.

In addition to the written approval of the IEC/IRB, the sponsor or investigator should obtain either the list of IEC/IRB members or a statement from the IEC/IRB that the institution is composed and organized according to, and adheres to GCP and applicable regulations.

As required by local regulation, by the IEC/IRB, or regulatory authorities, the sponsor or investigator will also submit the financial arrangements for the study or other financial interests of the investigator in the IMP or sponsor company to the IEC/IRB and regulatory authorities.

Furthermore, if required by local regulation, the sponsor or investigator will submit a summary of the clinical study results to the IEC/IRB.

Unless otherwise instructed by the IEC/IRB or by local regulation, the sponsor or the investigator must submit to the IRB/IEC:

- Information on adverse events that are serious AND unexpected AND associated with the IMP from the investigator's site, as soon as possible
- Expedited safety reports from the sponsor, as soon as possible
- Periodic reports on the progress of the study

22.3. Competent Authority

Before the start of the trial, the authorization by competent authority must be obtained. The submission must be done according to the European directive (2001/20/CE) and national regulation.

22.4. Patient Information and the informed consent of participants

The patient information sheet and consent form must be written in conformity with the Good Clinical Practices defined by the International Conference on Harmonization (ICH).

Prior to carrying out biomedical research on human patients, an informed consent form must be signed, without coercion, by each individual participating in the trial after she/he has been informed by the investigator during a physician-patient consultation and after sufficient time for reflection has been allowed.

The information sheets and informed consent forms must be associated within the same document to insure that all the information regarding the trial is transferred to the participant.

The consent forms must be dated and signed by both the participant in the research and the investigator. The original document is archived by the investigator; a copy is given to the research participant.

If genomic or proteomic analysis is one of the trial's objective, the information sheet must specify the type of research that will be performed and the patient must have the possibility to accept or refuse that the biological samples taken from her/him will be conserved for the purpose of scientific research.

22.5. Sponsor responsabilities

UNICANCER, the sponsor of the trial, has initiated this biomedical research on human subjects and is therefore accountable for the research management and for verifying that the financing schedule covers the anticipated expenses.

The main sponsor responsibilities are:

- to write the research protocol and amendments, as well as all other documents attached to it and/or required for the study.
- to subscribe a civil-responsibility insurance,
- to obtain an EudraCT (European Drug Regulatory Authorities Clinical Trials) identification number,
- to request the opinion of the Independent Ethic Committee (IEC) and authorization from Competant authority on the initial project and possible amendments,
- to declare to the competent authority any suspected unexpected serious adverse reactions (SUSAR) and communicate the information to the EC and investigators of the trial,
- to file annually the security report to the competent authority and the EC,
- to communicate the information on the trial to the investigation centers' heads, pharmacists and physician-investigators,
- to declare the beginning and end of the trial to the competent authority,
- to write the final report on the trial and communicate it to Competent authority,
- to communicate the information on the trial's results to the competent authority, the EC. The result may also be communicated to the research participant, at their request, by the physician-investigator.
- to archive the trial's essential documents for a minimal duration of 15 years after the research has ended.

22.6. Investigator Responsibilities

The principal investigator of each health care center participating in the study commits to conducting the clinical trial in compliance with the study protocol and the regulations in force, notably according to the Good Clinical Practices, as defined by the International Conference on Harmonization (ICH–E6, 17/07/96).

<u>It is the responsibility of the principal investigator</u>:

- to provide the sponsor (or sponsor representative) with its own curriculum vitae and co-investigators' curriculum vitas. CVs must be dated and signed, and the investigator's reference number in the official Practitioners' Registry must be indicated

- to identify the members of his/her team who participate in the trial and to define their individual responsibilities,

- to start recruiting patients after the sponsor has issued its authorization,

- to be available for monitoring visits and investigator meetings.

It is the responsibility of each investigator:

- to ensure the confidentiality of all data recorded during the trial

- to collect the informed consent form, dated and signed personally by each individual research participant before any selection procedure specific to the trial may start,

- to regularly complete the case report form (CRF) for each patient included in the trial in a timely manner and to allow the clinical research assistant mandated by the sponsor to have direct access to the source-documents in order to validate the data collected in the observation CRF,- to declare to the sponsor any SAE occurring during the research, immediately upon being made aware of these,

- to accommodate regular visits by the study monitor and possibly auditors as mandated by the sponsor or the inspectors of the competent legal authorities,

- to date, correct and sign the corrections made in the CRF for each patient included in the trial,

All documentation relative to the trial (protocol, consent forms, CRF, investigators' folders, etc…) as well as all other original documents (laboratory results, radiology images, reports of physician-patient consultations and clinical examinations, etc…) are confidential material and must be kept in a secured location. The principal investigator will be required to store the data and a list of patient identifications (i.e. list of all enrolled patients) during a minimal period of 15 years after the study has ended.

22.7. Federation of the Patient Committees for Clinical Research in Oncology

The dedicated task of the Committee is to review clinical trial protocols in oncology. The French patient committees' federation is coordinated by the Ligue National and The French NCI (INCA), a body department of UNICANCER. The Committee reviews the trial protocol and documentation to suggest improvements in the quality of information given to the patients, the delivery of study treatment and monitoring plan, to maximize the convenience and comfort of the patients.

23. Bibliography

1. Siegel R, Ma J, Zou Z, et al: Cancer statistics, 2014. CA Cancer J Clin 64:9-29, 2014

2. Houssami N, MacAskill P, Von Minckwitz G, et al: Meta-analysis of the association of breast cancer subtype and pathologic complete response to neoadjuvant chemotherapy. European Journal of Cancer 48:3342-3354, 2012

3. Denkert C, Loibl S, MÃ¼ller BM, et al: Ki67 levels as predictive and prognostic parameters in pretherapeutic breast cancer core biopsies: A translational investigation in the neoadjuvant gepartrio trial. Annals of Oncology 24:2786-2793, 2013

4. Cortazar P, Zhang L, Untch M, et al: Pathological complete response and long-term clinical benefit in breast cancer: the CTNeoBC pooled analysis. Lancet, 2014

5. Lerebours F, Bourgier C, Alran S, et al: A randomized phase II neoadjuvant trial evaluating anastrozole and fulvestrant efficiency for post-menopausal ER-positive, HER2-negative Breast Cancer patients: first results of the UNICANCER CARMINA 02 French trial -- Lerebours et al. 72 (1024): PD07-04, Cancer Research December 17, 2012 72:PD07-04, 2012

6. Semiglazov VF, Semiglazov VV, Dashyan GA, et al: Phase 2 randomized trial of primary endocrine therapy versus chemotherapy in postmenopausal patients with estrogen receptor-positive breast cancer. Cancer 110:244-54, 2007

7. Torrisi R, Bagnardi V, Pruneri G, et al: Antitumour and biological effects of letrozole and GnRH analogue as primary therapy in premenopausal women with ER and PgR positive locally advanced operable breast cancer. British journal of cancer 97:802-8, 2007

8. C. Palmieri • S. Cleator • L. S. Kilburn • S. B. Kim • S.-H. Ahn • M. Beresford • G. Gong • J. Mansi • E. Mallon • S. Reed • K. Mousa • L. Fallowfield • M. Cheang • J. Morden • K. Page • D. S. Guttery • B. Rghebi • L. Primrose • J. A. Shaw • A. M. Thompson • J. M. Bliss • R. C. Coombes: NEOCENT: a randomised feasibility and translational study comparing neoadjuvant endocrine therapy with chemotherapy in ER-rich postmenopausal primary breast cancer. Breast Cancer Res Treat 2014:148; 581-90).

9. Ellis MJ, Tao Y, Luo J, et al: Outcome prediction for estrogen receptor-positive breast cancer based on postneoadjuvant endocrine therapy tumor characteristics. Journal of the National Cancer Institute 100:1380-1388, 2008

10. Freedman OC, Amir E, Hanna W, et al: A randomized trial exploring the biomarker effects of neoadjuvant sequential treatment with exemestane and anastrozole in post-menopausal women with hormone receptor-positive breast cancer. Breast cancer research and treatment 119:155-61, 2010

11. Smith IE, Dowsett M, Ebbs SR, et al: Neoadjuvant treatment of postmenopausal breast cancer with anastrozole, tamoxifen, or both in combination: the Immediate Preoperative Anastrozole, Tamoxifen, or Combined with Tamoxifen (IMPACT) multicenter double-blind randomized trial. Journal of clinical oncology : official journal of the American Society of Clinical Oncology 23:5108-16, 2005

12. Ellis MJ, Suman VJ, Hoog J, et al: Randomized phase II neoadjuvant comparison between letrozole, anastrozole, and exemestane for postmenopausal women with estrogen receptor-rich stage 2 to 3 breast cancer: clinical and biomarker outcomes and predictive value of the baseline PAM50-based int. Journal of clinical oncology : official journal of the American Society of Clinical Oncology 29:2342-9, 2011

13. Sotiriou C, Pusztai L: Gene-expression signatures in breast cancer. The New England journal of medicine 360:790-800, 2009

14. Dubsky P, Brase JC, Jakesz R, et al: The EndoPredict score provides prognostic information on late distant metastases in ER+/HER2- breast cancer patients. British journal of cancer 109:2959-64, 2013

15. Filipits M, Nielsen TO, Rudas M, et al: The PAM50 risk-of-recurrence score predicts risk for late distant recurrence after endocrine therapy in postmenopausal women with endocrine-responsive early breast cancer. Clinical cancer research : an official journal of the American Association for Cancer Research 20:1298-305, 2014

16. Gnant M, Filipits M, Greil R, et al: Predicting distant recurrence in receptor-positive breast cancer patients with limited clinicopathological risk: using the PAM50 Risk of Recurrence score in 1478 postmenopausal patients of the ABCSG-8 trial treated with adjuvant endocrine therapy alone. Annals of oncology : official journal of the European Society for Medical Oncology / ESMO 25:339-45, 2014

17. Sestak I, Dowsett M, Zabaglo L, et al: Factors predicting late recurrence for estrogen receptor-positive breast cancer. Journal of the National Cancer Institute 105:1504-11, 2013

18. Nielsen TO, Parker JS, Leung S, et al: A comparison of PAM50 intrinsic subtyping with immunohistochemistry and clinical prognostic factors in tamoxifen-treated estrogen receptor-positive breast cancer. Clinical cancer research : an official journal of the American Association for Cancer Research 16:5222-32, 2010

19. Cheang MCU, Voduc KD, Tu D, et al: Responsiveness of intrinsic subtypes to adjuvant anthracycline substitution in the NCIC.CTG MA.5 randomized trial. Clinical cancer research : an official journal of the American Association for Cancer Research 18:2402-12, 2012

20. Esserman LJ, Berry DA, Cheang MCU, et al: Chemotherapy response and recurrence-free survival in neoadjuvant breast cancer depends on biomarker profiles: results from the I-SPY 1 TRIAL (CALGB 150007/150012; ACRIN 6657). Breast cancer research and treatment 132:1049-62, 2012

21. Parker JS, Mullins M, Cheang MCU, et al: Supervised risk predictor of breast cancer based on intrinsic subtypes. Journal of clinical oncology : official journal of the American Society of Clinical Oncology 27:1160-7, 2009

22. Patani N, Martin LA: Understanding response and resistance to oestrogen deprivation in ER-positive breast cancer. Molecular and cellular endocrinology 382:683-94, 2014

23. Musgrove EA, Caldon CE, Barraclough J, et al: Cyclin D as a therapeutic target in cancer. Nature reviews. Cancer 11:558-72, 2011

24. Finn RS, John P. Crown, Istvan Lang, et al: Final results of a randomized Phase II study of PD 0332991, a cyclin-dependent kinase (CDK)-4/6 inhibitor, in combination with letrozole vs letrozole alone for first-line treatment of ER+/HER2- advanced breast cancer (PALOMA-1; TRIO-18), AACR 2014, 2014

25. Symmans WF, Peintinger F, Hatzis C, et al: Measurement of residual breast cancer burden to predict survival after neoadjuvant chemotherapy. Journal of clinical oncology : official journal of the American Society of Clinical Oncology 25:4414-4422, 2007

26. Symmans WF, Wei C, Gould R, et al: Long-term prognostic value of residual cancer burden (RCB) classification following neoadjuvant chemotherapy. Cancer Research 73:S6-02-S6-02, 2013

27. Ocana A, Amir E, Vera-Badillo F, et al: Phase III trials of targeted anticancer therapies: redesigning the concept. Clinical cancer research : an official journal of the American Association for Cancer Research 19:4931-40, 2013

28. Smith TJ, Khatcheressian J, Lyman GH, et al: 2006 update of recommendations for the use of white blood cell growth factors: an evidence-based clinical practice guideline. Journal of clinical oncology : official journal of the American Society of Clinical Oncology 24:3187-3205, 2006

Appendix 1a – Investigation Summary Table – Arm A (Letrozole + Palbociclib)

Visits	Selection	Inclusion	Cycle 1 to 4[a]	pre-operatory assessment (end of treatment)	Surgery	Assessment 1 month after surgery	Follow-up (6, 12, 18, 24, 30, 36 months after surgery)
Written Informed Consent	X	X					
Verification of eligibility criteria		X					
Medical history	X						
Biopsy collection and sending	X[b]						
Intratumoral coil placement	X						
PAM50 test result		X					
Clinical examination							
Clinical examination and vital signs	X	X	X	X		X	X
ECOG PS	X	X	X	X		X	X
Safety assessment			X	X			
Paraclinical examination							
Mammogram	X[c]		At week 8	X[c]			X[c]
Mammary Ultrasound	X		At week 8	X			
Lymph node ultrasound + fine-needle aspiration in case of suspect lymph node	X[g]						
MRI (optional)	X		At week 8	X			
PET-Scan (optional)	X						
ECG		X	X[f]	X[f]			
LVEF		X					
Biological examination							
Hematology, Blood Chemistry		X	X[i]	X[h]		X	X
Renal, Liver evaluation		X	X	X		X	X
CA 15.3		X				X[e]	X[e]
Tissue and Blood sampling							
Surgical tissue collection (initial FFPE slides + 1 block)					X		
Blood sample		X					
Treatment – ARM A							

**Letrozole 2.5 mg q1d**<br>**Palbociclib 125 mg q1d 3w/4**			**X[d]**				

a. 1 cycle = 28 days for Arm A (Palbociclib + Letrozole).
b. 1 FFPE fixed tumoral block sample to be sent for PAM50 testing.
c. Mammogram will be bilateral at inclusion and unilateral thereafter. During follow-up a yearly mammography is requested according to the standard of care.
d. Palbociclib and Letrozole will be pursed until the day before surgery. Palbociclib could be stopped 5 days before the surgery in case of Neutropenia.
e. Only if initially above upper normal limit.
f. In case of abnormal QT/QTc (>501 ms) a close ECG monitoring shall be adapted (continuous) in hospital until the advice of a cardiologist.
g. For cN0 patients, axillary area must be evaluated with an ultrasonography. Any suspect lymph node should be sampled with US guided fine needle aspiration (no sentinel lymph node procedure should be performed at this stage).
h. Hematologic examination will be performed 5 days prior to surgery.
i. Note: hematology should be repeated at day 14 for cycle 1 and cycle 2.

Appendix 1b – Investigation Summary Table – Arm B (Chemotherapy: 3 FEC 100 + 3 Doc 100).

**Visits**	**Selection**	**Inclusion**	**Cycle 1 to 6[a]**	**pre-operatory assessment (end of treatment)**	**Surgery**	**Assessment 1 month after surgery**	**Follow-up (6, 12, 18, 24, 30, 36 months)**
Written Informed Consent	**X**	**X**					
Verification of eligibility criteria		**X**					
Medical history	**X**						
Biopsy collection and sending	**X[b]**						
Intratumoral coil placement	**X**						
PAM50 test result		**X**					
Clinical examination							
Clinical examination and vital signs	**X**	**X**	**X**	**X**		**X**	**X**
ECOG PS	**X**	**X**	**X**	**X**		**X**	**X**
Safety assessment			**X**	**X**			
Paraclinical examination							
Mammogram	**X[c]**		**At week 8**	**X[c]**			**X[c]**
Mammary Ultrasound	**X**		**At week 8**	**X**			
Lymph node ultrasound + fine-needle aspiration in case of suspect lymph node	**X[e]**						
MRI (optional)	**X**		**At week 8**	**X**			
PET-Scan (optional)	**X**						
ECG		**X**	**X**	**X**			
LVEF		**X**					

Biological examination							
Hematology, Blood Chemistry		**X**	**X**	**X**		**X**	**X**
Renal, Liver evaluation		**X**	**X**	**X**		**X**	**X**
CA 15.3		**X**				**X**[d]	**X**[d]
Tissue and Blood sampling							
Surgical tissue collection (initial FFPE slides + 1 block)					**X**		
Blood sample		**X**					
Treatment – ARM B							
**FEC 100 x 3 followed by docetaxel 100 x 3**			**X**				

a. 1 cycle = 21 days for Arm B (Chemotherapy).
b. 1 FFPE fixed tumoral block sample to be sent for PAM50 testing.
c. Mammogram will be bilateral at inclusion and unilateral thereafter.
d. Only if initially above upper normal limit.
e. For cN0 patients, axillary area must be evaluated with an ultrasonography. Any suspect lymph node should be sampled with US guided fine needle aspiration (no sentinel lymph node procedure should be performed at this stage).

Appendix 2 – Treatment schedule

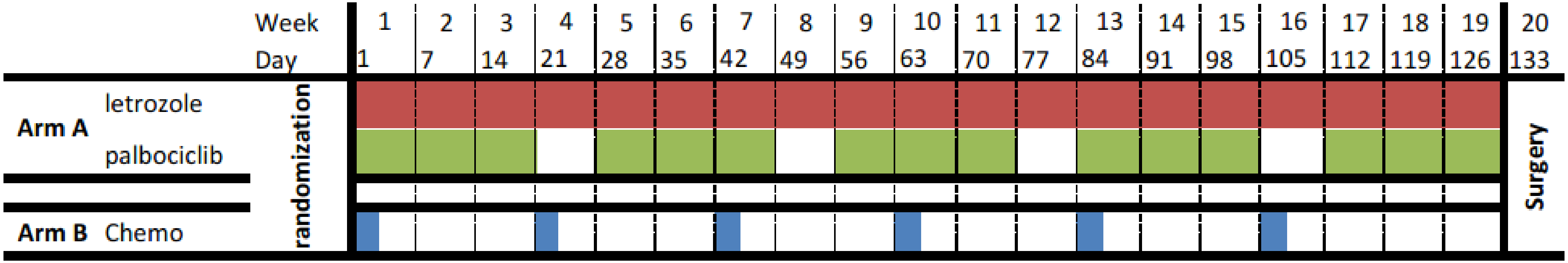

Appendix 3 – ECOG peformance status scale

ECOG PERFORMANCE STATUS	
0	Fully active, able to carry on all pre-disease performance without restriction
1	Restricted in physically strenuous activity but ambulatory and able to carry out work of a light or sedentary nature, e.g., light house work, office work
2	Ambulatory and capable of all selfcare but unable to carry out any work activities. Up and about more than 50% of waking hours
3	Capable of only limited selfcare, confined to bed or chair more than 50% of waking hours
4	Completely disabled. Cannot carry on any selfcare. Totally confined to bed or chair

Appendix 4 – Toxicity criteria (CTCAE)

http://ctep.cancer.gov/

Common Terminology Criteria for Adverse Events v4.0 (CTCAE)

(Publish Date October 1, 2009)

CTCAE v4.0, a new version of the CTEP, NCI CTC v3.0, includes Adverse Events applicable to all oncology clinical trials regardless of chronicity or modality.

Appendix 5 –Summary of Product Characteristics

Summary of Product Characteristics for:

Letrozole

5 Fluoro-uracile

Epirubicine

Cyclophosphamide

Docetaxel

are available from the links below :

http://agmed.sante.gouv.fr/

http://www.emea.eu.int/

http://www.vidalpro.net/

Appendix 6: Detailed Pathology Methods for Using Residual Cancer Burden (RCB)

Residual cancer burden (RCB) is estimated from routine pathologic sections of the primary breast tumor site and the regional lymph nodes after the completion of neoadjuvant therapy. Six variables are included in a calculation formula. The calculated RCB index value can also be categorized as one of four RCB classes. The calculation formula and detailed description can be found at a dedicated Web site:

http://www.mdanderson.org/breastcancer_RCB .

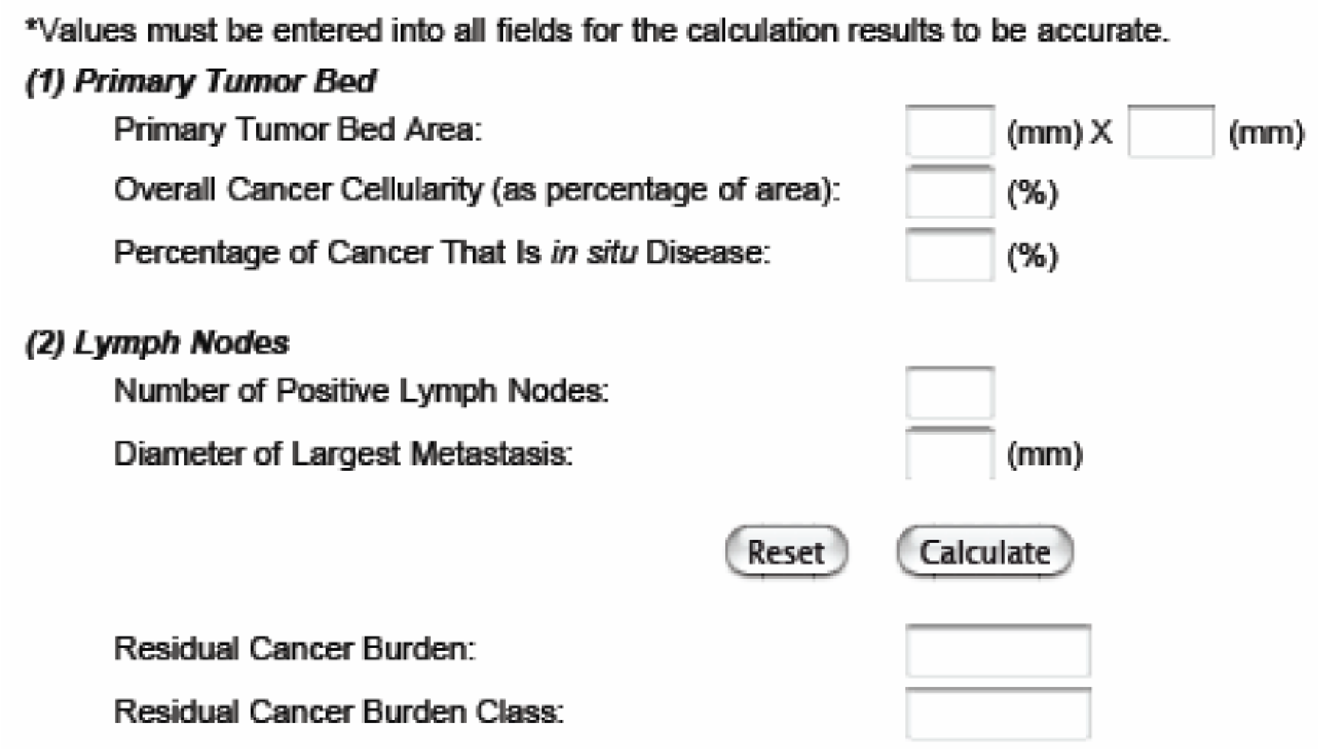
*Values must be entered into all fields for the calculation results to be accurate.

(1) Primary Tumor Bed

Primary Tumor Bed Area: (mm) X (mm)

Overall Cancer Cellularity (as percentage of area): (%)

Percentage of Cancer That Is *in situ* Disease: (%)

(2) Lymph Nodes

Number of Positive Lymph Nodes:

Diameter of Largest Metastasis: (mm)

Reset Calculate

Residual Cancer Burden:

Residual Cancer Burden Class:

Relevant information can be included within a pathology report (diagnoses or comment) without need for reporting calculated RCB index results. An example of relevant information from a report would be:

• Residual invasive carcinoma with chemotherapy effect

• Residual carcinoma measures 2.4 x 1.8 cm and contains approximately 10% cancer cellularity

• Residual intraductal carcinoma, solid type with necrosis, comprising 5% of the residual carcinoma

• Metastatic carcinoma involving three of 14 axillary lymph nodes (3/14)

• The largest metastasis measures 4 mm in greatest dimension

From the results above, one could calculate RCB using these results: d1 = 24 mm, d2 = 18 mm, %CA = 10%, %CIS = 5%, LN = 3, dmet = 4 mm.

Primary Tumor Bed: In general terms, pathologic evaluation of the primary tumor bed in the breast

requires that the pathologist make three judgments about the primary tumor bed:

i. Identify the cross-sectional dimensions of the residual tumor bed (d1 and d2),

ii. Estimate of the proportion of that residual tumor bed area that is involved by cancer (%CA),

and

iii. Estimate the proportion of the cancer that is in situ component (%CIS).

Defining the Tumor Bed.

In cases of multicentric disease, the RCB measurements are from the largest residual tumor bed. In cases where the extent of residual cancer under the microscope does not correlate with the gross measurement of the residual tumor bed, the tumor bed dimensions are to be revised according to the microscopic findings. Schematic diagrams are shown below to illustrate how gross residual tumor bed dimensions are first estimated from the gross findings (pink area) but may be revised after review of the slides from the gross tumor bed area according to the extent of residual cancer (blue).

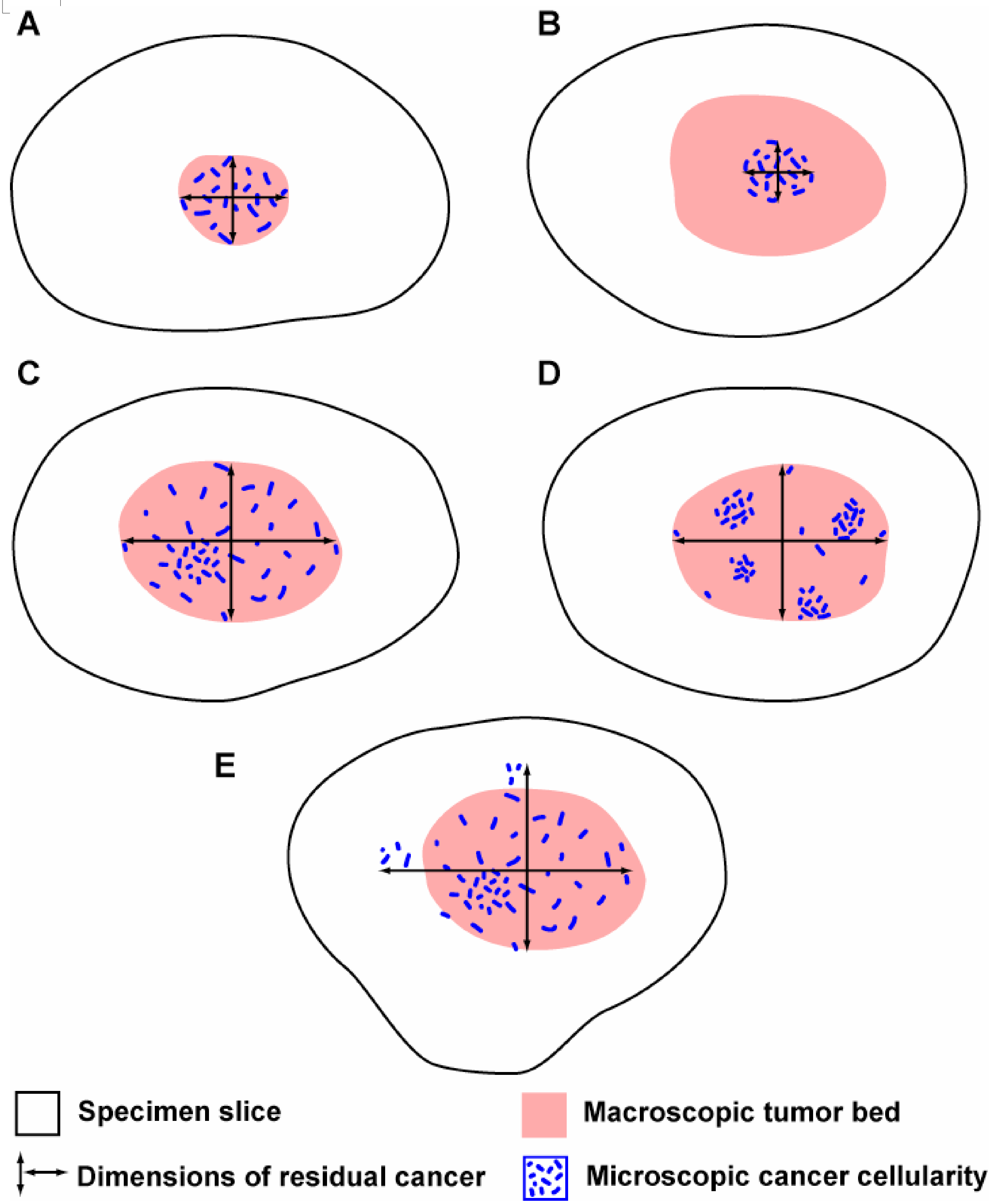

In these diagrams, the macroscopic tumor bed dimensions in examples A, C, D also define the final

dimensions of the residual tumor bed after microscopic review. However, the macroscopic tumor bed dimensions in example B overestimate the extent of residual cancer, and so the dimensions of the residual tumor bed (d1 and d2) would be revised after microscopic evaluation of the extent of residual cancer in the corresponding slides from the gross tumor bed. In a different example (E), microscopic residual cancer extends beyond the confines of the macroscopic tumor bed. Again, the dimensions of the residual

tumor bed (d1 and d2) would be revised after microscopic evaluation of the recognizable extent of residual cancer beyond the macroscopic tumor bed.

This approach accounts for differences in the concentration and distribution of residual cancer within a tumor bed. In the illustration above, the estimated %CA in example A would be high (in a small area), whereas the estimated %CA for examples C and D would be lower (in a larger area). In examples C and D, the estimated %CA would likely be similar, even though the distribution of cancer within the residual tumor bed is different in those two examples.

Estimating Cellularity within the Tumor Bed

The proportion of cancer (%CA) and the proportion of in situ component (%CIS) are estimated from

microscopic evaluation of the slides from the residual tumor bed area. The most effective way to obtain this information is to measure and submit for histology the largest cross-sectional area of residual tumor bed, and to designate in the report which slides represent the cross section of tumor bed. After reviewing those slides, the pathologist can estimate the average cellularity in the tumor bed on each slide in order to estimate the overall average cellularity of the tumor bed area (illustrated below).

The key is to simply:

i. Define the gross tumor bed as the largest cross-sectional area.

ii. Submit sections representing that tumor bed area as individual slides.

iii. Review those slides to estimate the %CA and %CIS within the residual tumor bed.

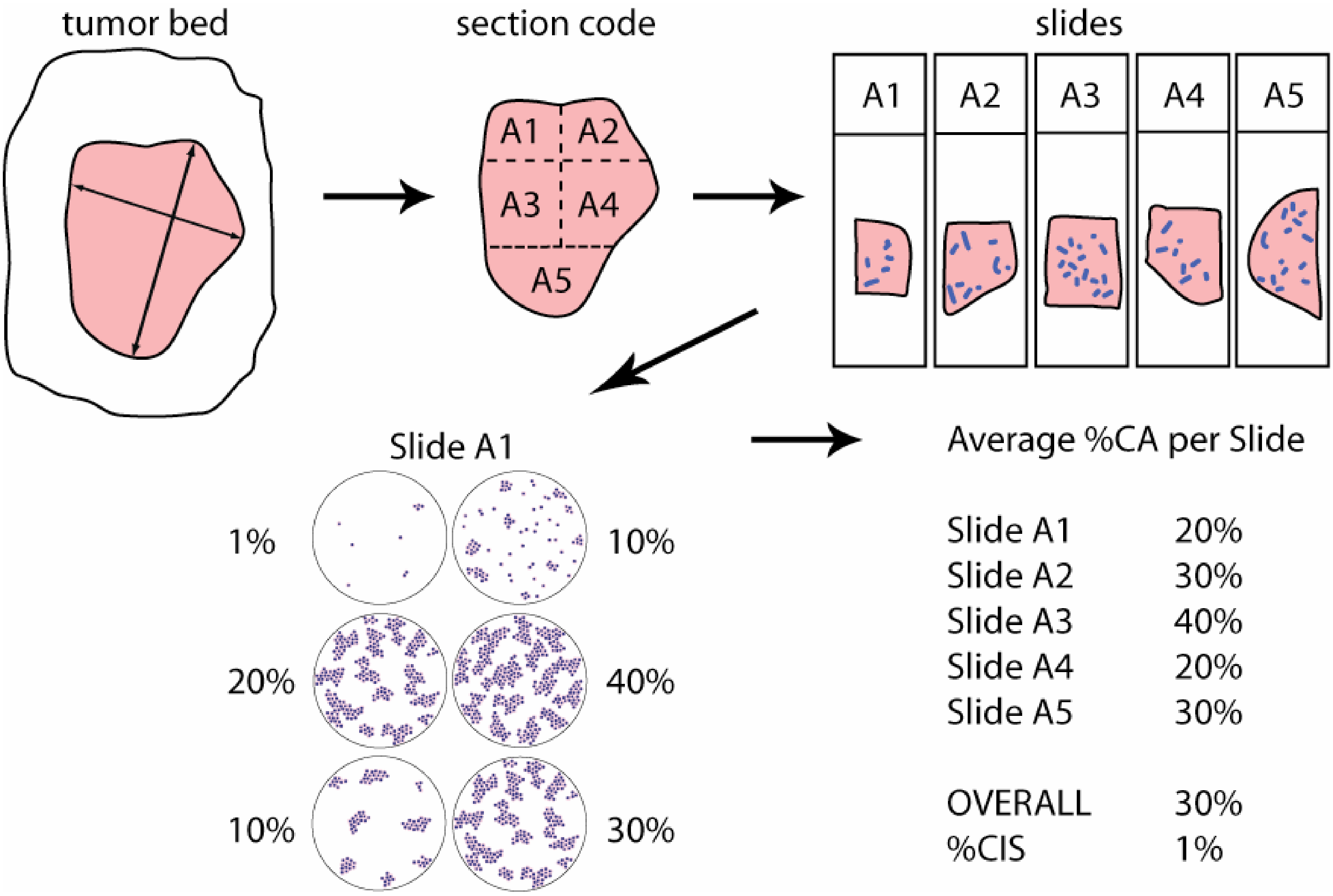

A practical way to estimate %CA in a slide is to encircle with ink dots the tumor bed on each slide from the grossly defined residual tumor bed (e.g., slides A1-A5 in the example above). Then use the microscope to estimate the cellularity in each microscopic field across the area of tumor bed. In each microscopic field, %CA can be estimated by comparing the proportion of residual tumor bed area containing cancer (invasive or in situ). Estimate an average of the readings for %CA in the cross-sectional area. The same can be done for in situ component (%CIS). Estimates are to the nearest 10%, but include 0%, 1%, and 5% for areas with low cellularity. The average cellularity within the tumor bed from each slide across the tumor bed can then be estimated (illustrated above). The website contains computergenerated diagrams of % cellularity per area to assist pathologists to estimate accurately the cellularity of a microscopic field. Those diagrams are appended at the end of this document.

Regional Lymph Nodes: Pathologic evaluation of the primary tumor bed in the breast requires that the pathologist make two judgments:

i. Count the number of positive lymph nodes (LN).

ii. Measure the diameter of the largest nodal metastasis (dmet).

Footnotes

Inoperable or Progressive Disease

The RCB index cannot be accurately calculated for patients whose disease remains inoperable at the completion of the neoadjuvant treatment course (e.g., requiring subsequent additional treatments before surgical resection is possible), or those who experience disease progression and so do not undergo surgical resection at the completion of the neoadjuvant treatment course. For those patients, RCB is assigned as extensive, i.e., RCB-III.

Internal Mammary Lymph Node Metastasis

There were no examples of internal mammary nodal metastasis in the published study that evaluated the prognostic value of RCB. However, it is reasonable to include internal mammary nodes with the other regional (axillary) nodes in the assessment of RCB.

Pre-treatment Sentinel Lymph Node Biopsy

Surgical excision of a positive sentinel lymph node before the neoadjuvant treatment would invalidate the accuracy of measuring RCB after the treatment to assess response. If all sentinel lymph nodes were negative before treatment began, this would not affect the assessment of RCB after treatment ended.

Summary of Key Points for Pathologic Assessment of the Primary Tumor Bed

Define the dimensions of residual tumor bed and estimate the percent of that area that is cancer.

1. GROSS. Identify the residual tumor bed and describe this macroscopic finding:

a. Report the measurements of the largest gross dimensions (prefer three dimensions, but minimum is two dimensions).

b. Submit the largest cross-sectional area for histology and specifically describe those blocks in the Section Code:

i. Try to indicate how they are oriented by photography, or a scheme or intelligent description (e.g., "blocks B1 – B7 cross section of tumor bed in rows from antero-superior to postero-inferior").

ii. If additional blocks are from surrounding tissues, then describe those as well.

iii. Five representative sections from a big, obvious tumor bed should be sufficient.

2. **MICROSCOPY**. Review the slides that correspond to the tumor bed (+/- surrounding tissues):

a. Estimate the extent of spread of residual cancer relative to the gross tumor bed:

i. If similar to the gross description, then keep the original measurements.

ii. If obviously different, then revise the dimensions of the tumor bed based on the microscopic review of the tumor bed.

iii. Suggestion: Dotting the perimeter of cancer in each slide can be helpful to reconstruct the tumor extent across multiple slides (see point 1-b-i).

b. Using the microscope, make visual snapshots of cancer cellularity as you go from field to field across the defined tumor bed from one end to the opposite (e.g., left to right, then top to bottom) to estimate the:

i. Average cancer cellularity (%) across the entire tumor bed. Considering both invasive and in situ components.

ii. Average percent of the cancer within the tumor bed that is in situ.

iii. Cellularity estimates are to the nearest 10%, with additional selections of 1% and 5% for very low cellularity. For reference, there are images of computer generated examples linked to the Web site: http://www.mdanderson.org/breastcancer_RCB.

iv. The usual misunderstanding is to only make estimates in foci of the tumor bed that contain lots of cancer. The estimates are supposed to represent the average across the entire residual tumor bed area

Regional Lymph Nodes: Pathologic evaluation of the primary tumor bed in the breast requires that the pathologist make two judgments:

i. Count the number of positive lymph nodes.

ii. Measure the diameter of the largest nodal metastasis.

Graphical Illustrations of Percentage Cancer Cellularity

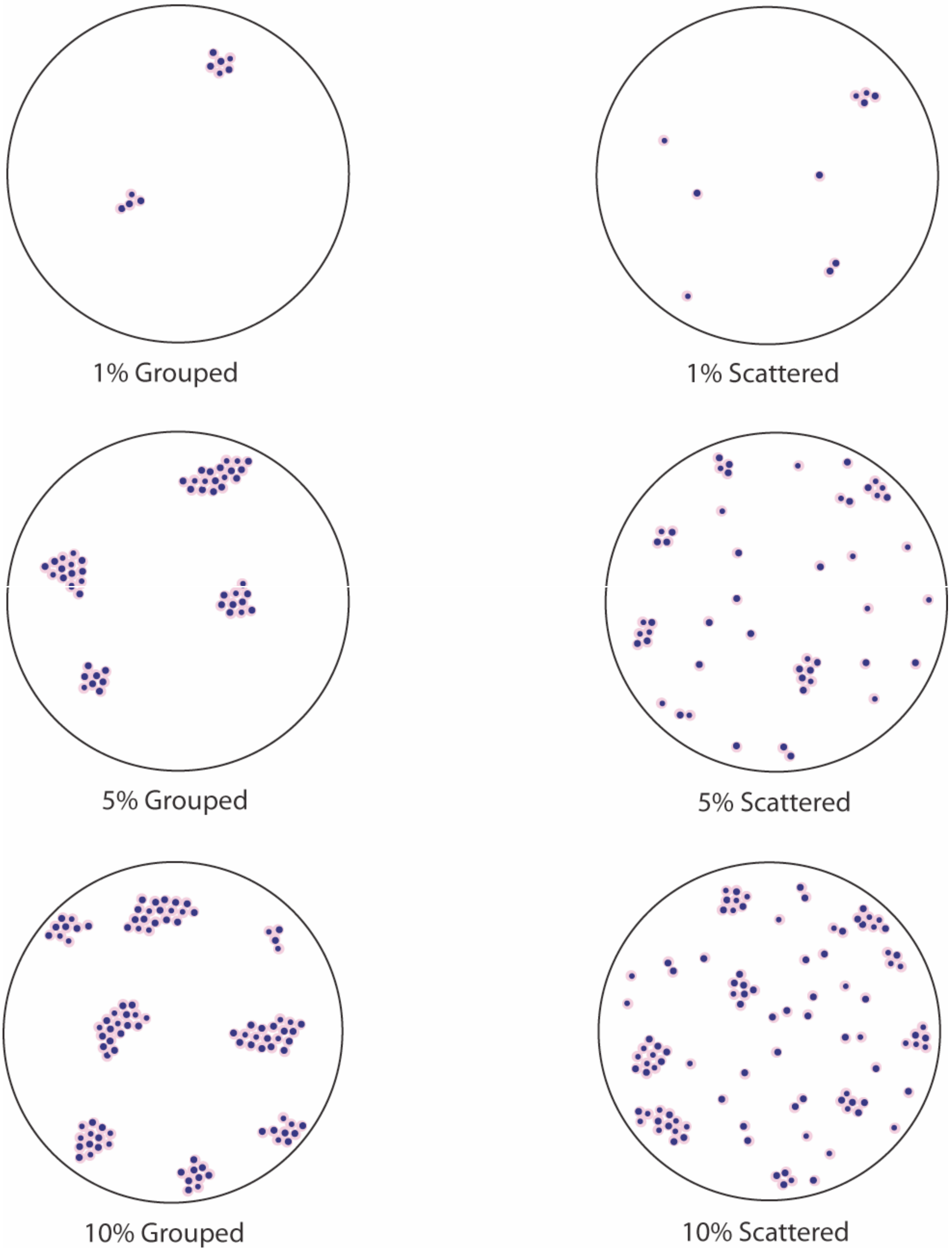

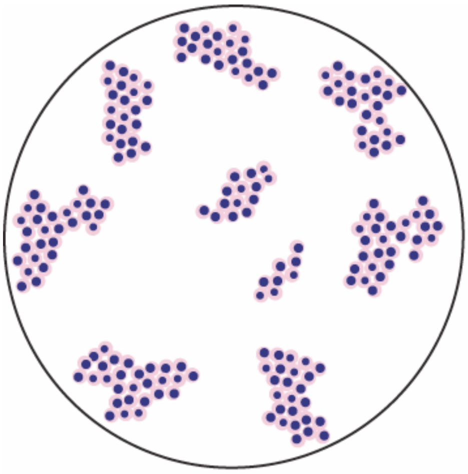

20% Grouped

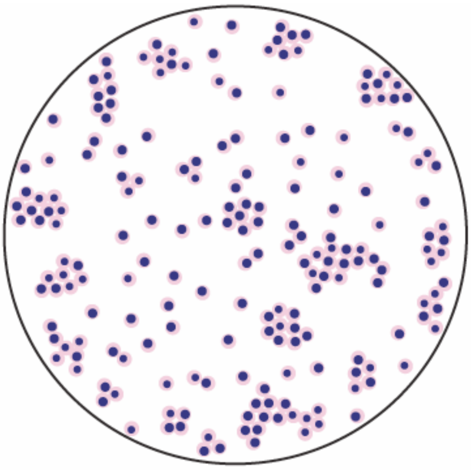

20% Scattered

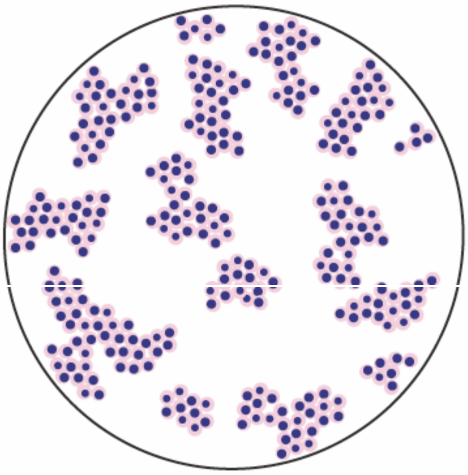

30% Grouped

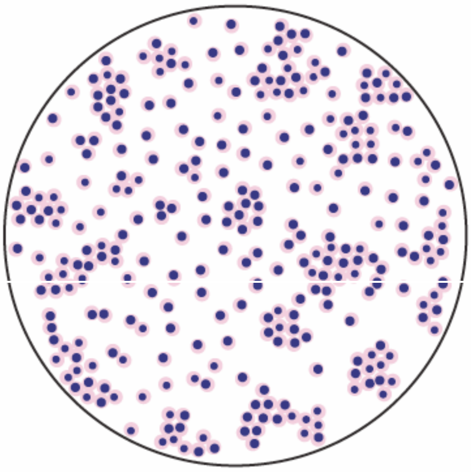

30% Scattered

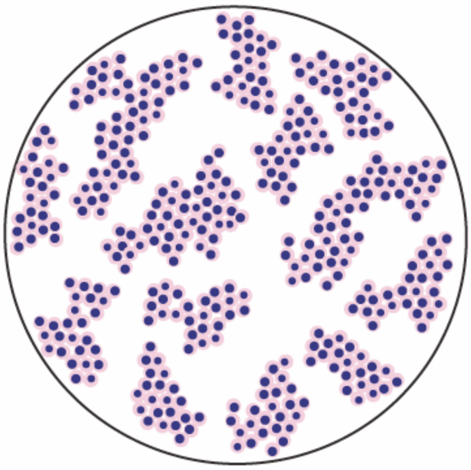

40%

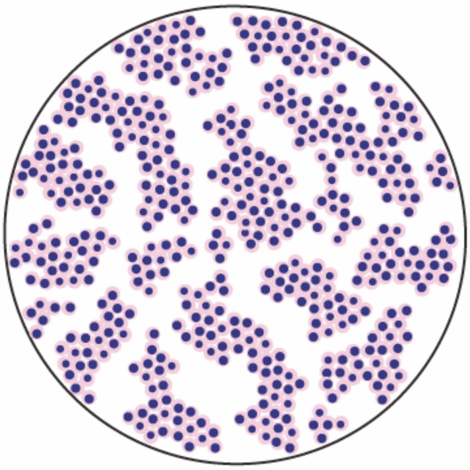

50%

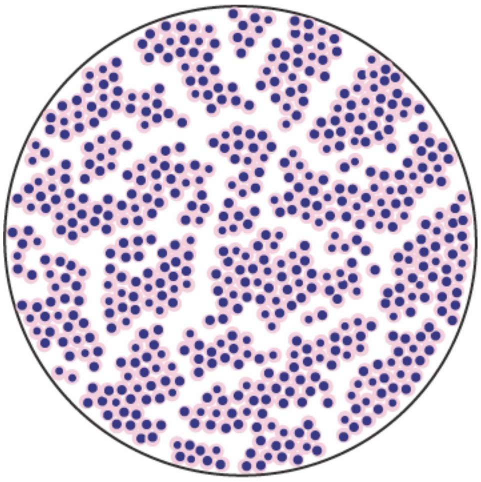

60%

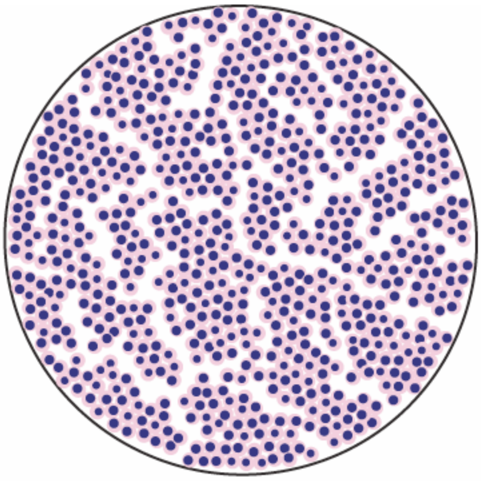

70%

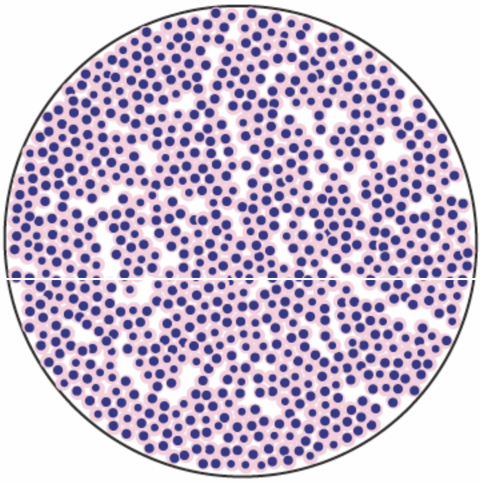

80%

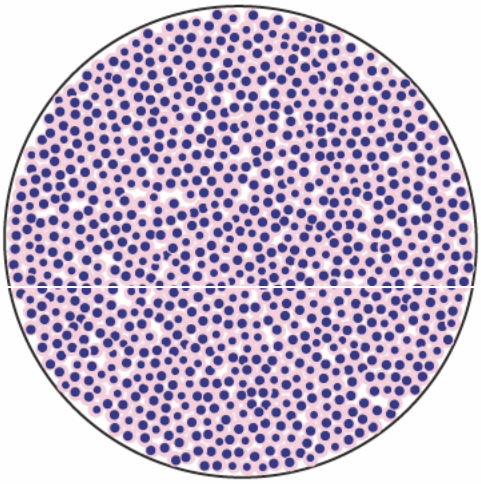

90%

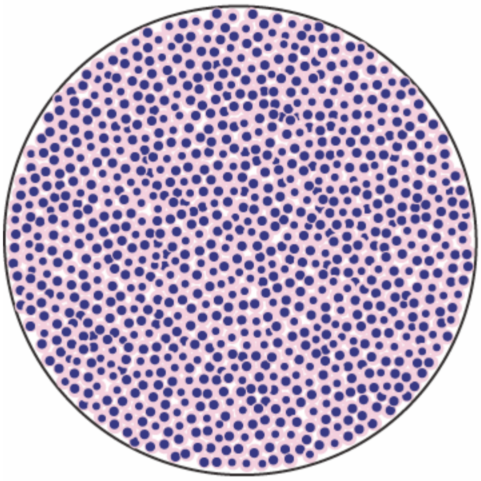

95%

DETAILS OF THIS MODEL

Area of circle = 2,827.8 mm^2

Area of 1 cell = 2.8278 mm^2

1 cell = 0.1% of Area = 0.1% cellularity

10 cells = 1% cellularity

1000 cells = 100% cellularity

Appendix 7: Scheme of logistic flow for pathological samples

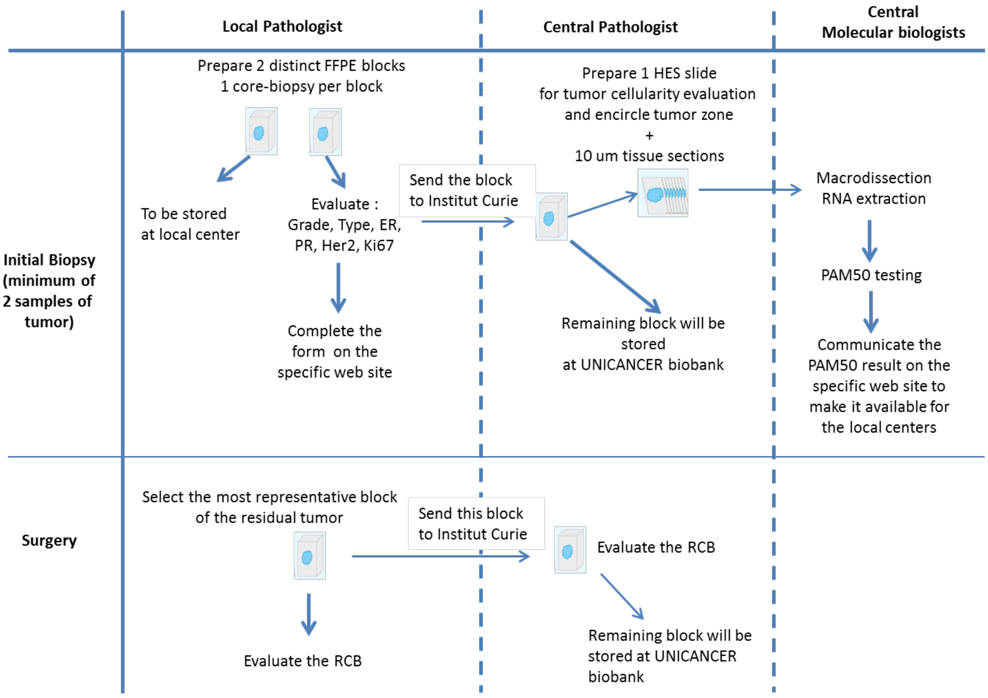

Supplement: Document S1. Figures S1–S6 and Tables S1–S3, S5, and S6 [file mmc1.pdf]
